# Supplementary material for: Cyclic 5-membered disulfides are not selective substrates of thioredoxin reductase, but are opened nonspecifically
Source: Nat Commun. 2022 Apr 1;13:1754. doi: 10.1038/s41467-022-29136-4 (PMC8975869; doi:10.1038/s41467-022-29136-4)
Supplement: Supplementary file 1 — Supplementary Information [file 41467_2022_29136_MOESM1_ESM.pdf]

Supplementary Information to:

Cyclic 5-membered disulfides are not selective substrates of thioredoxin reductase, but are opened nonspecifically

Jan G. Felber[1], Lena Poczka[1], Karoline C. Scholzen[2], Lukas Zeisel[1], Martin S. Maier[1], Sander Busker[2,#], Ulrike Theisen[3], Christina Brandstädter[4], Katja Becker[4], Elias S. J. Arnér[2,5], Julia Thorn-Seshold[1], Oliver Thorn-Seshold[1,*]

[1] Department of Pharmacy, Ludwig-Maximilians University Munich, Butenandtstr. 5-13, 81377 München, DE.
[2] Department of Medical Biochemistry, Karolinska Institutet, Solnavägen 9, 17177 Stockholm, SE.
[3] Zoological Institute, Cellular and Molecular Neurobiology, TU Braunschweig, Spielmannstr. 7, 38106 Braunschweig, DE.
[4] Interdisciplinary Research Centre (IFZ), Justus-Liebig University Giessen, Heinrich-Buff-Ring 26-32, 35392 Giessen, DE.
[5] Department of Selenoprotein Research, National Institute of Oncology, 1122 Budapest, HU.

[#] Present affiliation: Pelago Bioscience AB, 171 48 Solna, Sweden.

* Correspondence to: oliver.thorn-seshold@cup.lmu.de

1 Supplementary Discussion **4**

1.1 Summary: TrxR selectivity vs. thiol-mediated uptake **4**

1.2 1,2-dithiolane-based probe strategies **6**

1.3 Cell-free evaluation of reduction-mediated release: methods *7*

1.4 Cell-free enzyme specificity/activity studies *8*

1.5 Cellular TrxR suppression, knockdown, knockout and knockin *11*

1.6 Supplementary Note 1: 1,2-dithiolanes; and PAINS **12**

1.7 Cell-free and cellular studies with electrophilic inhibitors *15*

1.8 Supplementary Note 2: Auranofin's polypharmacology **21**

1.9 Lipid vesicle-mediated ROP of Fast-TRFS *23*

1.10 Supplementary Note 3: Fast-TRFS' TrxR-independent fluorogenicity **24**

1.11 Supplementary Note 4: TRFS-green's TrxR-independent fluorogenicity **25**

1.12 In vivo animal model, zebrafish: results and impact *26*

1.13 Supplementary Movie Caption *28*

2 Supplementary methods **28**

2.1 Methods for in vitro evaluation *28*

2.2 Cell culture methods *29*

2.3 Methods for in vivo animal model *30*

2.4 Laboratory techniques *31*

3 Synthetic Procedures **32**

4 NMR Spectra **38**

5 Supplementary References **45**

Abbreviations

AF	auranofin
Boc	*tert*-butoxycarbonyl
CA	cysteamine
COSY	correlated spectroscopy
Cys	*L*-cysteine
DAD	diode array detector
DCM	dichloromethane
DDQ	2,3-dichloro-5,6-dicyano-1,4-benzoquinone
δ	chemical shift (ppm)
DIAD	diisopropyl azodicarboxylate
DIPEA	diisopropylethylamine
DMEM	Dulbecco's modified eagle medium
DMF	dimethylformamide
DMSO	dimethylsulfoxide
dpf	days post fertilization
DTT	dithiothreitol
EC_{50}	half maximal effective concentration
EI	electron ionisation
ESI	electron spray ionisation
ESIPT	excited-state intramolecular proton transfer
ETP	epidithiodioxopiperazine
ex/em	excitation/emission
FACS	fluorescence activated cell scanning
Fast-TRFS	1-(1,2-dithiolan-4-yl)-3-(2-oxo-4-(trifluoromethyl)-2H-chromen-7-yl)urea
FBS	fetal bovine serum
GFP	green fluorescent protein
GR	glutathione reductase
Grx	glutaredoxin
GSH	glutathione (reduced form)
GSSG	glutathione disulfide
HeLa	Henrietta Lacks cervical cancer cell line
HEPES	2-[4-(2-hydroxyethyl)piperazin-1-yl]ethanesulfonic acid
HMBC	heteronuclear multiple bond correlation
HPLC	high-pressure liquid chromatography
hpt	hours post treatment
HRMS	high-resolution mass spectrometry
HSAc	thioacetic acid
HSQC	heteronuclear single quantum coherence spectroscopy
Linear-TRFS	1-methyl-1-(2-((2-morpholinoethyl)disulfaneyl)ethyl)-3-(2-oxo-4-(trifluoromethyl)-2*H*-chromen-7-yl)urea
MEDA	*N*,*N*-dimethylcysteamine
MEF	mouse embryonic fibroblasts
MsCl	methanesulfonyl chloride

NAC	*N*-acetyl *L*-cysteine
NADPH	*β*-nicotinamide adenine dinucleotide phosphate
NMR	nuclear magnetic resonance
PBS	phosphate-buffered saline
PCR	polymerase chain reaction
PQ-OH	6-chloro-2-(5-chloro-2-hydroxyphenyl)quinazolin-4(3H)-one
Pr-SH	a general cellular protein monothiol
R_f	retention factor
ROP	Ring-opening polymerisation
RPMI	Roswell park memorial institute medium
Ser	*L*-serine
S-Gem	disulfide-gemcitabine prodrug
SS50-PQ	4-chloro-2-(6-chloro-4-oxo-3,4-dihydroquinazolin-2-yl)phenyl (1,2-dithiolan-4-yl) (methyl)carbamate
TCEP	tris(2-carboxyethyl)phosphine
TE	Tris/EDTA buffer
THF	tetrahydrofuran
TRFS	TrxR fluorescent substrate probe (Fang *et al.*)
TRFS-green	1,2-dithiolan-4-yl (2-butyl-1,3-dioxo-2,3-dihydro-1*H*-benzo[*d,e*]isoquinolin-6-yl) carbamate
TRi	TrxR inhibitor (Busker *et al.*[1])
TRi-1	2-((4-chlorophenyl)sulfonyl)-6-methoxy-3-nitropyridine
TRi-3	4,5-dichloro-2-((5-(4-chlorophenyl)-1,3,4-oxadiazol-2-yl)methyl)pyridazin-3(2H)-one
TRP14	thioredoxin-related protein of 14 kDa
Trx	thioredoxin
TrxR	thioredoxin reductase

1 Supplementary Discussion

1.1 Summary: TrxR selectivity vs. thiol-mediated uptake

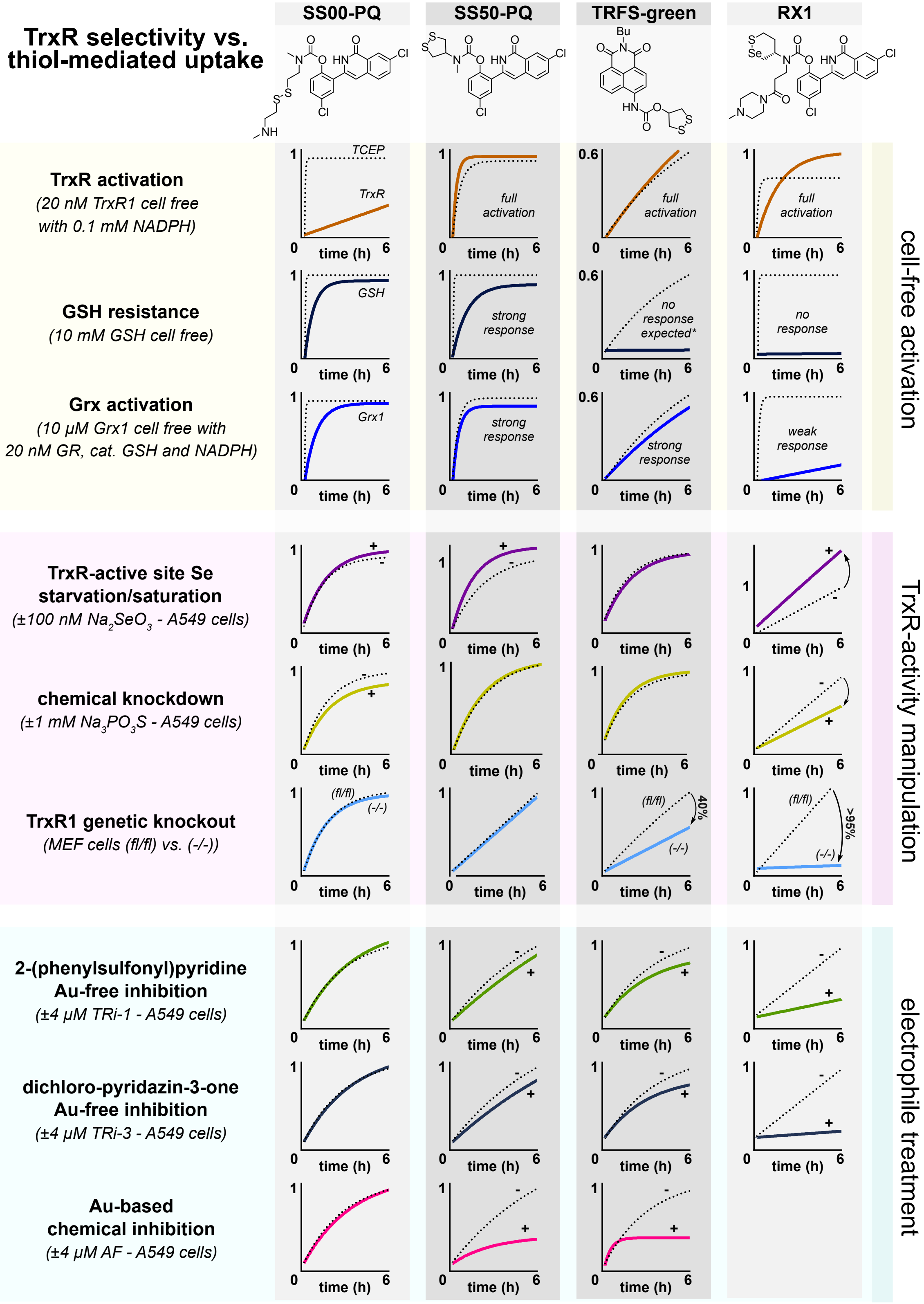

Supplementary Figure 1 Overview of major experimental results related to TrxR-(non)selectivity of 1,2-dithiolane-based probes cross-evaluated with TrxR-independent **SS00-PQ** and TrxR-dependent **RX1** in cell-free (at 10 µM) and in cellular settings (at 50 µM).

Discussion of Results Overview

Several features are apparent in **Supplementary Figure 1**, as discussed in the main text. Noteworthily, the strong cell-free responses with **SS50-PQ** show its nonselectivity for TrxR, while cellular TrxR activity manipulation experiments show that its processing is substantially independent of TrxR, leading to the conclusion that its pattern of cellular results in the electrophile treatments cannot be interpreted as TrxR selectivity. Therefore, **TRFS-green** which reproduces this identical pattern of cellular results in the electrophile treatments, and shows zero to low sensitivity to TrxR activity manipulation, and strong response to Grx in the cell-free assay, should not be interpreted as TrxR-selective either. This highlights the general sensitivity of 1,2-dithiolane towards a broad range of thiol reductants as well as confounding influences (see also vesicle experiments). Results for **RX1**[2], a redox probe which displays excellent cellular TrxR-selectivity, are provided for comparison to illustrate trends that would be expected for a TrxR-selective compound. The strong effects of auranofin on the non-TrxR-selective **SS50-PQ** and **TRFS-green** can be coherently interpreted as deriving from the effects of auranofin upon its targets other than TrxR, of which membrane thiol blocking is one. A dependency of cellular signal of the dithiolane-based cyclisation-driven release probes upon membrane thiol mediated, strain-promoted cellular uptake, is expected (see e.g. **Supplementary Figure 2**); consistent with this, **SS00-PQ** which does not benefit from strain-promoted thiol-mediated uptake is unaffected by auranofin.

*For the **TRFS-green** GSH data, see **Supplementary Figure 3c**.*

1.2 1,2-dithiolane-based probe strategies

a **1,2-dithiolane-mediated uptake probes**

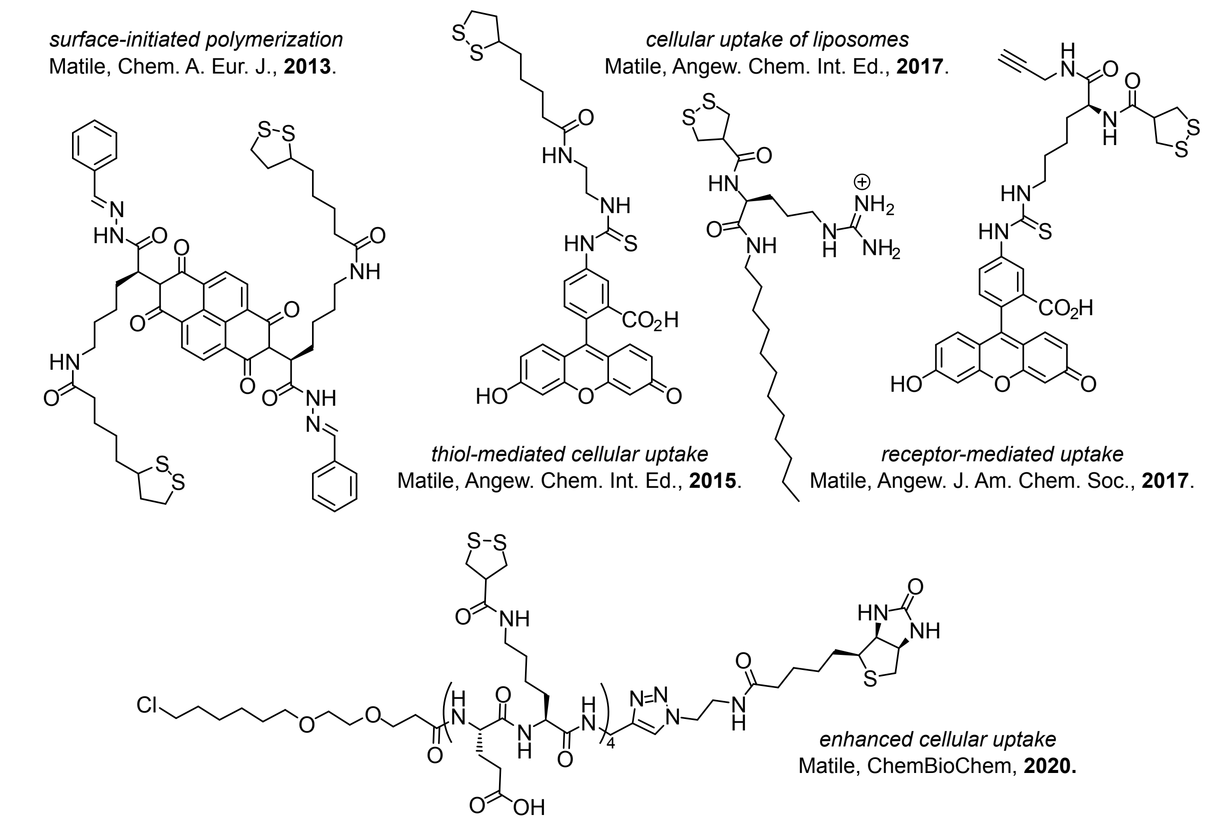

b **1,2-dithiolane-based TrxR probes**

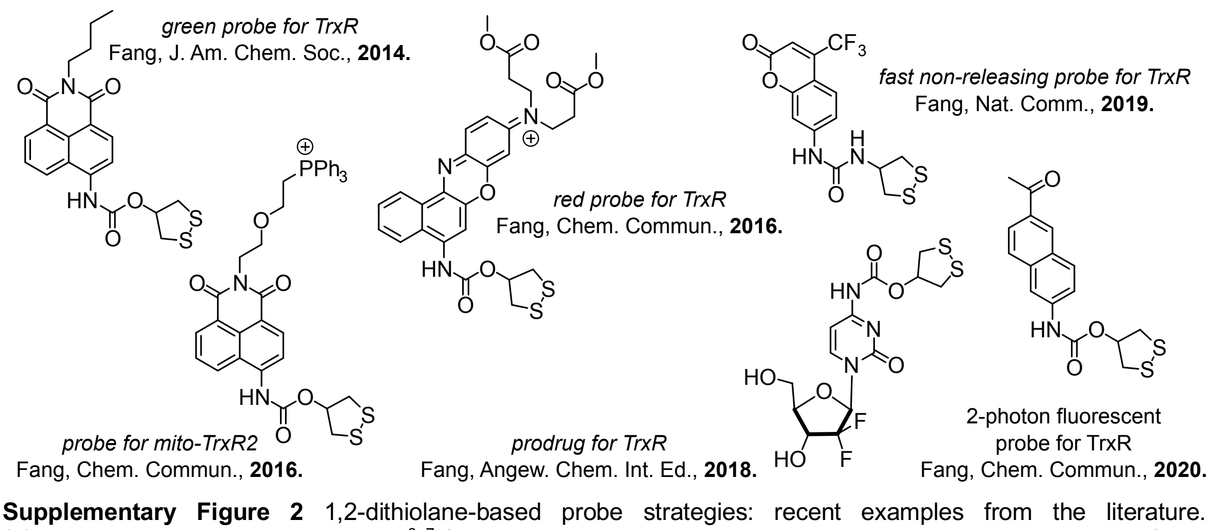

Supplementary Figure 2 1,2-dithiolane-based probe strategies: recent examples from the literature. (**a**) 1,2-dithiolanes are used by Matile[3–7] for strain-promoted thiol-mediated cellular uptake initiated by exofacial thiols, as well as for ring-opening polymerization (ROP) initiated by thiols or other nucleophiles; (**b**) 1,2-dithiolane is employed by Fang[8–13] as a reduction-sensing motif in cyclisation-driven release probes **TRFS-green**, **TRFS-red**, **mito-TRFS** and **TP-TRFS** and the prodrug **S-Gem** for TrxR activity, as well as in the dithiolane-opening-triggered fluorogenic probe **Fast-TRFS**.

1.3 Cell-free evaluation of reduction-mediated release: methods

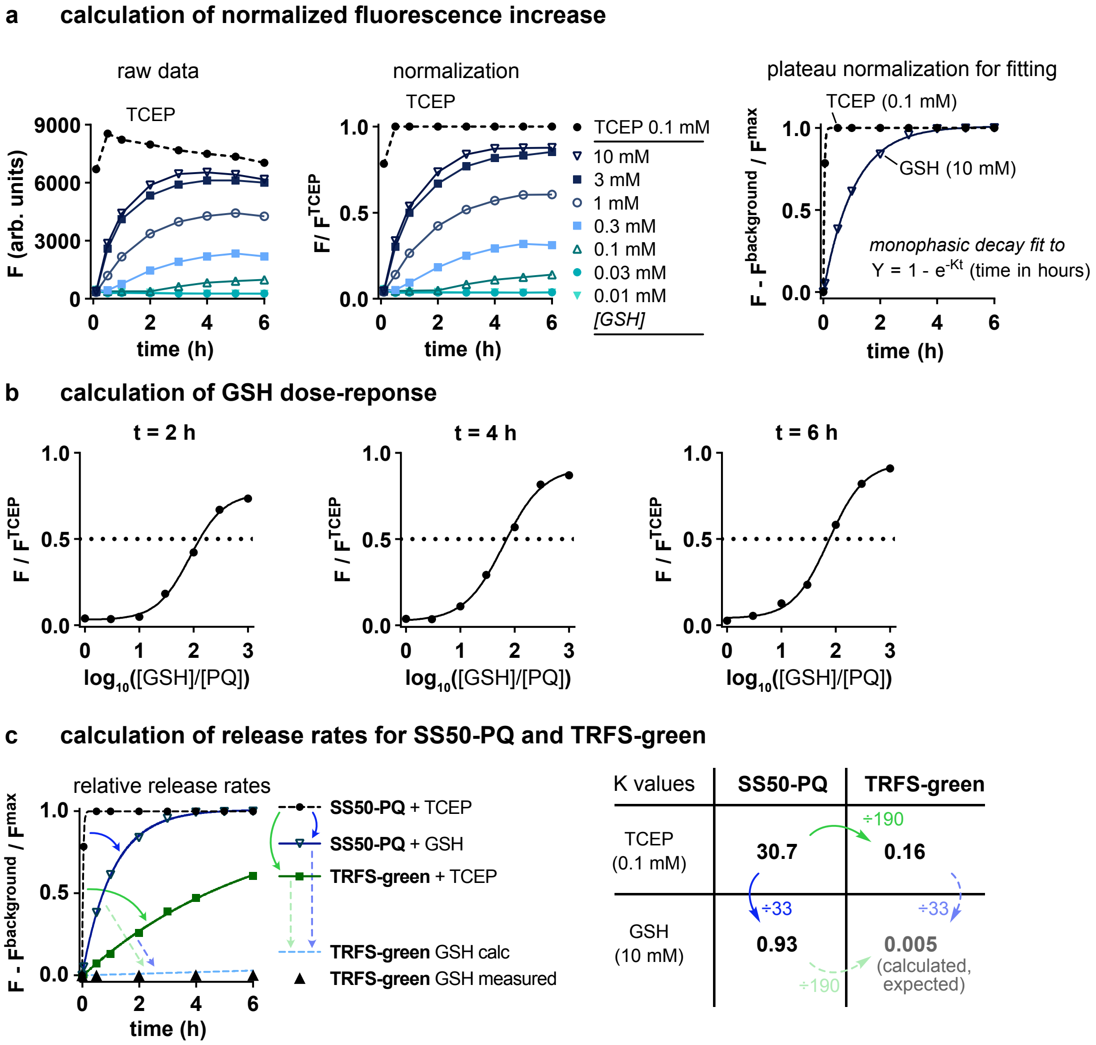

Supplementary Fig 3 Methods used in evaluation of *in vitro* performance of probes during reductant challenge. Example for **SS50-PQ** (10 µM) challenged with the monothiol reductant *L*-glutathione (GSH), monitoring reduction-mediated release of solid fluorescent **PQ-OH**. (**a**) Calculation of normalized fluorescence increase from raw fluorescence data recorded on a platereader (ex/em 355bp10/520lp) by reference to an experiment with maximum expected signal (TCEP at 100 µM). Only for the expected rate calculation in **Supplementary Figure 3c**, was the plateau of the GSH series adjusted to 1.0, for exponential fitting (assumes TCEP reduction near-instantaneous, so rate reports on cyclisation-elimination). (**b**) Dose-response curves for **SS50-PQ** upon challenge with GSH are very similar no matter what timepoint of data is used (t = 2 h, 4 h, 6 h): which is associated to the rapidity of post-reductive cyclisation-elimination of **SS50-PQ**. *Representative example out of 3 independent experiments.* (**c**) **TRFS-green** gives no significant fluorescence under GSH challenge in these assay conditions. However, it is ca. 190-fold slower to generate signal when treated with excess TCEP, than is **SS50-PQ**. Assuming that their reduction & cyclisation kinetics are identical, we interpret this as the expectedly slower kinetics of anilide vs phenolate leaving-group expulsion. Noting that GSH challenge of a 1,2-dithiolane is 33-fold slower to generate signal than TCEP challenge (**SS50-PQ** data), we can calculate the expected rate of signal generation under GSH challenge of **TRFS-green** either from its own TCEP rate, or from **SS50-PQ**'s GSH rate: but we arrive at a K-value of 0.005 regardless of which route is taken, which speaks to the accuracy of our rate assumptions.

The key outcome of this calculation is that no significant signal generation by **TRFS-green** can be expected in only 6 h of cell-free GSH challenge in homogenous media - *not because it is GSH-resistant*, but because its post-reduction kinetics are so slow that 6 h is insufficient to see much outcome. Contrasting this slow release rate to its instantaneous fluorogenicity in cellular experiments (e.g. early timepoints of **Supplementary Figure 11b**) supports that **TRFS-green** in cellular settings substantially gives signal through environment-dependent fluorogenicity of the trigger-cargo pair (whether as intact probe, or as e.g. a membrane-thiol-exchange adduct), and not through the very slow anilide elimination: therefore interpreting its fluorogenicity as reporting on post-reduction-elimination is therefore dangerous. It is an instructive exercise to calculate just what fraction of the reaction speed *of 10-fold excess of TCEP* that any given reductant (such as a 500-fold lower concentration of TrxR) would have to give, in

order to drive this slow probe to display any significant fluorescence increase, *unless effects such as environment-dependent fluorogenicity dominate the readout.*

Further discussions of rates relevant to signal generation are to be found in Felber *et al.*[14]

1.4 Cell-free enzyme specificity/activity studies

a calculation of normalized fluorescence increase

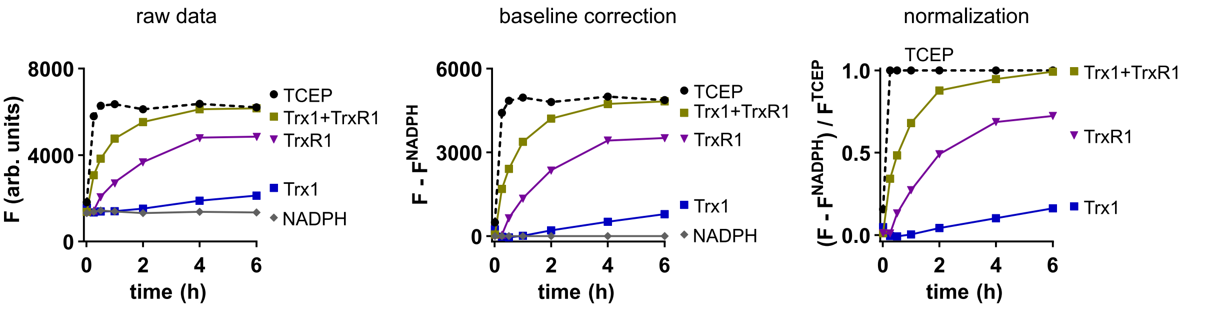

b probe activation by various redox cascades

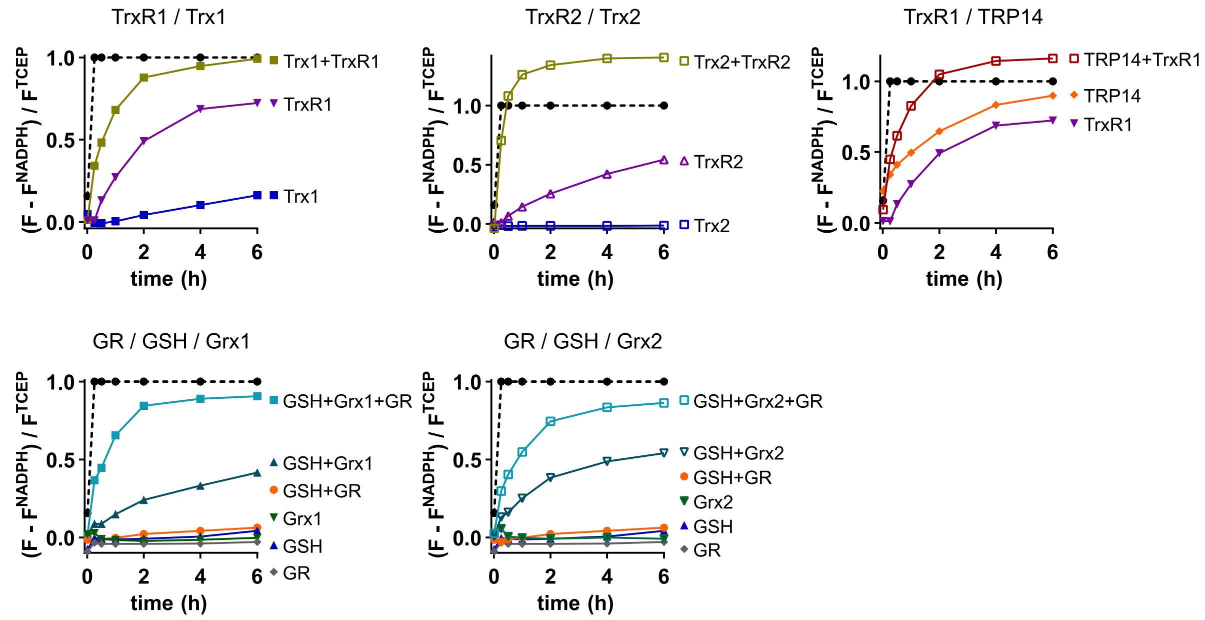

Supplementary Fig 4 Methods used in evaluation of *in vitro* performance of probes when treated with purified enzymes. Example for **SS50-PQ** (10 µM) treated with various members of different redox cascades, monitoring reduction-mediated release of solid fluorescent **PQ-OH**. (**a**) Example enzyme activity assay: calculation of normalized fluorescence increase from raw fluorescence data recorded on a platereader (ex/em 355bp10/520lp), corrected for the baseline autofluorescence of reduced β-NADPH, and normalised to a reference experiment with maximum expected signal (TCEP at 100 µM); (**b**) Resulting normalised data for probe activation by various members of the different redox cascades: (1) TrxR1 (20 nM), Trx1 (10 µM), TrxR1+Trx1; (2) TrxR2 (20 nM), Trx2 (10 µM), TrxR2+Trx2; (3) TrxR1 (20 nM), TRP14 (10 µM), TrxR1+TRP14; (4) GR (20 nM), GSH (10 µM), Grx1 (10 µM), GR+GSH, GR+Grx1, GSH+Grx1, GR+GSH+Grx1; (5) GR (20 nM), GSH (10 µM), Grx2 (10 µM), GR+GSH, GR+Grx2, GSH+Grx1, GR+GSH+Grx2. *Representative examples of 3 independent experiments.*

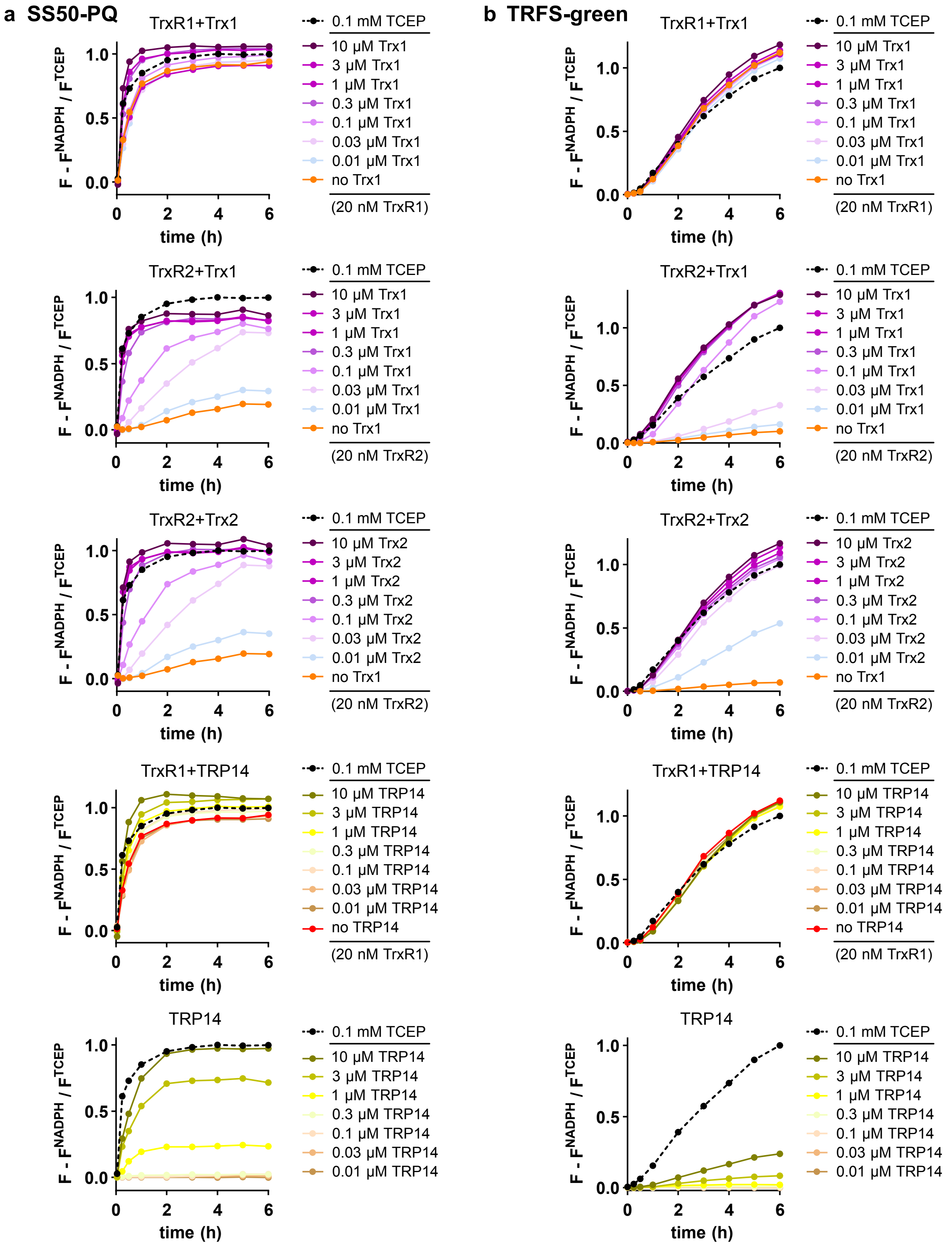

Supplementary Fig 5 Redox-effector protein titrations of the Trx-system challenging (**a**) **SS50-PQ** and (**b**) **TRFS-green** (10 µM). TrxR1 or TrxR2 (both at 20 nM) were employed with β-NADPH (100 µM) to recycle the redox effector proteins Trx1, Trx2 or TRP14 (from 0.01 µM to 10 µM); the "no Trx" results show that TrxR2 alone at this concentration is ineffective in reducing the probes, while significant direct reduction by TrxR1 at this concentration occurs that overlays the Trx-mediated reduction. Raw fluorescence data recorded on a platereader (ex/em 355bp10/520lp or 440bp10/520lp) is represented corrected by the basal fluorescence caused by autofluorescence of reduced β-NADPH if applicable and normalized to a reference experiment with maximum expected signal (TCEP at 100 µM). *Representative example out of 3 independent experiments.*

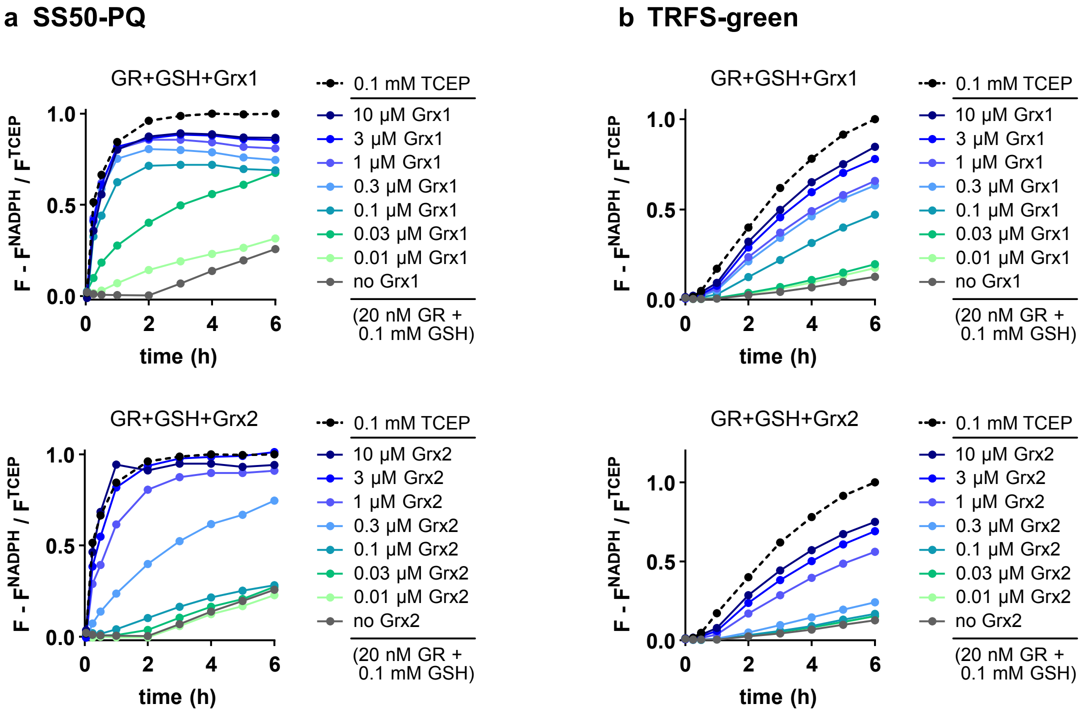

Supplementary Fig 6 Redox-effector protein titrations of the Grx-system challenging (**a**) **SS50-PQ** and (**b**) **TRFS-green** (10 μM). GR (at 20 nM) was employed with *β*-NADPH (100 μM) and used to recycle low concentrations of GSH (0.1 mM) and finally the redox effector proteins Grx1 or Grx2 (from 0.01 μM to 10 μM). Raw fluorescence data recorded on a platereader (ex/em 355bp10/520lp or 440bp10/520lp) is represented corrected by the basal fluorescence caused by autofluorescence of reduced *β*-NADPH if applicable and normalized to a reference experiment with maximum expected signal (TCEP at 100 μM). *Representative example out of 3 independent experiments.*

redox effector protein - dose-reponse

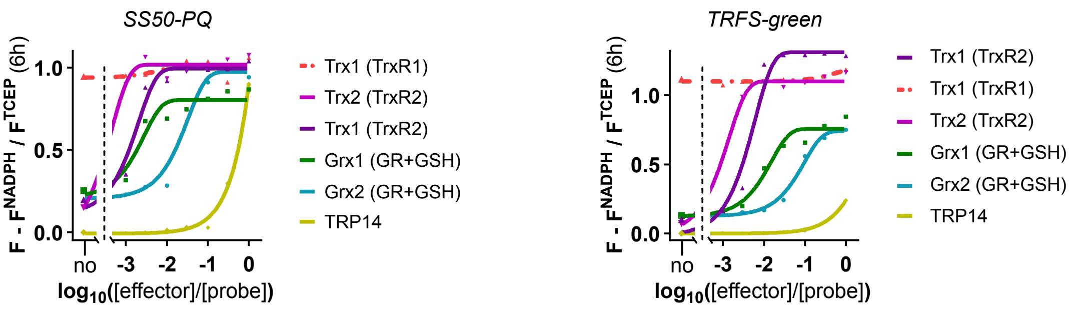

EC_{50} (nM)	Trx1 (TrxR2)	Trx2 (TrxR2)	Grx1 (GR+GSH)	Grx2 (GR+GSH)	TRP14*
**SS50-PQ**	1.4	0.3	1.9	22.9	9,016
**TRFS-green**	4.2	1.0	10.7	67.5	> 10,000

Supplementary Fig 7 Dose-response plots of **SS50-PQ** and **TRFS-green** (both at 10 μM) cross-evaluated against increasing redox effector protein concentrations (0.01-10 μM) at timepoint t = 6 h. TRP14*: TRP14 was titrated without the addition of *β*-NADPH and/or a reductase, as it can only be recycled efficiently by TrxR1 and TrxR1 has itself fast kinetics in reduction of both **SS50-PQ** and **TRFS-green.** *Representative example out of 3 independent experiments.*

1.5 Cellular TrxR suppression, knockdown, knockout and knockin

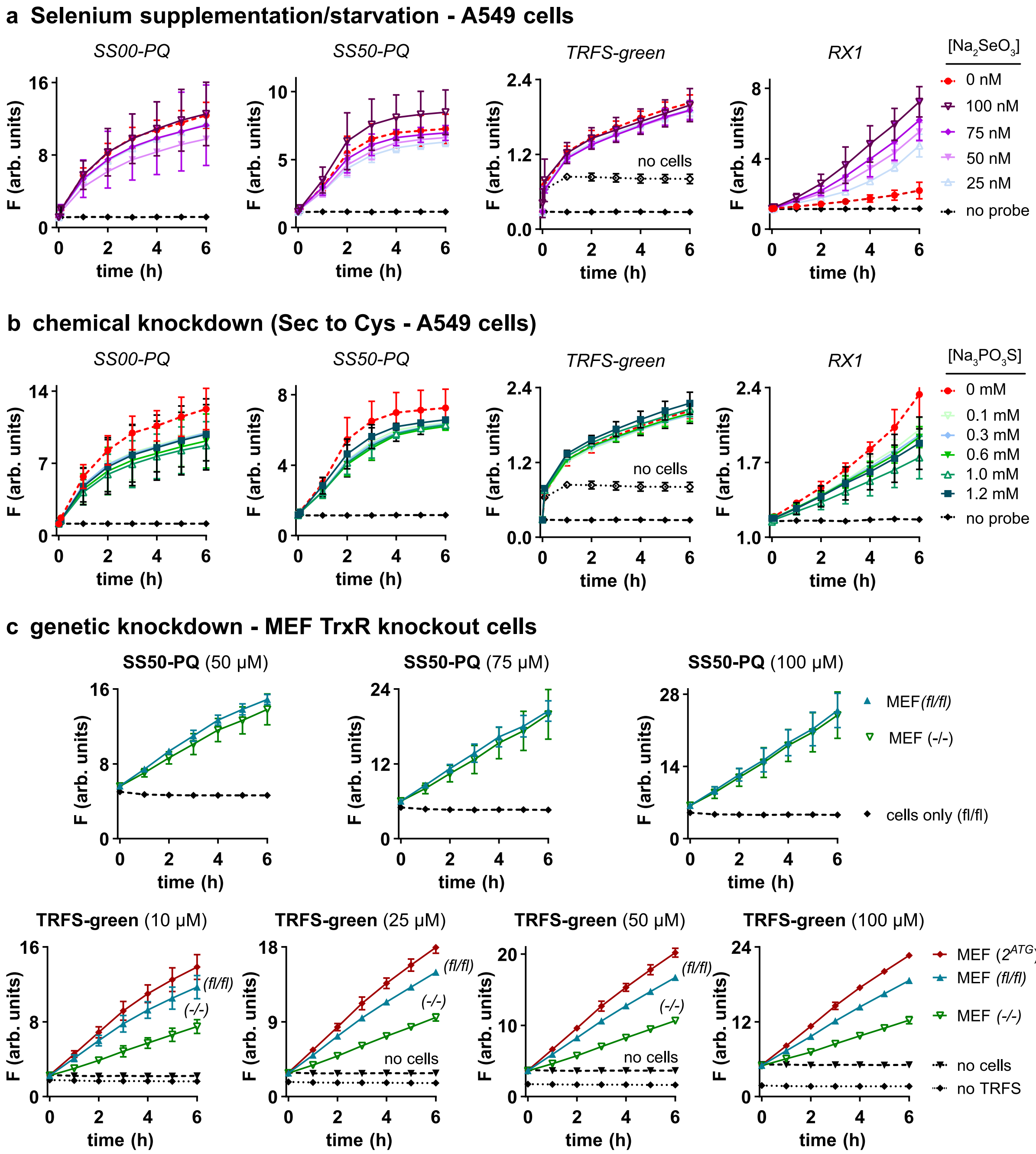

Supplementary Fig 8 Cellular tests of the TrxR-dependency of **SS50-PQ** and **TRFS-green**, with comparisons to the performance of positive control probe **SS00-PQ** and the TrxR1-selective probe **RX1**[2]. (**a**) A549 cells were supplemented with (**a**) Na_2SeO_3 (25-100 nM) (**b**) with Na_3PO_3S (0.1-1.2 mM) for 48 h, before treated with **SS00-PQ**, **SS50-PQ**, **TRFS-green** and **RX1** (all at 100 µM) and fluorescence was monitored (ex/em 355bp10/520lp or 390bp10/520lp) for 6 h. (**c**) Assay comparing fluorescence (ex/em 355bp10/520lp or 440bp10/520lp) timecourses in MEF cells with TrxR1 knockout (-/-)[15], to the precursor MEF cell line from which knockout was not performed (fl/fl), as well as to the 2^{ATG} cell line[16] where TrxR1 is knocked in. *All graphs in Supplementary Figure 8 show Mean+SD calculated from 3 independent experiments.*

Summary of TrxR modulation results

TrxR1 functional suppression (**Supplementary Figure 8a**), knockdown (**Supplementary Figure 8b**), or knockout (**Supplementary Figure 8c**), do not prevent signal generation from either probe. It should also be noted that all these TrxR1 modulations will also affect the redox status of all its downstream targets, both direct targets such as

Trx1 and indirect ones (targets of Trx1); that membrane-bound Trx1[17] is a significant species in cell-surface thiol chemistry; and that the fact that the **TRFS-green** signal saturates above 10 µM is consistent with the presence of a limiting mechanism for signal generation, such as rate of thiol-mediated uptake and concentration into water-excluded environment, being a determinant of cellular signal: but is not consistent with the linearity of dose-response seen for the faster-releasing environment-independent probe **SS50-PQ**. The interested reader can also revisit the discussion following **Supplementary Figure 3**, in which we argue based on rates of reaction with 10-fold excess of TCEP that the very slow release-based signal generation of **TRFS-green** makes it to our understanding impossible that this probe can hope to report cleanly on TrxR activity in cells, instead of giving a signal that is entirely dominated by environment-dependent effects.

1.6 Supplementary Note 1: 1,2-dithiolanes; and PAINS

Specific dithiol/disulfide-exchange reactions are central to biology, and engineered disulfides exploiting these reaction manifolds have applications from chemical biology probes to biophysics and materials chemistry. 1,2-dithiolanes have emerged as substrates of interest in chemical biology[8,11,12] (**Supplementary Figure 2**), although it has remained contentious whether its reduction is enzyme-selective, or nonspecific. In this paper we answered this question by studying the biochemical and biological performance of the novel 1,2-dithiolane-based redox probe **SS50-PQ**. The disulfide was integrated in a stable, modular design that can be adapted to release arbitrary phenols: in this case, a release-activated fluorescent cargo, that we could even use in flow cytometry studies and for cell-resolved imaging in live embryos.

A rigorous methodology of reductant titrations, enzyme panel screenings, and inhibitor/knockout cell experiments has here demonstrated the nonspecific reduction-based cleavage of the 1,2-dithiolane. While 1,2-dithiolane can indeed be rapidly opened by TrxR, particularly TrxR1 (**Supplementary Figure 4**), it is also rapidly opened by GSH at sub physiological concentrations (**Supplementary Figure 3**) as well as by many thiol protein reductants (**Supplementary Figures 5-7**). Therefore, inside cells, it is highly unlikely to selectively report on TrxR, instead of on more abundantly present reductants. The cellular data also show that neither TrxR1 knockdown, knockout, nor TrxR inhibition by recently developed Au-free inhibitors (**Supplementary Figures 8, 10-15**), greatly modulate the cellular signal obtained from the 1,2-dithiolane-based probes.

Note 1.1: Comparisons between the different cargo-releasing dithiolane probe designs

The same nonselectivity conclusions outlined above for **SS50-PQ** apply also to the other tested dithiolane probes. Particularly, for the irreversible cyclisation-driven-release probe **TRFS-green**:

(1) It is significant to show which reductants are capable of triggering fluorophore release in the cell-free setting. **Figure 3c-d** shows that the aniline-eliminating probe TRFS-green is kinetically labile to all tested redox effector proteins Trx1, Trx2, Grx1, Grx2 and TRP14, with very similar dose-response as the phenol-eliminating **SS50-PQ**, just with an apparent shift to higher reductant doses: although this shift arises not because of any difference of intrinsic reducibility (selectivity) of their 1,2-dithiolanes, but simply because anilines are poorer leaving groups. Therefore, we consider that the post-reduction-expulsion kinetics of **SS50-PQ** and **TRFS-green** are predictably different according to the nature of the chemical cargo (TCEP challenge half-life ca. 3 hours for aniline expulsion of **TRFS-green**, but only ca. 5 minutes for phenolate expulsion from the more sensitive and easier to interpret **SS50-PQ**). This similarity is supported by **Supplementary Figures 5-6**, and particularly by the full dose-responses and tabulated EC_{50} values of **SS50-PQ** compared to **TRFS-green** for all protein effectors (**Supplementary Figure 7**) showing there is a consistent 3-to-5-fold difference of the 6 hour fluorescence dose-response across all effectors. This seems a compelling reiteration that redox structure-activity relationships do exist: the 1,2-dithiolane is the reducible motif constant across both probes, the distant aniline/phenol cargo is the leaving group kept constant between probe series, the only way that reduction rates between the series can maintain the same ratios across different reductants is if the dithiolane motif itself dictates reactivity (i.e. the disulfide plays the key role in selectivity, not the cargo).

(2) We also take **TRFS-green** through cellular signal generation studies to show that its signal generation is independent from its claimed reductase TrxR, supporting our general conclusion that 1,2-dithiolane-based probes are not capable of cellular selectivity:

(2a) TrxR1 knockdown does not prevent **TRFS-green** signal generation in cells (**Figure 5, Supplementary Figure 8**) but only reduces it by ca. 40%. TrxR2, the other isozyme of TrxR, is only present in mitochondria, and is not expected to have a comparable expression level to TrxR1. Additionally, TrxR2 is also known to have lower turnover of small molecule substrates in general, and cell-free data show (**Supplementary Figure 5**) that it is almost entirely incapable of **TRFS-green** reduction. Therefore, at least ca. 60% of signal generation in cells by **TRFS-green** cannot be directly related to TrxR activity. Regarding the 40% drop, this can arise straightforwardly in probes that are partially processed by downstream reductants dependent on TrxR, such as Trxs, without this change indicating any direct reduction of the probe specifically by TrxR. By comparison, **SS50-PQ** is entirely unresponsive to TrxR1 knockout. The similarity of cellular behaviour of the two probe systems, which in cell-free settings are strikingly similarly reducible by the same broad range of redox effectors, should be kept in mind: it strongly suggests that

results from **SS50-PQ** should also apply to **TRFS-green**. We believe this indicates that the **SS50-PQ** environment-independent release-based probe has more straightforwardly revealed, that 1,2-dithiolane probe activation is not significantly due to direct reduction by TrxR1 in the cellular context.

(2b) Selenium supplementation/depletion has no effect on **TRFS-green** signal generation in cells, even though this controls the amount of active site selenolthiol TrxR (**Figure 5**, **Supplementary Figure 8**). Thiophosphate supplementation (**Figure 5**, **Supplementary Figure 8**) which likewise prevents selenocysteine incorporation, is also ineffective at reducing **TRFS-green** signal. Unsurprisingly to us, **SS50-PQ** performs identically in both these respects i.e. is similarly nonresponsive to changes in the amount of functional cellular TrxR. It can again be concluded that cellular reduction of 1,2-dithiolane-based probes does not dependent on correctly-expressed UC-containing TrxR. For comparison to how a TrxR-dependent probe should perform, **Figure 5** references results of the redox probe candidate RX1[2], that data indicate to be near-exclusively directly activated by TrxR1: e.g. the >90% signal suppression by TrxR1 knockout, the signal enhancement by selenium supplementation, and the signal reduction by thiophosphate treatment.

(3) We also introduce new comparisons between several probes to show how assay interpretation requires combining independent experiments. For example:

(3a) 1,2-dithiolane **SS50-PQ** is unaffected by cellular TrxR knockout (**Figure 5**, **Supplementary Figure 8**): so it is not significantly cellularly activated by TrxR: yet its signal is mildly suppressed by cellular treatment with chalcophilic S_NAr-based electrophiles such as TRi-1 and TRi-3 (**Figure 5**), and strongly suppressed by cellular treatment with the lipophilic Au(I)-based Lewis acid auranofin (AF), which has more than 20 attested targets (new **Supplementary Figure 10-15**). Thus, treating cells with these thiol/selenol-affine electrophiles and observing this suppression of cellular signal from a dithiolane probe, cannot be cited as a proof that that 1,2-dithiolane-based probe is a selective reporter of TrxR, since **SS50-PQ** provides a clear counterexample. Relevant to this, Matile's studies[4] have showed that 1,2-dithiolanes substantially rely on *free* exofacial thiols for cellular uptake. This uptake is inhibited by general thiol-reactive species of all tested chemotypes (including even other disulfides) typically even suppressing uptake to only 10% of normal. This provides a coherent explanation: these lipophilic electrophiles can suppress cellular 1,2-dithiolane signal generation by reacting with thiols on the cell surface, so substantially blocking the otherwise strain-promoted enhanced cellular uptake that dithiolanes can experience.

(3b) Next, the electrophile treatment data for 1,2-dithiolane **TRFS-green**, show almost identical signal suppression as **SS50-PQ** (**Figure 5**, **Supplementary Figure 11**). Having shown that the electrophile assay does not test *TrxR selectivity* (see above), it is still supported by literature that one would expect that these electrophiles should inhibit strain-promoted cellular uptake of the dithiolane **TRFS-green** very similarly as for the dithiolane **SS50-PQ**. The observation that the level of inhibition is so similar between **SS50-PQ** and **TRFS-green** again suggests that their chemical behaviour (rooted in their 1,2-dithiolane) is the same, i.e. that the signal of **TRFS-green** is likewise being inhibited by electrophilic blocking of cellular thiols, not from the effects those electrophiles also have by partially reacting with cellular TrxR (which is only one of their many cellular targets).

(3c) To counter-test these results, the linear disulfide probe **SS00-PQ** was used. This unstrained probe should not benefit from strain-promoted thiol-mediated uptake enhancement (which can be suppressed by electrophiles); and we as well as others have shown that linear disulfide probes are not selective for any particular cellular reductant - while they are reducible by TrxR, they are also reducible by the vastly more concentrated GSH, Trx, Grx, etc. The electrophile assay results are perfectly coherent with this expectation (**Figure 5**, **Supplementary Figure 12**): the electrophiles do not suppress signal from **SS00-PQ**, since the signal from this non-dithiolane probe is not limited by strain-promoted uptake.

This coherent intercomparison of results across multiple probes (**Supplementary Figure 13**), and the unity of results between the releasing probe **SS50-PQ** and the alternative releasing design **TRFS-green**, delivers strong evidence for the generality of the nonselectivity of 1,2-dithiolane as a reducible motif, and for the other effects we report.

Taken together, we conclude that 1,2-dithiolane is a nonspecifically reduced motif, that is not a TrxR-specific substrate. Beyond the dithiolane-relevant references in the introduction to this paper, we also refer the interested reader to excellent work by Lothrop, Ruggles and Hondal[18], which shows that even when the unique selenothiol *N*-terminal reaction centre of TrxR is deleted, 1,2-dithiolane can still be reduced, further weakening the argument that dithiolane has any intrinsic selectivity for TrxR.

Note 1.2: Misinterpreting Dithiolanes can lead to PAINS

In recent years, a range of 1,2-dithiolane-based TRFS probes have been reported as TrxR-specific substrates; they have been used as such for mechanistic validations during screening[12] and in disease characterisation.[9,19] These reports interpret cellular 1,2-dithiolane probe readouts as monitoring TrxR activity; but we contend that these reports may benefit from re-evaluation for alternative interpretation and application. We also caution that since 1,2-dithiolane-based probes such as **TRFS-green** are commercialised, they may continue to be used in assays without appreciation that their *cellular* readouts do not uniquely monitor TrxR-activity: however, research that

assumes they are TrxR-selective, risks false outcomes in the same ways that have been reviewed for pan-assay interference compounds.[20]

Indeed, the set of 13 compounds that was reported to be "validated" as TrxR inhibitors on the basis of comparison to a 1,2-dithiolane-based probe[12], consists entirely of hydrophobic PAINS compounds (9 Michael acceptors, 2 naphthoquinones, and 2 polyphenolic flavones) including the archetypical PAINS, curcumin[21] and myricetin[22]; while the 20 new inhibitors also reported included no single compound that is not a typical or known PAINS candidate (ortho- or para-quinones, Michael acceptors, catecholic polyphenols/flavonoids, and the known thiol-reactive PAINS disulfiram[23]). The cautionary results and stepwise methodology we report can also prevent mistakes in the future. Both will be useful for systematising redox probe development.

1.7 Cell-free and cellular studies with electrophilic inhibitors

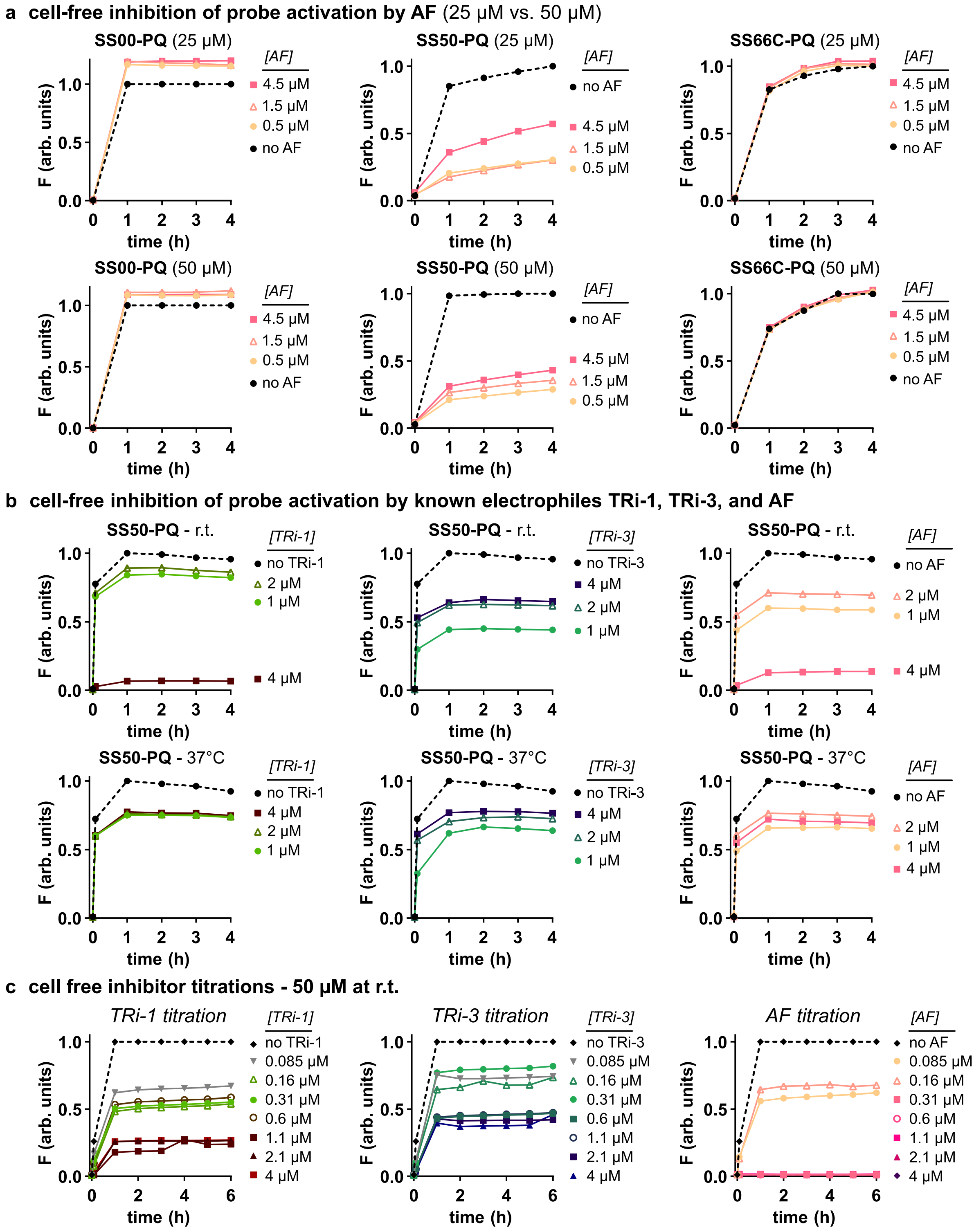

Supplementary Fig 9 Cell-free inhibition of potential reductive probe activation by electrophilic known TrxR-inhibitors was tested for **SS50-PQ** and referenced to non-cyclic **SS00-PQ** and non-strained **SS66C-PQ**[14] reference probes. Fluorescence timecourses for (**a**) **SS50-PQ**, **SS00-PQ** and **SS66C-PQ** (at 25 µM or 50 µM as indicated) pre-treated with AF (0.5-4.5 µM) for 2 h at r.t.; *then* treated with TCEP (at 100 µM). (**b**) **SS50-PQ** (at 50 µM) pre-treated with TRi-1, TRi-3 or AF (1.0-4.0 µM) for 2 h at r.t. or 37 °C (as indicated); *then* treated with TCEP (at 100 µM) (**c**) **SS50-PQ** (at 50 µM) pre-treated with TRi-1, TRi-3 or AF (0.085-4.0 µM) for 2 h at r.t.; *then* treated with TCEP (at 100 µM). *(**a-c**) show single experiments with technical replicates.*

a **SS50-PQ** (25 µM) **- inhibitor studies - A549 cells**

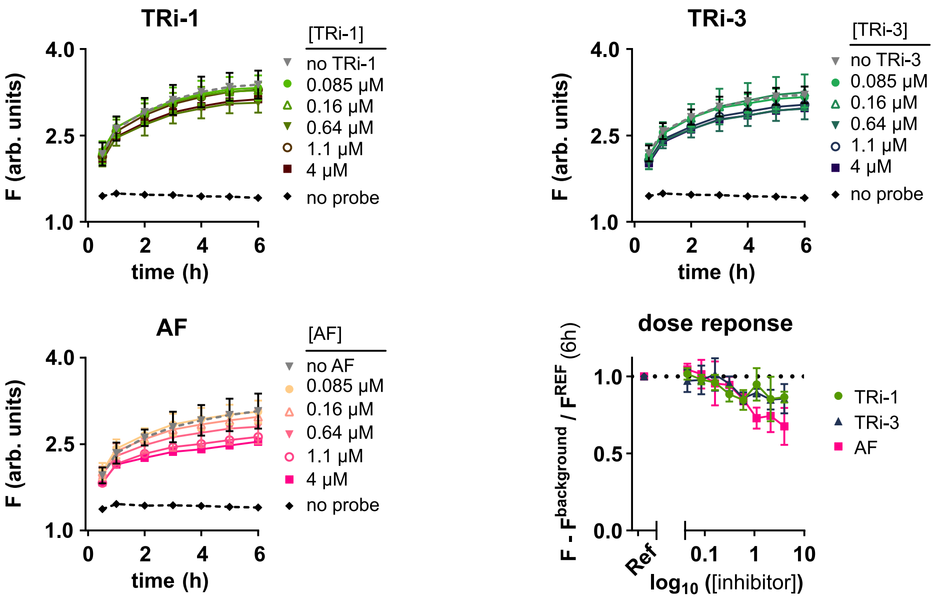

b **SS50-PQ** (25 µM) **- inhibitor studies - HeLa cells**

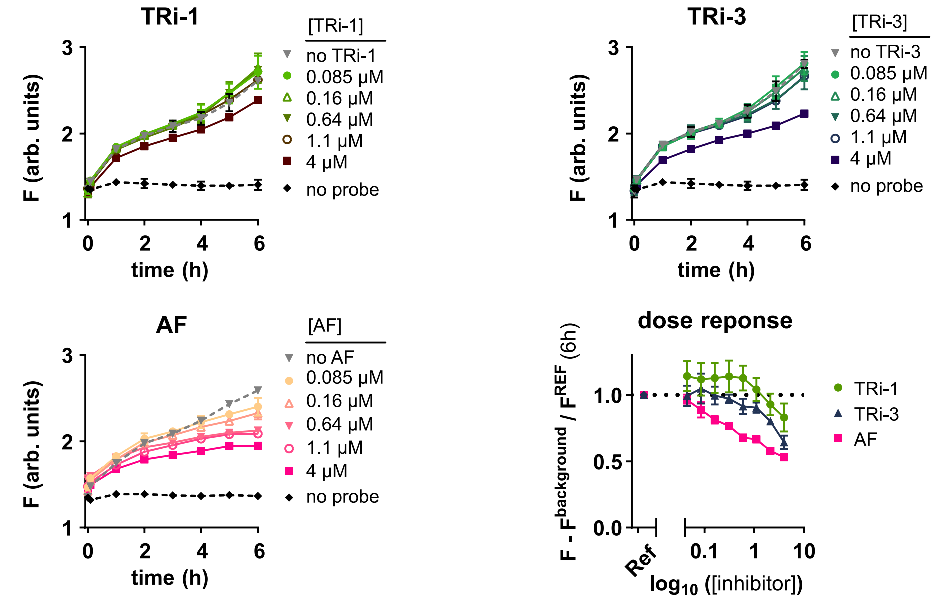

Supplementary Fig 10 Preliminary evaluation of cellular response of **SS50-PQ** under cellular treatment with known Au(I)-free TrxR inhibitors TRi-1 and TRi-3, and with the Au(I)-based inhibitor auranofin (AF). Conditions differ slightly from those used in the final evaluation (**Supplementary Figure 10**). Cellular fluorescence (ex/em 355bp10/520lp) and dose-response plots for (**a**) A549 cells pre-treated with TRi-1, TRi-3 and AF (25 µM **SS50-PQ,** 3 h pre-treatment). (**b**) HeLa cells were pre-treated (3 h pre-treatment) TRi-1, TRi-3 and AF and then treated with 25 µM **SS50-PQ**. *(**a-b**) show graphs representing Mean+SD calculated from 3 independent experiments.*

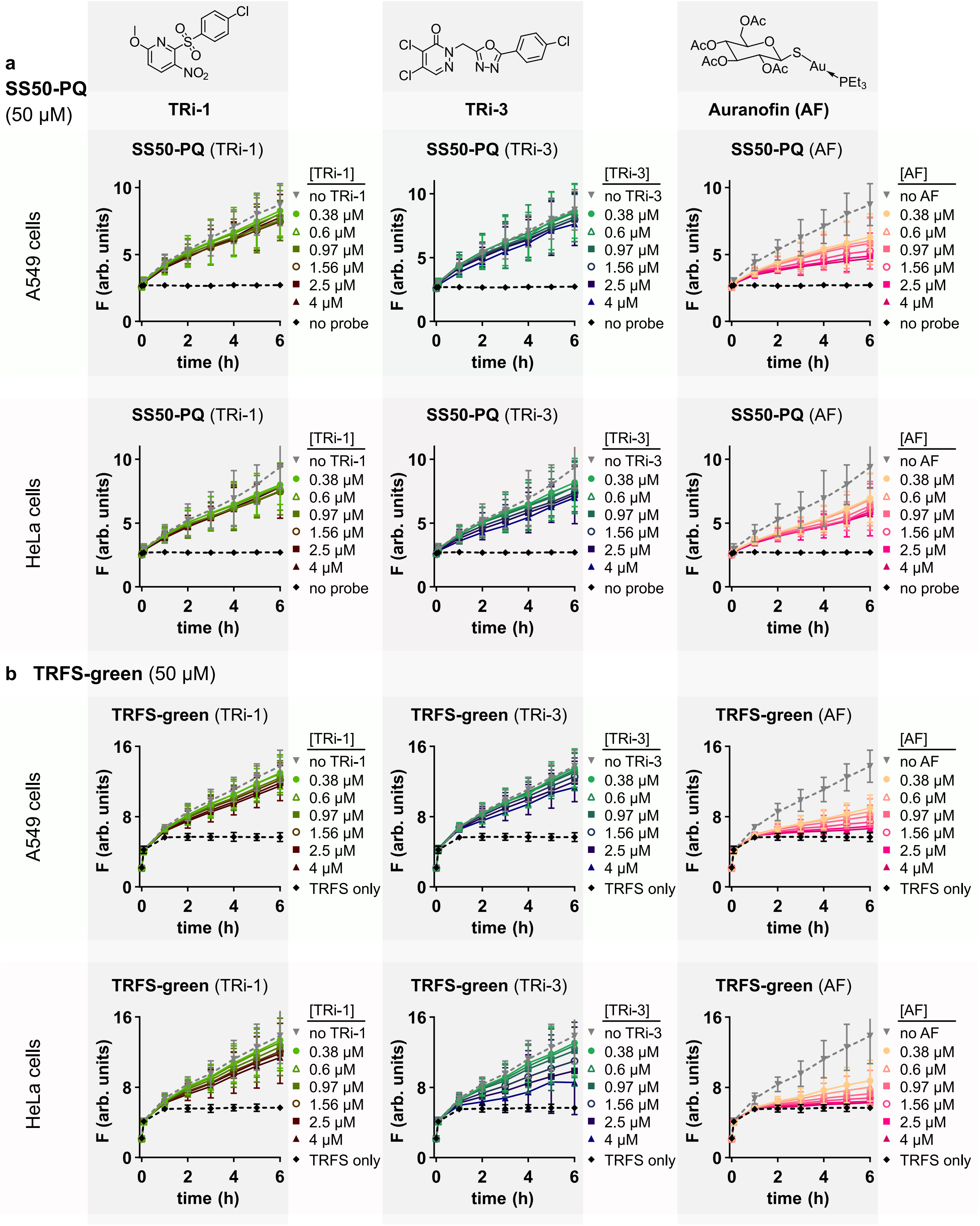

Supplementary Fig 11 Final comparison of **SS50-PQ** and **TRFS-green** cross-evaluated with TRi-1, TRi-3 and AF. Fluorescence timecourse upon cellular processing in (**a**) A549 cells pre-treated with TRi-1, TRi-3 and AF (50 µM **SS50-PQ** or **TRFS-green,** 2 h pre-treatment) and (**b**) HeLa cells pre-treated with TRi-1, TRi-3 and AF (50 µM **SS50-PQ** or **TRFS-green,** 2 h pre-treatment). Note that micromolar concentrations of these electrophiles are also cytotoxic.[24] *(**a-b**) show graphs representing Mean+SD calculated from 3 independent experiments.*

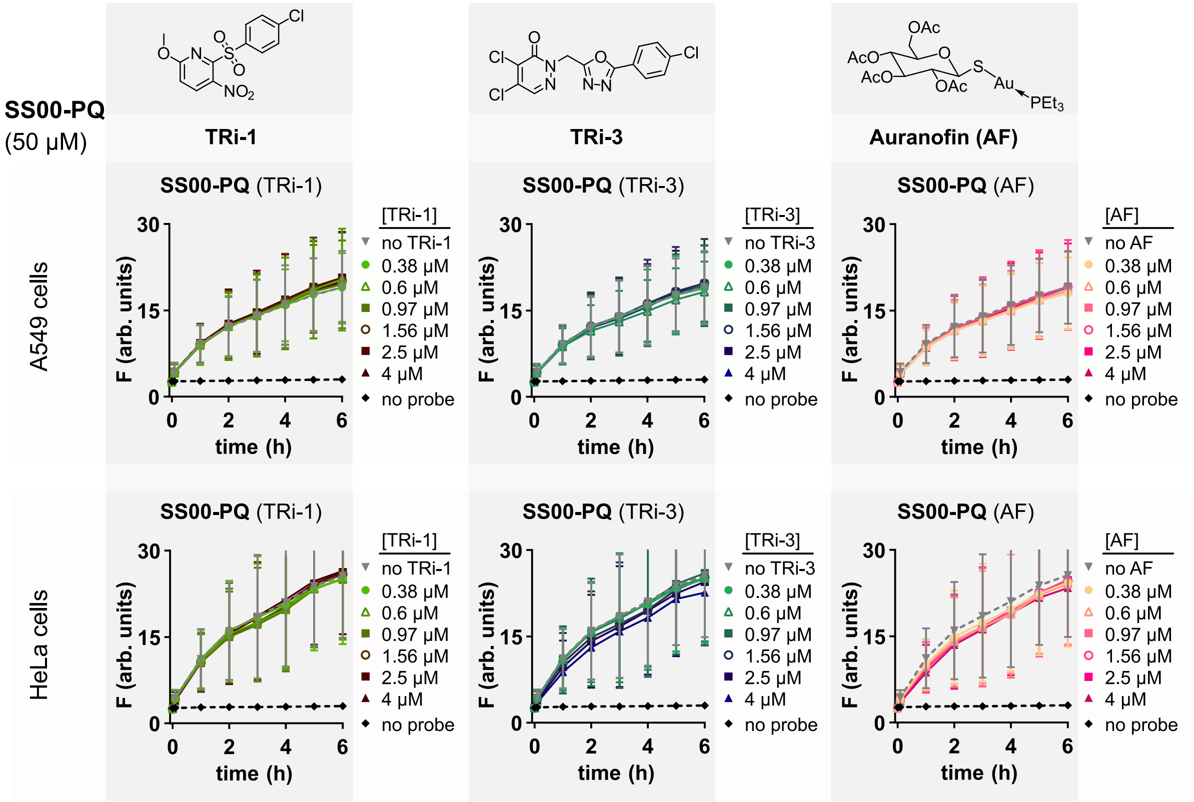

Supplementary Fig 12 Results of reference compound **SS00-PQ** cross-evaluated with TRi-1, TRi-3 and AF. Cellular fluorescence time-courses for A549 cells pre-treated with TRi-1, TRi-3 and AF (50 µM **SS00-PQ,** 2 h pre-treatment). *Graphs represent Mean+SD calculated from 3 independent experiments.*

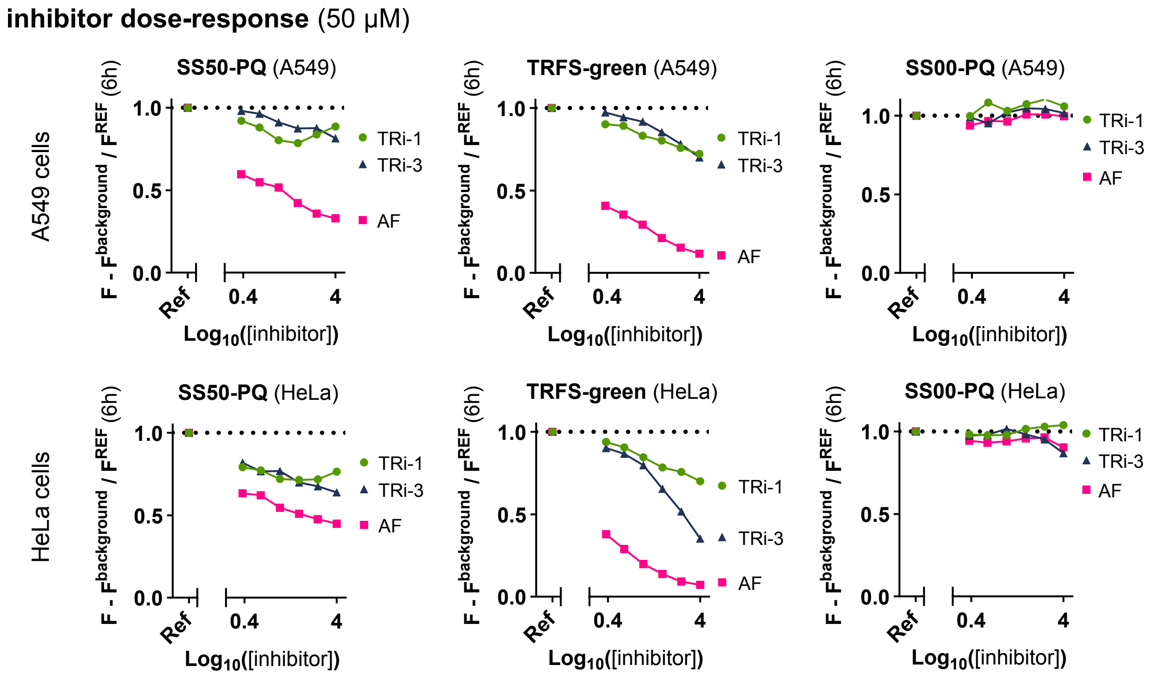

Supplementary Fig 13 Dose-response plots for **SS50-PQ**, **TRFS-green** and **SS00-PQ** cross-evaluated with TRi-1, TRi-3 and AF as specified above (timepoint t = 6 h). *Graphs show the data also presented in **Supplementary Figure 11-12** representing Mean calculated from 3 independent experiments.*

a **reference cells - gating**

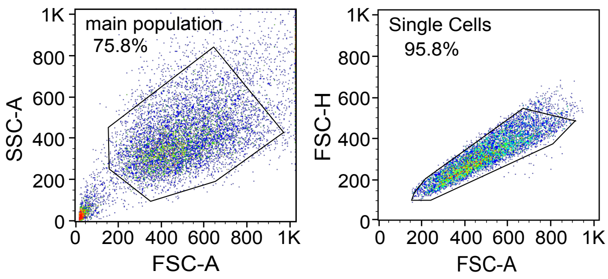

b **gating for PQ(+) cells of zombie(-) cell population**

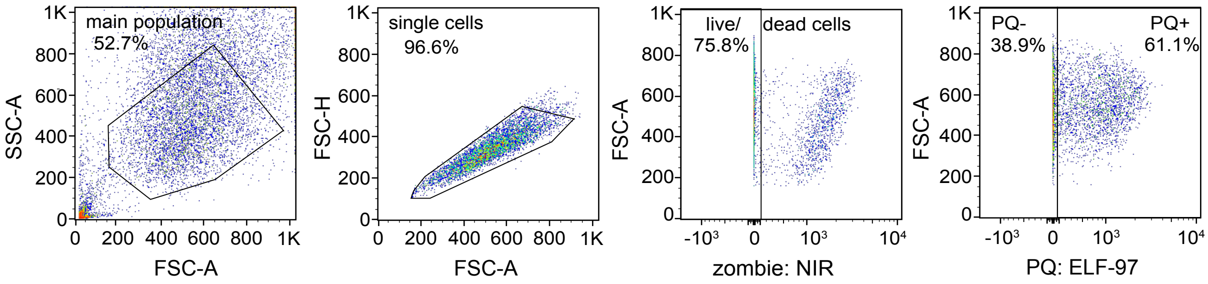

c

**total count**	**main population**	**single cells**	**zombie(-) stain**	**PQ(+) fluorescence**
**no inhibitor**	**5732** (57%)	**5569** (97%)	**5374** (97%)	**2524** (47%)
**TRi-1**	**5255** (53%)	**5041** (96%)	**4743** (94%)	**2729** (58%)
**TRi-3**	**5750** (58%)	**5561** (97%)	**5342** (96%)	**2843** (53%)
**AF**	**4341** (43%)	**3960** (91%)	**3615** (91%)	**3011** (83%)

d

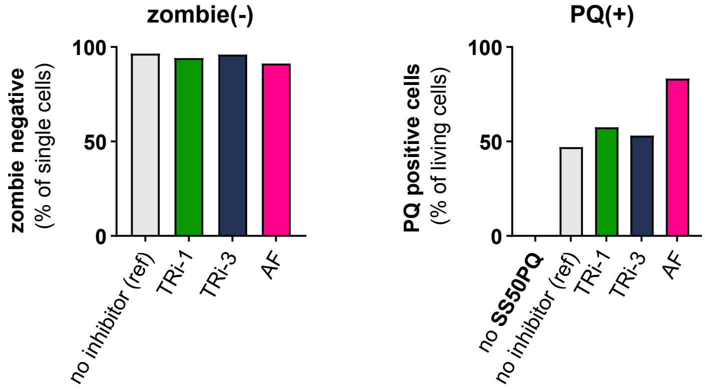

Supplementary Fig 14 Flow cytometry-based workflow for data processing, used to collect single cell statistics of Jurkat T-cells treated with **SS50-PQ**. (**a**) Untreated reference cells: (1) exclusion of debris in FSC-A/SSC-A, (2) selection of single cells in FSC-A/FSC-H. (**b**) Jurkat T-cells treated only with **SS50-PQ** (25 µM) gated according to (1) and (2), followed by (3) live/dead selection applying zombie-staining (ex/em 647laser/780bp60), (4) PQ(+) cells out of the zombie(-) cell populations by detecting ELF-97 fluorescence (ex/em: 365laser/520lp). (**c**) Flow cytometry-related total count for an example inhibitor screening experiment: Jurkat T-cells treated with **SS50-PQ** (25 µM) optionally after 3 h of pre-incubation with TRi-1, TRi-3 or AF (2 µM). (**d**) Results from (**c**) in bar chart representation: plot of live cells (Zombie-negative) as a percentage of all gated single cells; and PQ(+) live cells as a percentage of all live cells. *(**c-d**) show representative examples out of >3 independent experiments.*

a electrophilic inhibitor concentration screening (3 h pre-incubation)

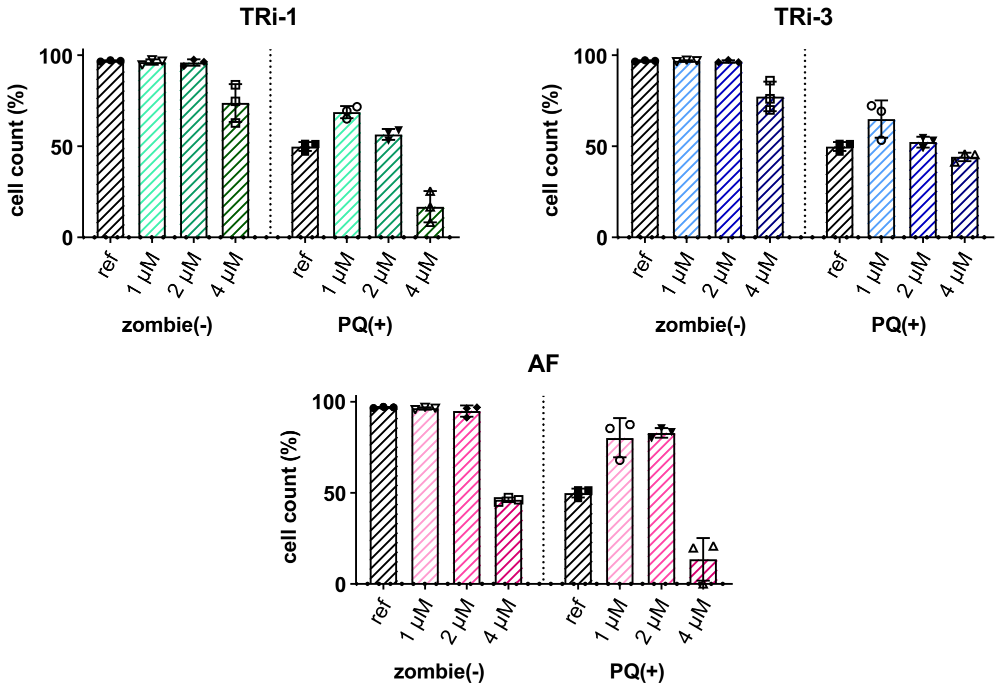

b electrophilic inhibitor comparison

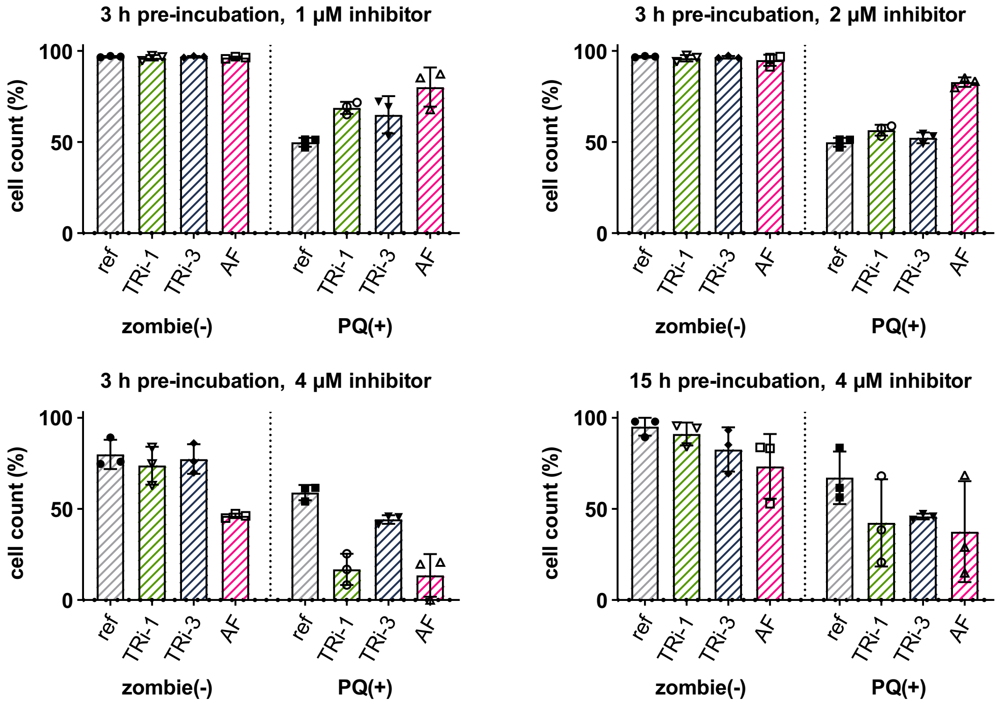

Supplementary Fig 15 (**a**) Flow cytometry-based single-cell statistics of Jurkat T-cells treated with **SS50-PQ** (25 µM) optionally after 3 h of pre-incubation with TRi-1, TRi-3 or AF (1-4 µM) to determine the onset of toxicity with each inhibitor. Comparison of live cells (Zombie-negative) as a percentage of all gated single cells; and PQ(+) live cells as a percentage of all live cells. (**b**) Results with TRi-1, TRi-3 or AF used at nontoxic concentrations (1 µM and 2 µM for all inhibitors) and at moderately toxic concentration (4 µM), with pre-incubations of cells with inhibitor either for 3 h (all concentrations) or 15 h (only 4 µM). *Graphs show barchart representation using Mean+SD calculated from 3 independent experiments including individual datapoints for each experiment.*

1.8 Supplementary Note 2: Auranofin's polypharmacology

Our cell-free and cellular assays had shown that 1,2-dithiolane is not selective for TrxR. However, previous studies of 1,2-dithiolane-based probes have claimed excellent TrxR selectivity. This claim was substantially based on assays where exposure of cells or lysates to the chalcophilic Au(I) complex auranofin (AF) dose-dependently reduced fluorescence signals as compared to untreated controls.[9] We were intrigued to study AF more closely, to understand if/why it may have led to misinterpretation of selectivity for 1,2-dithiolane-based probes.

AF is a chalcophilic Au(I) species that is popularly used as an inhibitor of TrxR. AF does bind TrxR in cell-free assays and in cells. However, AF is a broadly "potent thiol-reactive species"[25] that is **also** reported to bind at least 20 other thiol protein targets. Its therapeutic mechanism/s of action are widely accepted to be unresolved.[26] Although it is clear that TrxR is one of its major targets,[26] it is also noted that AF is a particularly strong binder of membrane thiols, likely driven by its lipophilicity.[27]

We confirmed that cells pre-treated with AF (0.1 to 4 µM) had decreased probe processing, seen by both single-cell (**Supplementary Figure 14-15**) and population average (**Supplementary Figure 10-13**) measurements. However, this *does not* indicate that probe processing occurs through TrxR. Given AF's polypharmacology, the dithiolane's cell-free nonselectivity, and the minor effects of non-Au-based TrxR modulation on probe activation (TRi results in **Supplementary Figure 11 and 13**), we were motivated to look more critically into this result. Conclusively, even in a fully cell- and enzyme-free setting, AF catalytically suppressed the fluorescence generated upon TCEP treatment, without a clear dose-dependence (**Supplementary Figure 9**). Therefore, signal suppression in the cellular assay cannot be used to argue that TrxR, or any other cellular target of auranofin, might be a selective reductant of 1,2-dithiolane.

We present two hypotheses that plausibly explain why AF suppresses signal from **SS50-PQ**, that are consistent with 1,2-dithiolane being a nonspecific thiol probe for which TrxR is not a significant cellular reductant. (1) The strained disulfide probe usually benefits from dynamic covalent exchange enhancement of cellular delivery, therefore AF's binding to membrane thiols[27] suppresses cellular delivery and signal. Similar suppression of delivery of strained disulfide substrates has been achieved by thiophilic organic alkylators of exofacial thiols.[4] This delivery inhibition is consistent with literature findings and our data showing that 1,2-dithiolanes are nonspecificially opened by thiols (**Fig 3**), and it could be responsible for some or most of the cellular signal suppression by auranofin. (2) AF also reacts both directly and catalytically with 1,2-dithiolane-based probes to suppress signal by diminishing their reducibility. Literature reports indeed show that Au(I)-(thiolate)(phosphine) complexes react with disulfides, initially forming triethylphosphine oxide and the thioglucose-gold-thiolate complex.[28,29] Since Au(I)-bis(thiolate) complexes undergo facile net ligand exchange with disulfides, they mediate thiol-disulfide exchange reactions[30], which in the case of 1,2-dithiolane triggers ring-opening polymerisation,[31] which is discussed and demonstrated extensively for Fast-TRFS (**Figure 6**, **Supplementary Note 4**, etc). Polymerisation of the 1,2-dithiolane-based **SS50-PQ** would generate hydrophobic polymeric products, which (as we observed and as literature suggests[32]) are not reducible by even such strong aqueous reductants as TCEP, thus suppressing fluorescent signal generation. This would explain auranofin's cell-free signal suppression (**Supplementary Figure 9**), and could even be sufficient to explain the cellular signal suppression, that we and others have observed.

Motivated by the known broad spectrum of auranofin's targets and effects, it would be beneficial to at least take design precautions (e.g. cell-free controls testing AF-probe interactions, or using TRi inhibitors for competition assays) to identify likely confounding factors before advancing targets of strained disulfide probes based on AF experiments. However, we think it would be best to avoid interpreting Au-based reagent studies with strained disulfides altogether, due to the high chance of any assay result being misinterpreted based on a populous literature of AF targets with no clear consensus. These may be valuable guides for analysis of cellular redox probe selectivities in the future.

Conclusion and Outlook for Auranofin: We have pooled literature references with our data to show that inhibition of cellular activation of probes based on 5-membered cyclic disulfides by treatment with auranofin can result from at least two plausible, precedented processes that do not involve TrxR: i.e., delivery inhibition by binding exofacial thiols, and suppressing probe bioavailability and reducibility by catalysing dithiolane ring-opening polymerisation in extracellular medium. This calls generally for caution when claiming cellular mechanisms of probe activity based on inhibitor studies with the potential for direct inhibitor-probe interactions, spontaneous probe degradation, and known non-specific probe-activating mechanisms (such as 1,2-dithiolane exofacial thiolysis). In general, most biological literature using auranofin interprets AF assays in light of TrxR inhibition. Here, we have drawn attention to the thiol/disulfide reactivities of AF to caution that it cannot be used for mechanistic evaluation of cellular targets of strained disulfides, whereas recent gold-free TrxR inhibitors[1] may be better suited for this purpose. With a nuanced view of AF, a more promising avenue of research is suggested. If the FDA-approved auranofin shows substantial inhibition of disulfide-mediated uptake not only in monolayer cell culture but also in 3D models and *in vivo*, this could make it interesting for repurposing, or as a therapeutic lead. The cellular uptake of some viruses and toxins is mediated by thiols, and inhibitors of this process are valuable targets, with the most potent inhibitors reported[33] apparently being comparable to what we have observed with auranofin. In an illustrative recent example, AF was reported to slow SARS-COV-2 replication by 95%, although inhibition of thiol-mediated uptake was not considered as a potential basis for this inhibition[34].

Separately, the interesting question has been raised in review, of whether a probe is likely to enter the cell, get reduced to the (di)thiol form, and only then coordinate with AF (as a monothiol, since the Au(I) prefers a linear coordination geometry that a 6-membered ring cannot permit) so reducing its cyclisation - which could be an alternative mechanism for cellular signal suppression. We do not think this is relevant. We consider that the intracellular background thiol concentration (5 mM GSH, up to 50 mM protein thiols) is much higher than the instantaneous reduced intracellular probe reduction (di)thiol intermediate is ever likely to be, particularly for the rapidly-cyclising **SS50-PQ**), and since we see no basis for selective AF reaction with the reduced (di)thiol intermediate, we do not imagine that intracellular reaction can be significant for suppression of signal generation - particularly since AF is applied at max. 4 μM and typically we are using probe at up to 50-100 μM.

1.9 Lipid vesicle-mediated ROP of Fast-TRFS

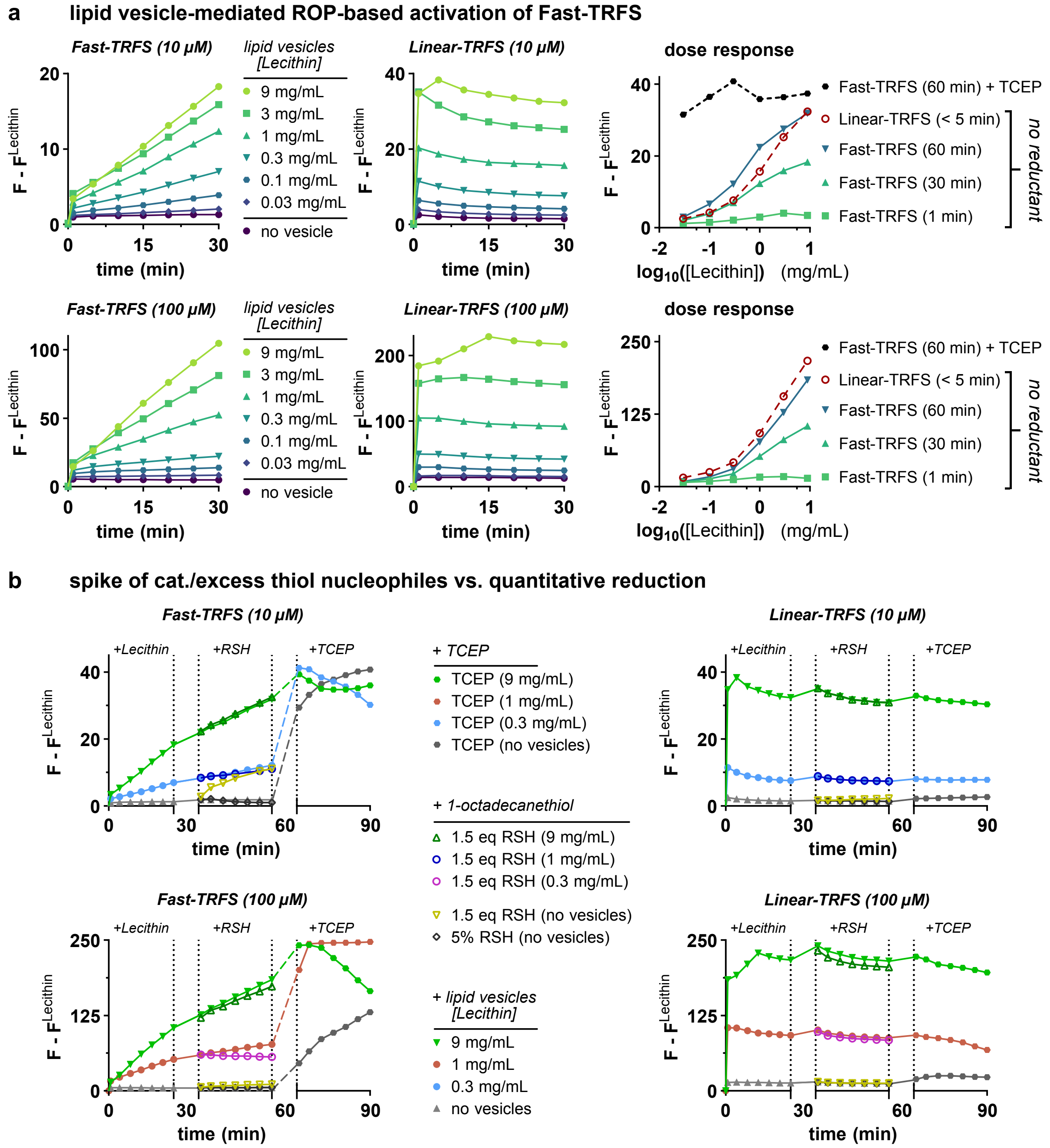

Supplementary Fig 16 (**a**) Fluorescence timecourse (355bp10/460bp20) monitored for **Fast-TRFS** and **Linear-TRFS** (both at 10 µM and 100 µM as indicated) incubated at 37°C with solubilised lipid vesicles (prepared from commercial lecithin at 0.03-9 mg/mL in deionised water according to Fromherz *et al.*[35] Background fluorescent signal (concentration-dependent scatter from lecithin) has been subtracted. (**b**) After 30 min of fluorescence monitoring, 1-octadecanethiol (RSH) was spiked at catalytic amounts (0.05 eq.) or in excess (1.5 eq. vs. probe concentration) and compared to an untreated reference experiment. After 30 min of further fluorescence monitoring, the potential for additional, *reduction-mediated* fluorescence increase was tested by treatment with excess TCEP (at 100 µM) and fluorescence was further monitored for 30 min. *(**a-b**) show graphs representing single experiments with technical replicates.* (1) The **Linear-TRFS** model compound's fluorescence turn-on *instantly reaching plateaus that depend on lecithin concentration* illustrates the environment-dependence of fluorescence of the intact aminocoumarin-urea disulfide; (2) **Fast-TRFS** turn-on is, without reduction required, concentration-dependently initiated in the presence of a lipid environment resulting from surface-initiated strain-promoted ring-opening polymerisation of the vesicle-partitioned proportion of probe.

1.10 Supplementary Note 3: Fast-TRFS' *TrxR-independent* fluorogenicity

Cell-free experiments with the known **non-cargo-releasing** 1,2-dithiolane-based probe **Fast-TRFS**[12] could show important conclusions, through comparison to results for its novel linear disulfide analogue **Linear-TRFS** we report here.

Fast-TRFS was intended to be fluorescence-quenched in its intact cyclic 1,2-dithiolane state, and was reported to be "a specific and superfast fluorogenic probe of mammalian thioredoxin reductase" that should act in cells by increasing in fluorescence upon "reduction [to **Dithiol-TRFS**, see **Fig 6**]"[12] We now show that there are at least three hitherto unreported confounding aspects to this **Fast-TRFS** probe system, and discuss how these combine with each other to give misleading results: and in doing so we support our thesis that 1,2-dithiolane is not a viable redox sensor for selective probes.

Firstly, in our hands, **Fast-TRFS** performs with poor reproducibility that matches entirely to Whitesides' characterisation of polymerisation-prone dithiolanes,[36] and to the observations we report about polymerisation-prone **SS50-PQ** and its precursors (see main text).

Secondly, we show that a ring-opened species resulting from strain-promoted thiol attack on the **Fast-TRFS** 1,2-dithiolane [model compound **Linear-TRFS**] will be *just as fluorescent, as the fully-reduced* **Dithiol-TRFS** (e.g. on the dose-response panel in **Fig 6d**, at 9 mg/mL lecithin, ca. 36 fluorescence units). This means that any of several likely TrxR-independent ring-opening mechanisms will generate TrxR-independent fluorescence signal from **Fast-TRFS** (**Fig 6**, **Supplementary Figure 16**). These mechanisms include but are not limited to: (a) strain-promoted oligomerisation, that should be particularly relevant if high local concentrations of **Fast-TRFS** are created, resulting in all ring-opened probe molecules becoming fluorescent (see below and shown in **Fig 6c**); (b) strain-promoted cellular uptake is via the known mechanism of exofacial thiol attack on the dithiolane and so will likewise activate fluorescence before it ever reaches the intracellular environment; (c) opening of the dithiolane by any cellular thiol.

Thirdly, we show that the fluorescence signal from **Fast-TRFS** and from its oligomerisation and its reduction products, also have strong environment dependence which creates additional problems. For example, oxidised **Fast-TRFS** fluorescence is instantly ca. 3-fold enhanced just by moving from aqueous to apolar environment (at the first minute of **Fig 6d**, the fluorescence intensity without vesicles is 0.99, but rises to ca. 3.4 as the lipid concentration increases - i.e. already 1/10 of the total potential fluorescence signal) - and this is before *non-reductive* strain-promoted ring-opening has significantly taken place (which it then does, reaching 50% of completion within the next 29 minutes). We also show that the environment-dependence of the Fast-TRFS system entirely rules the fluorescence behaviour of the model compound **Linear-TRFS**, which is up to 17-fold enhanced just by exposure to lipids (**Fig 6d**: from 2.0 to 35) but is *almost entirely unaffected by reduction* (irrespective of which environment the probe is in - see TCEP spike at the end of its incubation, **Supplementary Figure 16**). Therefore, the faster that a **Fast-TRFS** probe of any redox status accumulates into (intracellular) lipid environments, the more quickly that the cellular fluorescence will rise.

The combination of these three effects provides a unified model rationalising the published "superfast" cellular fluorescence turn-on of **Fast-TRFS** without recourse to TrxR, which depending on the situation under testing may be relevant or dominant.

We propose that in cells, a local-concentration-dependent ring-opening-oligomerisation can be activated by partitioning the dithiolane **Fast-TRFS** from a relatively large volume of aqueous medium into a relatively smaller volume of apolar environment (ideally, of high surface area for rapidity), with subsequent need only for catalytic activation by a nucleophile that may or may not be a membrane-associated reductase. Then **Fast-TRFS** fluorescence (which already jumped threefold upon entering the apolar medium) would rapidly rise as increasing amounts of the more fluorescent oligomer would be formed. We predicted that this could allow even maximal theoretical fluorescence signal to be reached either with catalytic, or even zero, added reductants. We tested this experimentally with the series of vesicle assays (**Fig 6** and **Supplementary Figure 16)**, simply sonicating commercial soybean lecithin (Sigma, P5638, ca. 60% phospholipids and 35% oils; zero content of TrxR or NADPH) in water to form a lipid vesicle stock,[35,37] diluting it to various concentrations and mixing with **Fast-TRFS**. Surpassing our expectations, in just one hour, the **Fast-TRFS** signal in 0.9%wt vesicle mixture without any reductants reached *full maximal signal plateau, corresponding to that seen with full TCEP reduction to* **Dithiol-TRFS**. This can only be understood as local-concentration-dependent oligomerisation to the poly-(disulfido-TRFS), that is just as fluorescent as **Linear-TRFS** or **Dithiol-TRFS** when they are compared in the vesicle system (**Supplementary Figure 16**). This shows the confounding influence of redox-independent, strain-promoted processes on 1,2-dithiolane chemical behaviour: and illustrates how the environment-dependent readout of the **Fast-TRFS** compounds expands this complexity by generating a false positive signal.

1.11 Supplementary Note 4: TRFS-green's *TrxR-independent* fluorogenicity

*In the discussion following **Supplementary Figure 3**, we have argued based on rates of reaction with 10-fold excess of TCEP, that the very slow release-based signal generation of **TRFS-green** makes it to our understanding impossible that this probe can hope to report cleanly on TrxR activity in cells, instead of giving a signal that is entirely dominated by environment-dependent effects.*

As the aminonaphthilimide core of **TRFS-green** is also a classic environment-dependent fluorophore we looked in the literature to see if this might be a similarly confounding issue as environment effects were shown to be for **Fast-TRFS**. We note that recently published results from the Fang group[38] show that their naphthilimide-based compound S1, which is similar to **TRFS-green**, experiences an instantaneous 45-fold enhancement of fluorescence intensity upon leaving all-aqueous environment and noncovalently associating to albumin in a cell-free experiment (their paper's Figure 3; note too that their aminocoumarin compound S3, which is similar to **Fast-TRFS**, has a ca. 4-fold fluorescence enhancement in the same experiment, matching our vesicle results with **Fast-TRFS**).

Paralleling our experimental investigation of TrxR-independent signal generation in the **Fast-TRFS** system, this literature report of environment-dependent-signal highlights how interpreting fluorescence increases with the **TRFS-green** probe may similarly not be straightforward since (1) in any context, even just leaving the aqueous (extracellular) environment will trigger a fluorescence increase, that can be entirely independent of any reaction on its 1,2-dithiolane motif; (2) in the cellular context, all TrxR-independent strain-promoted thiol-mediated uptake at the cell surface will covalently associate the **TRFS-green** probe onto membrane proteins and into membranes, thereby giving cell-uptake-driven signal independent of molecular encounter of the **TRFS-green** probe with intracellular TrxR, let alone cyclisation-driven release of the cargo. It is therefore consistent with these hypotheses that the same factors as we advanced for the dithiolane **Fast-TRFS** (concentration into membranes aided by membrane-thiol-based opening of the strained dithiolane, which gives an environment-dependent signal turn-on) will apply to **TRFS-green**, permitting it to generate signal based on cellular exofacial thiol status, without even encountering TrxR.

These suggestions could particularly be considered, in light of the demonstrations that cellular signal from **TRFS-green** is manifestly independent of TrxR and saturates at low concentrations (see legend to **Supplementary Figure 8**).

1.12 *In vivo* animal model, zebrafish: results and impact

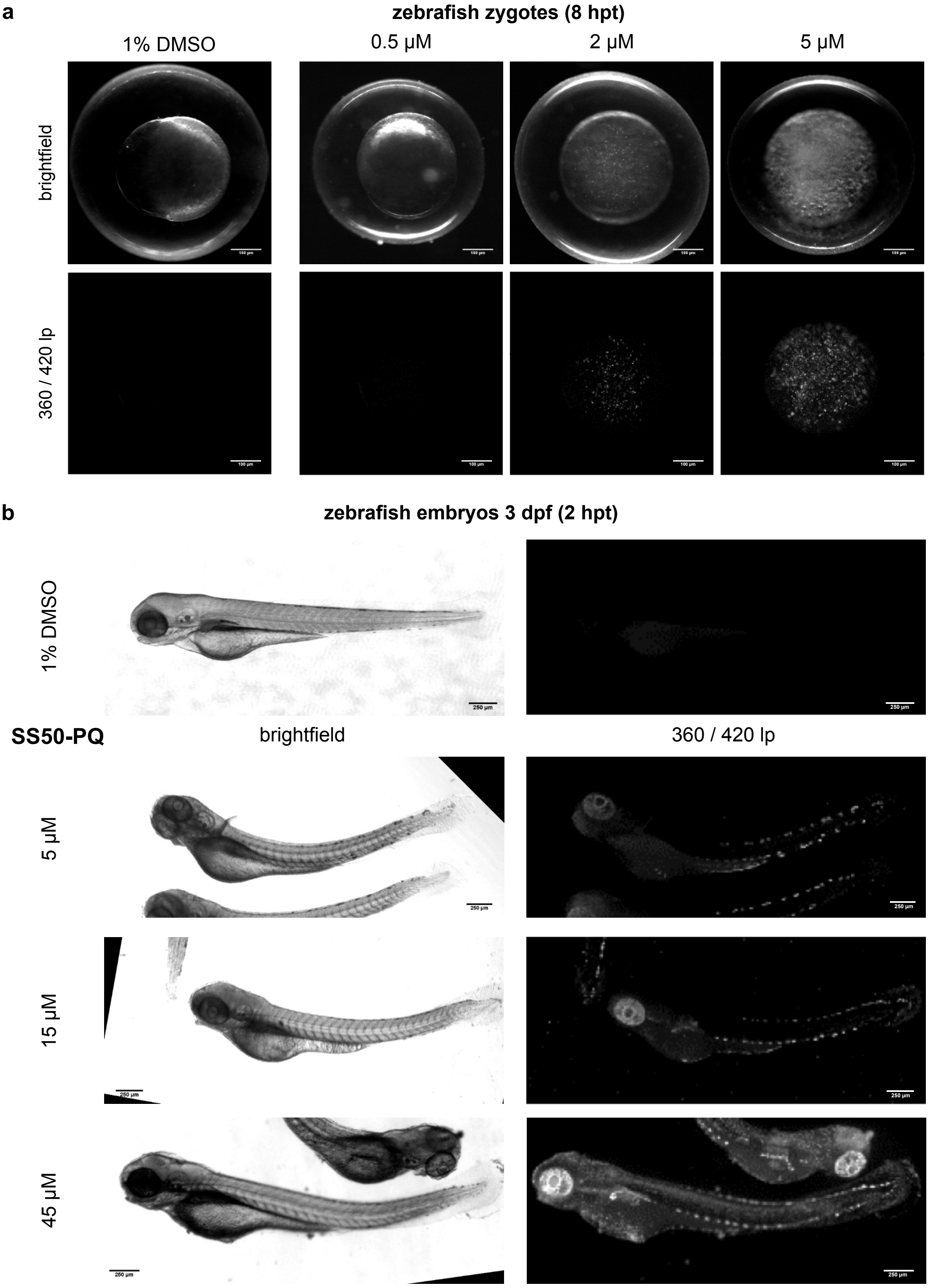

Supplementary Fig 17 *in vivo* animal model for reduction-responsive imaging. Representative epifluorescence stereomicroscopy images of (**a**) zebrafish zygotes incubated **SS50-PQ** (8 hpt) (representative image of 3 independent experiments with similar results); (**b**) zebrafish embryos (3 dpf) treated with **SS50-PQ** (2 hpt) (representative image of 3 independent experiments with similar results).

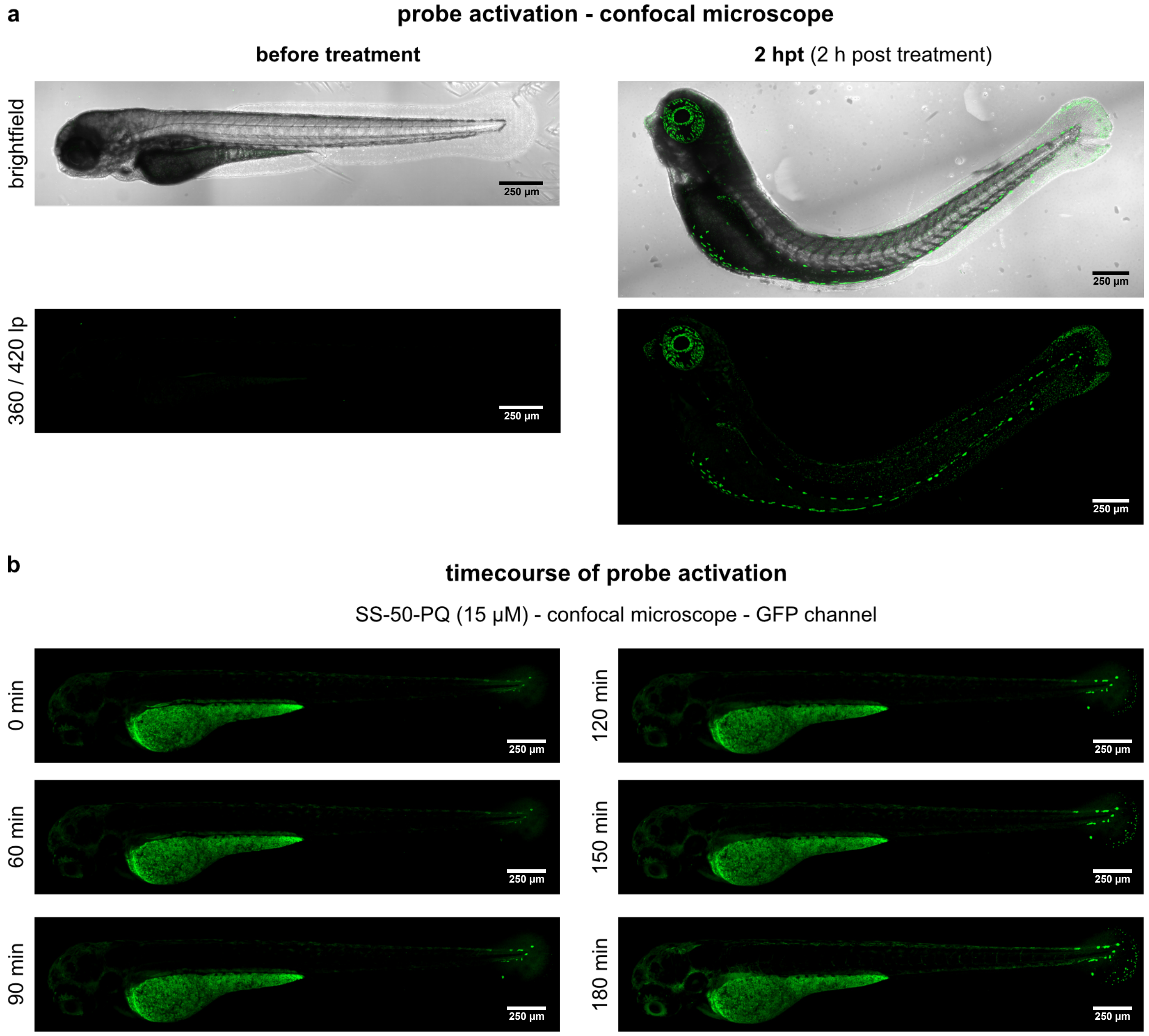

Supplementary Fig 18 (**a**) Overlay of representative transmission microscope and confocal fluorescence microscope images of zebrafish embryos (3 dpf) incubated with **SS50-PQ** (15 µM) before treatment and 2 hpt (representative image of 3 independent experiments with similar results). (**b**) Timecourse fluorescence increase recorded using a confocal microscope (ex/em 405 nm/520bp20 "GFP" filter) at 0 min, 60 min, 90 min, 120 min, 150 min and 180 min after treatment. Fluorescence channel images (green overlay) are maximum projections (representative image of 2 independent experiments with similar results).

Discussion of *in vivo* imaging in zebrafish

Activation of fluorescence, as compared to untreated control experiments, was already observed in zebrafish embryos at low **SS50-PQ** concentrations (5 µM). Aggregation of solid, fluorescent particles with high brightness inside the living cells was observed. Connected to the release of solid particles, death was subsequently observed.

When embryos already at a mobile development stage (3 dpf) were incubated with **SS50-PQ**, signal was observed after short treatment time (2 hpt) at very low concentration. A clear response of the animals to the formation of the solid was observed leading to deformation, limitation of mobility and death. At 8 hpt no live animals were observed. Maximum projection images were taken for the off-to-on signal switch when zebrafish embryos (3 dpf) were incubated with **SS50-PQ** (15 µM) and solid particles could be resolved on a single-cell level and localized due to transparency of the organism. Release of solid, fluorescent particles was observed time-dependently after incubation with **SS50-PQ**. Presumably, due to aggregation of release solid, the particles itself grow over time. Note that time-resolved confocal microscopy was possible because embryos were immobilized by embedding in gel. The PQ probe system design is responsible for the irreversible and non-diffusive accumulation of the bright, photostable signal inside cells, allowing for cellularly-resolved imaging, with the high Stokes shift delivering excellent signal to noise ratios, which are beneficial for further use with other trigger systems.

1.13 Supplementary Movie Caption

Supplementary Movie 1: Representative confocal time lapse microscopy of live HeLa cells treated with **SS50-PQ** (50 µM) and immediately imaged for one hour at ex/em: 405/530bp20; fluorescence channel (green) overlaid on brightfield transmission image.

2 Supplementary methodsMethods for *in vitro* evaluation

2.1.1 Cell-free chemoreductant assays

Cell-free *in vitro* assays were performed to evaluate the activation of **SS50-PQ** and **TRFS-green** opon reduction-mediated release of the water-insoluble dye **PQ-OH**$_{(s)}$ (precipitation-based induction of ESIPT-based fluorescence monitored with ex/em 355bp10/520lp) or of 1-butyl-6-amino-naphtalimide (fluorescence monitored with ex/em 445bp10/520lp). The molecular process was monitored by fluorescence intensity increase based on the off-to-on mechanism. For **TRFS-green** subtraction of background fluorescence was conducted as indicated. We noted that upon standing **SS50-PQ** probe stocks had reduced capacity to generate fluorescence in reductant assays. Consequently, probe of **SS50-PQ** stocks were prepared freshly from the solids for all assays.

In a black 96-well plate with black bottom, 80 µL of a diluted solution (12.5 µM in aq. TE, pH 7.4, 1.25% DMSO) of **SS50-PQ** (final concentration 10 µM) was mixed with a solution of selected chemical reductants (20 µL of a solution (50 µM to 50 mM in aq. TE, pH 7.4). As reductants were used tris(carboxyethyl)phosphine (TCEP) at 100 µM final concentration; dithiothreitol (reduced, DTT), glutathione (reduced, GSH), *L*-glutathione (oxidized, GSSG), *L*-Cysteine (Cys), *L*-Serine (Ser), mercaptoethyl-dimethylamine (MEDA), cysteamine (CA) and *N*-acetyl *L*-Cysteine (NAC) all at 1 mM final concentration. For cell-free inhibition experiments 80 µL of a diluted solution (62.5 µM in aq. TE, pH 7.4, 1.25% DMSO) of **SS50-PQ** (final concentration 25 µM or 50 µM as indicated) was pre-treated with 2-((4-chlorophenyl)sulfonyl)-6-methoxy-3-nitropyridine (TRi-1), 4,5-dichloro-2-((5-(4-chlorophenyl)-1,3,4-oxadiazol-2-yl)methyl)pyridazine-3(2H)-one (TRi-3)[1] (from DMSO stock solutions) or auranofin (AF) (from ethanolic stock solutions) with final concentrations from 0.1 µM to 4.5 µM 2 h before maximum reduction was induced by addition of 20 µL TCEP (at 100 µM final concentration).

The reaction mixtures were incubated at 37 °C and 100% humidity for 6 h. Time-course measurements were conducted to determine kinetics of the reduction-mediated release. Data was interpreted by calculating the absolute, time-dependent fluorescence intensity **F(t)** normalised to the plateau signal observed in a reference maximum-fluorescence experiment $\mathbf{F^{TCEP}(t)}$ that used incubation with 100 µM of TCEP. For monothiol-reductant concentration-dependent **SS50-PQ** was challenged with increased concentrations of glutathione (reduced, GSH) at 10 µM, 30 µM, 100 µM, 300 µM, 1 mM, 3 mM and 10 mM and the dose-response relation was later calculated from data representation on a $\mathbf{log_{10}}$[GSH/probe] vs. $\mathbf{F/F^{TCEP}(t = 6h)}$ graph. Dose-reponse curves were fitted using GraphPad Prism version 8.0.2. for windows from GraphPad Software, San Diego (USA).

2.1.2 Cell-free bioreductant assays

Cell-free in vitro enzyme specificity assays (bioreductants) were performed to evaluate the specificity and reaction kinetics of **SS50-PQ** and **TRFS-green** to enzymatic reduction based on the reduction-mediated release of **PQ-OH**$_{(s)}$ or of 1-butyl-6-amino-naphtalimide after incubation with bioreductant cascade model systems. The molecular process was equally monitored by fluorescence intensity increase (ex/em 355bp10/520lp or 440bp10/520lp). In a black 96-Well Plate with black bottom, 50 µL of a diluted solution (20 µM in aq. TE, pH 7.4, 2% DMSO) of **SS50-PQ** or **TRFS-green** (final concentration 10 µM) was mixed with 40 µL of the selected oxidoreductase enzyme (TrxR1, TrxR2, GR to reach final concentrations of 20 nM) and/or its respective native substrate (Trx1, Trx2, TRP14, Grx1, Grx2 to reach final concentrations of 10 nM to 10 µM respectively), including 100 µM final concentration of GSH in case of GR-Grx-GSH system. The biochemical reaction was started by addition of 10 µL of a solution of *β*-NADPH (1 mM in aq. TE, pH 7.4 to reach 100 µM final concentration). The reaction mixtures were incubated at 37 °C and 100% humidity for 6 h.

Human recombinant thioredoxin (Trx1 and Trx2) (lyophilized) human recombinant glutaredoxin (Grx1 and Grx2) (lyophilized from 10 µL TE-buffer, pH 7.5), human recombinant thioredoxin-related protein of 14 kDa (TRP14), human thioredoxin reductase (TrxR1 and TrxR2) (1.5 m/mL in 50% glycerol/TE-buffer, pH 7.5) and human recombinant glutathione reductase (GR) (100 µM in 50% glycerol/TE-buffer, pH 7.5) were obtained from *IMCO Corp.*, Stockholm (Sweden) or produced and purified as previously described.[39–42]

The reaction mixtures were incubated at 37 °C and 100% humidity for 6 h. Time course measurements were conducted to determine kinetics of the enzyme-mediated release. Data was interpreted by calculating the *absolute, time-dependent* fluorescence intensity **F(t)** corrected by the *absolute, time-dependent* background fluorescence $\mathbf{F^{NADPH}(t)}$ caused by autofluorescence of reduced *β*-NADPH normalised to the plateau signal observed in a reference maximum-fluorescence experiment $\mathbf{F^{max}(t)}$ with 100 µM of TCEP. Dose-reponse curves were fitted using GraphPad Prism version 8.0.2. for windows from GraphPad Software, San Diego (USA).

2.1.3 Cell-free environment-dependent polymerisation assay

Commercial *L*-α-lecithin (CAS 8002-43-5, derived from Sigma-Aldrich P5638) was hydrated with ultrapure water (Milli-Q, Reptile Bioscience Ltd., Boston, MA) to a final concentration of 9 mg/mL. The suspension was gently vortexed to achieve a homogeneous phase, which was then subjected to one freeze/thaw cycle. The stock solution was extruded through a polycarbonate membrane with a pore diameter of 400 nm, 21 times, using a Mini Extruder (Avanti Polar Lipids, Inc., Alabama, United States) according to Fromherz *et al.*[35] The stock was then diluted with deionised water to the indicated concentrations and treated with DMSO-stock solutions of **Fast-TRFS** and **Linear-TRFS** to final probe concentrations of 10 µM or 100 µM with maximum 1 % DMSO content. Fluorescence intensity (355bp10/460bp20) was recorded overtime and 1-octadecanethiol (RSH) was optionally spiked at catalytic amounts (0.05 eq.) or in excess (1.5 eq. vs. probe concentration) and compared to an untreated reference experiment. The potential for additional, *reduction-mediated* fluorescence increase was tested by treatment of the system with excess TCEP (0.1 mM, i.e. 1-10 eq.) and fluorescence was further monitored for 30 min.

2.2 Cell culture methods

2.2.1 General cell cultivation methods

HeLa, A549 or MEF cells were grown in Dulbecco's modified Eagle's medium (DMEM: L-glucose (4.5 g/L), *L*-glutamine, *L*-pyruvate, phenol-red, $NaHCO_3$ (2.7 g/L)) purchased from PAN Biotech, Aidenbach (Germany). Jurkat cells were grown in RPMI-1640 medium (*L*-glutamine, sodium bicarbonate) purchased from Sigma-Aldrich Life Science by Merck KGaA, Darmstadt (Germany). Media were supplemented with 10% heat-inactivated fetal bovine serum, penicillin (100 U/mL), streptomycin (100 µg/mL) and optionally with Na_2SeO_3 (0-100 nM) or Na_3PO_3S (0-1.2 mM). For washing and resuspending steps PBS Dulbecco buffer from Merck GmbH, Darmstadt (Germany) was used and for trypsination, TrypLE™ Express from gibco Life Technologies Inc., Massachusetts (USA) was used. Centrifugation steps were conducted using a centrifuge 5810R from Eppendorf GmbH, Hamburg (Germany) was used. Cells were grown at 37 °C under 5% CO_2 atmosphere and cell growth was confirmed using a Nikon Eclipse Ti microscope from Nikon Corp., Minato (Japan).

2.2.2 Cell lines

HeLa (DSMZ; ACC57), A549 (DSMZ; ACC107) and Jurkat (ATCC; TIB-152) cell lines were purchased form the German Collection of Microorganisms and Cell Cultures. TrxR1 knockout and reference mouse embryonic fibroblasts (MEF) are a kind gift from Marcus Conrad. Briefly, MEFs isolated from conditional TrxR1 knockout mouse embryos, were immortalised by lentiviral transduction. In vitro deletion of TrxR1 was achieved by Tat-Cre induced recombination and verified by PCR and Immunoblotting for TrxR1.[15] All cell lines are tested regularly for mycoplasma contamination and only mycoplasma negative cells are used in assays.

2.2.3 Cellular activation and inhibition assays

For evaluation of cellular processing of **SS50-PQ** and **TRFS-green** cells were seeded in 96-well plates (Microplates, 96-Well, F-Bottom, black, Fluotrack, high binding) from Greiner bio-one GmbH, Kremsmünster (Austria) at a medium level of 100 µL. Cell medium was treated with **SS50-PQ**, **TRFS-green** or respective reference compounds **SS00-PQ** or **RX1**(from stock solution in 100% DMSO) to reach final concentrations of 1 µM to 100 µM at maximum final levels of 1% DMSO. For inhibition experiments cells were pre-treated with the selected TrxR inhibitors TRi-1 and TRi-3[1] (from DMSO stock solutions) and AF (from ethanolic stock solution), 3 h or 15 h before probe treatment.

Treated cell plates were incubated at 37 °C under 5 % CO_2 atmosphere and timecourse fluorescence measurements were conducted to determine kinetics of cellular processing. Fluorescence readout of cell-free activity and/or cell assays was performed either using a *FluoStar Omega* plate reader from BMG Labtech, Ortenburg (Germany) (ex/em 355bp10/520lp or 440bp10/520lp) or a Tecan Infinite M200 plate reader from Tecan, Maennedorf (Switzerland) (ex/em 355bp10/520lp or 440bp10/520lp) recording fluorescence intensity. Data was interpreted by representing the absolute, time-dependent fluorescence intensity **F(t)** or representing time-dependent background-corrected fluorescence intensity $F(t)-F^{Background}(t)$ where indicated.

2.2.4 Fluorescence microscopy

Intracellular formation of solid green-fluorescent particles corresponding to a time-dependent increase **F(t)** was confirmed using a Nikon Eclipse Ti2 upright microscope from Nikon Instruments Europe BV, Amsterdam (Netherlands) (ex/em 355bp50/410lp; or transmitted light, as appropriate). Images were processed using Fiji version 1.51 (ImageJ)[43] open-source image processing software.

Confocal time lapse microscopy was performed on live HeLa cells seeded in 8-well ibiTreat µ ibidi slides from ibidi GmbH, Martinsried (Germany). Dishes were placed on the motorized stage of a Leica SP8 laser-scanning confocal microscope (Wetzlar, Germany), treated with **SS50-PQ** at 50 µM on stage and immediately imaged for one hour at ex/em: 405laser/530bp20 comparing the fluorescence and the brightfield image. Acquired images were processed using Fiji version 1.51 (ImageJ)[43] open-source software.

2.2.5 Flow cytometry-based single-cell statistics

For single-cell resolved statistical analysis of intracellular solid green-fluorescent particles resulted from reduction-mediated activation/release of **SS50-PQ** a flow cytometry-based method was developed and implemented.

After respective treatment with TrxR inhibitors and/or **SS50-PQ**, cells were harvested and stained with a fixable viability dye according to manufacturer's recommendations (zombie NIR™ Fixable Viability Kit, BioLegend). Cells were fixed in 4% paraformaldehyde (PFA) in PBS for 30 min and either stored in PBS or immediately resuspended in a wash buffer containing PBS with 1% bovine serum albumin (BSA) and 1 mM EDTA Disodium salt (0.1 mol/L or 0.2 N).

Flow cytometry was conducted at the BioMedical Centre Core Facility of the LMU Munich on a BD LSRFortessa instrument (BD Bioscience) using the BD FACS Diva Software. The following excitation/detection settings were used: zombie (ex/em 647laser/780bp60) and PQ fluorescence (ex/em: 355laser/530bp30). Data was processed using FlowJo v.10.7.1(BD).

An unstained sample was used to exclude cell debris and doublets. Zombie dye was used to exclude dead cells. PQ-positive gate was set, so that 0% of cells were PQ positive in an unstained sample. Cell debris, singlets, Zombie and PQ gates were set on an appropriate sample in each experiment and applied to all samples. General gating strategy is shown **Supplementary Figure 6**.

2.3 Methods for *in vivo* animal model

2.3.1 Animal model

Danio rerio (8 hpf or 3 dpf, sex undetermined) were housed in groups of 20-40 individuals in a fish facility (Aquaneering) maintaining approx. 700 mS, pH 6.9 – 7.1 and 28 °C with a 14/10 h light/dark cycle as outlined by common zebrafish handling guidelines.[44] All experiments used fertilized eggs from *ab* wild-type parents, grown in 30% Danieau medium (0.12 mM $MgSO_4$, 0.21 mM KCl, 0.18 mM $Ca(NO_3)_2$, 17.4 mM NaCl, 1.5 mM HEPES, pH 7.2) at 28 °C, again with a 14/10 h light/dark cycle. To suppress melanin pigmentation, 150 µM phenylthiourea (PTU) was added to the 30% Danieau medium at 8-10 hpf when needed.

All procedures involving animals were carried out according to EU guidelines and German legislation (EU Directive 2010_63, licence number AZ 325.1.53/56.1-TU-BS). As all zebrafish embryos in this study were analysed at ages below 5 dpf, hence no approval by an ethics board was required. The study adhered to ARRIVE guidelines.

2.3.2 *In vivo* activation experiments

SS50-PQ was prepared as stock solutions (10 mM in DMSO) and stored at 4 °C and briefly warmed to 37 °C before use. 5 µM, 15 µM and 45 µM dilutions were freshly prepared in 30% Danieau supplemented with DMSO to 1% (final concentration). At 8 hpf or 3 dpf, embryos were transferred to each well of a 6-well plate, and the 30% Danieau medium exchanged for the test dilutions (5 ml per well). Embryos were incubated at 28 °C until the formation of solid green-fluorescent particles was observed.

2.3.3 Imaging

Signal development was initially monitored on a stereofluorescence microscope (Leica M205FA or MDG41, from Leica Microsystems, Wetzlar, Germany) equipped with a UV filter (ex/em: 360bp40/420lp). Then for confocal imaging, the embryos were embedded in lateral position in 1.2% ultra-low gelling agarose (type IX-A), overlaid with the test solution. The embryos were transferred to a confocal microscope (Zeiss Airyscan from Carl Zeiss microscopy, Jena, Germany) equipped with a 405 nm diode and several laser lines, with a Life Imaging Services heating chamber set to 28 °C. Whole embryos were recorded using a 10× objective and the 405 nm diode for excitation, with subsequent tile/grid stitching using the Fiji version 1.51 (ImageJ) open-source software.[43,45] Image acquisition was controlled by the Zen Black software from Carl Zeiss microscopy, Jena, Germany.

2.4 Laboratory techniques

All solvents, reagents and building blocks were purchased from standard commercial sources. Anhydrous solvents obtained in septum-capped bottles and analytical grade or higher quality solvents were used without purification. Industrial grade solvents were distilled prior to use. Unless otherwise stated, reactions were performed at room temperature without precautions regarding air or moisture and were stirred using a magnetic Teflon®-coated stir bars. Air or moisture sensitive reactions were conducted in dry Schlenk glassware.

Flash column chromatography was performed on *Geduran® Si 60* silica gel from *Merck GmbH*, Darmstadt (Germany) an optionally conducted using a *Biotage® Select* automated column chromatography system from *Biotage GmbH*, Uppsala (Sweden). Unless stated otherwise, and thin layer chromatography to monitor reactions and determine R_f-values was performed on silica coated aluminium sheets with fluorescent indicator (*TLC Silica gel 60 F254* from *Merck GmbH*, Darmstadt, Germany) with visualisation by UV irradiation (254 nm/360 nm) or staining with $KMnO_4$ solution (3.0 g $KMnO_4$, 20 g K_2CO_3, 0.30 g KOH, 0.30 L H_2O).

2.4.1 Mass spectrometry

High resolution mass spectrometry (**HRMS**) was conducted either using a *Thermo Finnigan LTQ FT Ultra FourierTransform* ion cyclotron resonance spectrometer from *ThermoFisher Scientific GmbH*, Dreieich (Germany) applying electron spray ionisation (ESI) with a spray capillary voltage of 4 kV at temperature 250 °C with a method dependent range from 50 to 2000 u or a *Finnigan MAT 95* from *Thermo Fisher Scientific*, Dreieich (Germany) applying electron ionisation (EI) at a source temperature of 250 °C and an electron energy of 70 eV with a method dependent range from 40 to 1040 u. All reported *m/z* values refer to positive ionization mode, unless stated otherwise.

2.4.1 NMR spectroscopy

Nuclear magnetic resonance (**NMR**) spectroscopy was performed using a *Bruker Avance* (600/150 MHz, with *TCI cryoprobe*) *or a Bruker Avance III HD Biospin* (400/100 MHz, with BBFO cryoprobeTM) from Bruker Corp., Billerica (USA) either at 400 MHz or 500 MHz. ^{19}F-NMR spectra were recorded on a *Bruker Avance III* spectrometer (400 MHz for ^{1}H; 377 MHz for ^{19}F). NMR-spectra were measured at 298 K, unless stated otherwise, and were analysed with the program *MestreNova 12* developed by *MestreLab Ltd.*, Santiago de Compostela (Spain). ^{1}H-NMR spectra chemical shifts (δ) in parts per million (ppm) relative to tetramethylsilane (δ = 0 ppm) are reported using the residual protic solvent ($CHCl_3$ in $CDCl_3$: δ = 7.26 ppm, DMSO-d_5 in DMSO-d_6: δ = 2.50 ppm, CHD_2OD in CD_3OD: δ = 3.31 ppm) as an internal reference. For ^{13}C-NMR spectra, chemical shifts in ppm relative to tetramethylsilane (δ = 0 ppm) are reported using the central resonance of the solvent signal ($CDCl_3$: δ = 77.16 ppm, DMSO-d_6: δ = 39.52 ppm, CD_3OD: δ = 49.00 ppm) as an internal reference. For ^{1}H-NMR spectra in addition to the chemical shift the following data is reported in parenthesis: multiplicity, coupling constant(s), number of hydrogen atoms and, if available, assignment. The abbreviations for multiplicities and related descriptors are s = singlet, d = doublet, t = triplet, q = quartet, p = pentuplet or combinations thereof, m = multiplet and br = broad. The numbering scheme used for the assignments is specified in each case in a figure depicting the respective molecular structure and does not follow any convention. The reported assignments are supported by 2D-NMR experiments (HMBC, HSQC, COSY). Where known products matched literature analysis data, only selected data acquired are reported.

2.4.1 HPLC(-MS) analysis

Analytical high pressure liquid chromatography (**HPLC**) analysis was conducted either using an *Agilent 1100* system from *Agilent Technologies Corp.,* Santa Clara (USA) equipped with a DAD detector and a *Hypersil Gold* HPLC column from *ThermoFisher Scientific GmbH*, Dreieich (Germany) or a *Agilent 1200 SL* system *Agilent Technologies Corp.,* Santa Clara (USA) equipped with a DAD detector, a *Hypersil Gold* HPLC column from *ThermoFisher Scientific GmbH*, Dreieich (Germany) and consecutive low-resolution mass detection using a *HCT ultra PTM discovery* system applying ESI from *Bruker Corp*., Billerica (USA). For both systems mixtures of water (analytical grade, 0.1% formic acid) and MeCN (analytical grade, 0.1% formic acid) were used as eluent systems.

3 Synthetic Procedures

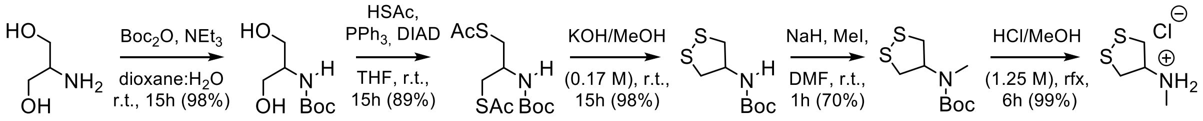

3 9 4 5 10 6

N-Boc serinol (9)[12]

2-amino-1,3-diol **3** (2.50 g, 27.4 mmol) was dissolved in a 1:1-mixture of dioxane:H_2O (200 mL, 0.13 M) and NEt_3 (4.85 mL, 35 mmol) was added. The mixture was cooled to 0 °C and a solution of Boc_2O (7.04 g, 32.2 mmol) dissolved in 1,4-dioxane (35 mL) was added, the reaction mixture was allowed to warm to r.t. and was then further stirred for 15 h.The solution was acidified to pH 4using 2 M aq. HCl, then saturated with potassium sodium tartrate and extracted with EtOAc (3×200 mL). The combined organic layers were dried over Na_2SO_4, filtered and concentrated under reduced pressure to give **9** (5.03 g, 26.3 mmol, 96%) as a colourless solid and used without further purification.[12]

TLC R_f = 0.50 (EtOAc). The product matched literature analysis data[12]. Selected data acquired: **HRMS** (EI): $C_8H_{15}NO_3^+$ [M-e]: calc. 173.1046, found 173.1046. **^{1}H-NMR** (400 MHz, $CDCl_3$): δ (ppm) = 5.29 (d, *J* = 6.7 Hz, 1H), 3.81 (dd, *J* = 11.1, 4.4 Hz, 2H), 3.74 (dd, *J* = 11.1, 4.5 Hz, 2H), 3.67 (s, 1H, N*H*), 2.89 (s, 2H, O*H*), 1.44 (s, 9H).

N-Boc 2-amino-1,3-bis(acetylthio)propane (4)[46]

Protocol A:

To a solution of PPh_3 (13.50 g, 51.5 mmol) in anhydrous THF (300 mL) under N_2-atmosphere at 0 °C was dropwise added DIAD (10.0 mL, 50.8 mmol). The mixture was stirred for 30 min and precipitation of a colourless crystalline solid was observed. Then, a solution of *N*-Boc serinol **9** (4.70 g, 24.3 mmol) in anhydrous THF (25 mL) and HSAc (3.8 mL, 53.5 mmol) were added under vigorous stirring. The reaction mixture was stirred at 0 °C for 1 h, was then allowed to warm to r.t. and further stirred for 16 h, before being concentrated under reduced pressure. The residue was dry-loaded on activation silica gel (Ceduran®) and purified by flash column chromatography (isohexane:EtOAc, 90:10 to 0:100) to give and compound **4** (6.63 g, 21.6 mmol, 88%) as a colourless solid.[46]

Protocol B:

Step 1: To a solution of *N*-Boc serinol (77 mg, 0.40 mmol), and pyridine (157 µL, 1.95 mmol) in anhydrous DCM (10 mL) under N_2-atmosphere at 0 °C was added MsCl (31 µL, 0.40 mmol). The mixture was stirred for 30 min, then warmed to r.t. and stirred further for 1.5 h. After diluting with DCM (20 mL), the mixture was washed with sat. aq. NaCl (2×20 mL) and the aq. washings were extracted with DCM (2×10 mL). The combined organic layers were dried over Na_2SO_4 and concentrated under reduced pressure to *N*-Boc 2-aminopropane-1,3-diyl dimethanesulfonate as a colourless oil, which was used without further purification.

Step 2: The material obtained in step 1 was dissolved in acetone (5 mL), potassium thioacetate (recrystallized from EtOH, 137 mg, 1.20 mmol) was added and the mixture was heated to reflux for 2 h. Then the volatiles were removed under reduced pressure and DCM (10 mL) and H_2O (10 mL) were added. The layers were separated and the aq. layer was extracted with DCM (2×10 mL). The combined organic layers were dried over Na_2SO_4, filtered over a short layer of silica gel and concentrated under reduced pressure to give compound **4** (110 mg, 0.36 mmol, 89%) as a colourless solid.

TLC R_f = 0.36 (*n*-pentane:Et_2O, 3:1). **HRMS** (ESI$^+$): $C_{12}H_{21}NNaO_4S_2^+$ [M+Na]$^+$: calc. 330.08042, found 330.08039. **^{1}H-NMR** (400 MHz, $CDCl_3$): δ (ppm) = 4.88 – 4.62 (m, 1H), 3.99 – 3.80 (m, 1H), 3.07 (d, *J* = 7.0 Hz, 4H), 2.35 (s, 6H), 1.42 (s, 9H).[12]

N-Boc 1,2-dithiolan-4-amine (5)[12]

Bis-thioacetate **4** (4.59 g, 14.9 mmol) was dissolved in a 0.17 M solution of KOH in methanol (500 mL). The mixture was stirred at r.t. open to air for 15 h, concentrated under reduced pressure (at <40 °C) and the remaining yellow oil was dissolved in EtOAc (200 mL). The solution was washed with aq. NaCl (2×50 mL) and H_2O (100 mL), dried over Na_2SO_4 , filtered and concentrated under reduced pressure (at T < 40 °C) to afford compound **5** (3.22 g, 14.6 mmol, 98%) as a yellow solid.[12]

TLC R_f = 0.49 (isohexane:EtOAc, 10:1). **HRMS** (ESI$^-$): $C_8H_{14}NO_2S_2^-$ [M-H]$^-$: calc. 220.04714, found 220.04718. **^{1}H-NMR** (400 MHz, $CDCl_3$): δ (ppm) = 5.06 (d, *J* = 3.9 Hz, 1H, N*H*), 4.95 (s, 1H, C*H*), 3.21 (dd, *J* = 11.6, 4.8 Hz, 2H), 3.09 (dd, *J* = 11.6, 2.0 Hz, 2H), 1.44 (s, 9H).

N-Boc *N*-methyl-1,2-dithiolan-4-amine (10)

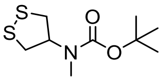

To a mixture of 60% NaH suspension in mineral oil (434 mg, 10.8 mmol), anhydrous DMF (180 mL) and iodomethane (731 µL, 11.7 mmol) under N_2-atmosphere at 0 °C was added dithiolane **5** (2.00 g, 9.04 mmol) in one portion. After stirring at 0 °C for 40 min, H_2O (500 mL) was added and the mixture was extracted with EtOAc (3×250 mL). The extracts were washed with H_2O (200 mL) and aq. NaCl (2×200 mL), dried over Na_2SO_4 and concentrated under reduced pressure.

The remaining yellow oil (3.41 g) was dissolved in methanol (250 mL) and KOH (2.10 g, 37.5 mmol) was added. This "depolymerization mixture" was stirred open to air for 6 days and was extracted four times (after 1 day, 2 days, 4 days and 6 days) to obtain compound **10** (507 + 587 + 307 + 90 mg = 1.49 g, 6.33 mmol, 70%) according to the following procedure:

The depolymerization mixture was extracted with hexanes (2×350 mL). The extracts were washed with half-saturated aq. NaCl (300 mL), dried over Na_2SO_4 and filtered over a plug of silica gel (32 g). The filtrate was discarded and compound **10** was eluted with a 1:8-mixture of EtOAc:hexanes (360 mL). After evaporating the eluent, compound **10** was obtained as a yellow oil. Additionally, the silica was washed with ethyl acetate (200 mL), the washings were evaporated, the remainder was dissolved in small amount of methanol and added back into the depolymerization mixture.

TLC R_f = 0.49 (isohexane:EtOAc 10:1). **HRMS** (EI): $C_9H_{17}NO_2S_2$: [M-e] calc. 235.0696, found 235.0677. **^{1}H-NMR** (400 MHz, $CDCl_3$): δ (ppm) = 5.49 – 4.73 (m, 1H), 3.30 (dd, *J* = 12.0, 7.8 Hz, 2H), 3.03 (dd, *J* = 12.0, 5.9 Hz, 2H), 2.82 (s, 3H), 1.47 (s, 9H). **^{13}C-NMR** (101 MHz, $CDCl_3$): δ (ppm) = 155.3 (*C*=O), 80.5 ($C(CH_3)_3$), 61.2 (*C*H), 40.5 (CH_2), 29.5 (CH_3), 28.5 (CH_3).

N-methyl-1,2-dithiolan-4-amine hydrochloride (6)

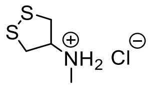

Dithiolane **10** (118 mg, 0.501 mmol) was dissolved in anhydrous MeOH (0.033 M, 15 mL) and stirred open to air for 1 hm before a 1.25 M solution of HCl in MeOH (5 mL, 6.25 mmol) was added. The resulting mixture was heated to reflux for 6 h and then evaporated under reduced pressure to yield hydrochloride **6** (85 mg, 0.50 mmol, quant.) as a colourless solid.

^{1}H-NMR (400 MHz, DMSO-d_6): δ (ppm) = 8.49 (s, 2H), 3.35 (p, *J* = 5.1 Hz, 1H), 2.58 (dd, *J* = 5.2, 1.7 Hz, 4H), 1.74 (s, 3H). **^{13}C-NMR** (101 MHz, DMSO-d_6): δ (ppm) = 63.3 (*C*H), 40.4 (CH_2), 31.1 (CH_3).

1,2-dithiolan-4-ol (13)

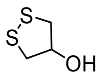

Step 1: To a solution of 4-dimethylaminopyridine (189 mg, 1.55 mmol, 0.1 eq.), pyridine (1.25 mL, 15.5 mmol, 1.0 eq.) and benzoyl chloride (1.98 mL, 17.1 mmol, 1.1 eq.) in anhydrous DCM (40 mL) at 0 °C was dropwise added 1,3-dichloropropan-2-ol **11** (1.48 mL, 15.5 mmol, 1.0 eq.). The mixture was stirred with warming to r.t. for 15 h, diluted with DCM (40 mL), washed with 2 M aq. HCl (40 mL), water (40 mL) and sat. aq. $NaHCO_3$ (40 mL) and dried over $MgSO_4$. The volatiles were evaporated under reduced pressure and the remaining colourless oil was directly used in the next step.

Step 2: The material obtained in step 1 was dissolved in acetone (150 mL) and potassium thioacetate (4.43 g, 38.8 mmol, 2.5 eq.) was added. The mixture was stirred at r.t. for three days, and then at reflux for six more days. While stirring at reflux, more KSAc was added on day four (2.66 g, 23.5 mmol, 1.5 eq.) and day five (2.66 g, 23.5 mmol, 1.5 eq.). After cooling to r.t., the mixture was filtered, the solids were discarded and the filtrate was evaporated under reduced pressure. The residue was suspended in DCM (50 mL), the suspension was filtered, the solids were discarded and the filtrate was evaporated under reduced pressure. The residue was purified by FCC (silica, DCM/isohexane) to afford a dark red oil containing *O*-benzoyl-bis(thioacetate) **12** (1.38 g, 4.42 mmol, 29%).

Step 3: The material obtained in step 2 was dissolved in MeOH (150 mL), KOH (1.24 g, 22.1 mmol, 5.0 eq.) was added and the mixture was stirred at r.t. under air for seven days. Then, the mixture was filtered and the solids were discarded. The filtrate was diluted with diethyl ether (200 mL), washed with water (200 mL), 2 M aq. NaOH

(150 mL) and sat. aq. NaCl (100 mL) and dried over $MgSO_4$. The volatiles were removed under reduced pressure and the remaining crude 1,2-dithiolan-4-ol **13** (60 mg, 0.49 mmol, 11%) was used without further purification.

4-chloro-2-(6-chloro-4-oxo-3,4-dihydroquinazolin-2-yl)phenyl (1,2-dithiolan-4-yl)(methyl)carbamate (SS50-PQ, 1)

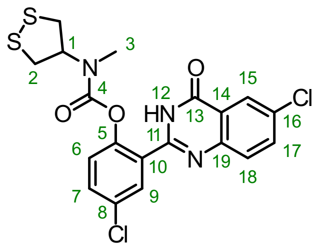

Step 1: To a solution of **PQ-OH (2)** (Prepared according to procedures reported in Felber *et al.*[14]) (57.2 mg, 0.187 mmol) in anhydrous DCM (5 mL) under nitrogen atmosphere at 0 °C was added a solution of triphosgene (60.5 mg, 0.204 mmol) in anhydrous DCM (1 mL), followed by a solution of DIPEA (38 µL, 0.221 mmol) in anhydrous DCM (2 mL). The resulting mixture was stirred at 0 °C for 30 min, then warmed to r.t. and stirred further for 30 min, before being evaporated under high vacuum (the volatiles were condensed in a liquid nitrogen trap and treated with 2M aq. NaOH as well as piperidine to discharge residual phosgene). The remainder was dissolved in anhydrous DCM (12.5 mmol) and this solution of **PQ-OH**-chloroformate was directly used in the next reaction.

Step 2: To a suspension of compound **6** (29.2 mg, 0.170 mmol) in anhydrous DCM (2 mL) was added DIPEA (34 µL, 0.20 mmol), the resulting solution was added dropwise at 0 °C to the solution of **PQ-OH** chloroformate obtained in step 1 and the mixture was stirred at 0°C for 15 min. Then, additional DIPEA (29 µL, 0.17 mmol) was added, before the mixture was warmed to ambient temperature. After stirring for 30 min, the mixture was concentrated under reduced pressure and the residue was purified by FCC (isohexane:EtOAc, 90:10 to 0:100) to give compound **1** (39.0 mg, 0.083 mmol, 49%) as a colourless solid.

Only weak, blue residual fluorescence was observed upon irradiation with UV-light, confirming that the obtained material was not contaminated with **PQ-OH**.

TLC R_f = 0.26 (isohexane:EtOAc, 3:1).

HRMS (ESI$^+$): $C_{19}H_{16}Cl_2N_3O_3S_2^+$ [M+H]$^+$: calc. 468.00046, found 468.00049.

Individual rotamers were observed by NMR spectroscopy at 298 K (ratio ≈ 2/1) and time-averaged spectra were obtained at 373 K.

^{1}H-NMR (400 MHz, tetrachlorethan-d_2, 373 K): δ (ppm) = 8.24 – 8.22 (m, 1H, 15-H), 7.95 (d, *J* = 2.6 Hz, 1H, 9-H), 7.71 (d, *J* = 1.9 Hz, 2H, 17-H; 18-H), 7.50 (dd, *J* = 8.7, 2.7 Hz, 1H, 7-H), 7.20 (d, *J* = 8.7 Hz, 1H, 6-H), 5.09 (p, *J* = 6.7 Hz, 1H, 1-H_{ax}), 3.32 (dd, $^2J_{2'-2}$ = 12.1 Hz, $^3J_{2-1}$ = 7.6 Hz, 2H, 2-H_{ax}), 3.10 (dd, $^2J_{2'-2}$ = 12.1 Hz, $^3J_{2'-1}$ = 5.6 Hz, 2H, 2'-H_{eq}), 3.04 (s, 3H, 3-H).

^{13}C-NMR (101 MHz, tetrachlorethan-d_2, 373 K): δ (ppm) = 160.1 (*C*=O, C13), 153.7 (*C*=O, C4), 149.0 (*C*=N, C11), 147.6 (C_{Ar}, C5), 147.4 (C_{Ar}, C14), 135.3 (C_{Ar}H, C18), 133.6 (C_{Ar}, C19), 132.3 (C_{Ar}H, C7), 132.1 (C_{Ar}, C8), 130.4 (C_{Ar}H, C9), 129.7 (C_{Ar}H, C17), 128.0 (C_{Ar}, C10), 126.1 (C_{Ar}H, C15), 125.2 (C_{Ar}H, C6), 122.4 (C_{Ar}, C16), 62.9 (*C*H, C1), 40.7 (CH_2, C2), 30.6 (CH_3, C3).

1,2-dithiolan-4-yl (2-butyl-1,3-dioxo-2,3-dihydro-1H-benzo[de]isoquinolin-6-yl)carbamate (TRFS-green) (15)

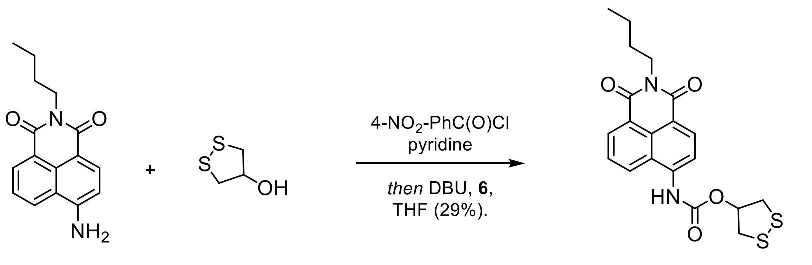

14 **6** **TRFS-green (15)**

To a solution of 4-nitrophenyl chloroformate (78 mg, 0.39 mmol, 1.1 eq.) in anhydrous tetrahydrofuran (3 mL) under nitrogen atmosphere at 0 °C was dropwise added a solution of 1-butyl-6-amino-naphtalimide **14** (prepared according to reported procedures[8,47,48] (94 mg, 0.35 mmol, 1.0 eq.) and pyridine (42 µL, 0.52 mmol, 1.5 eq.) in anhydrous tetrahydrofuran (3 mL). The mixture was stirred with warming to room temperature for 4 hours. Then, a solution of 1,2-dithiolan-4-ol (51 mg, 0.42 mmol, 1.2 eq.) in anhydrous tetrahydrofuran (3 mL) and diazabicycloundecene (157 µL, 1.05 mmol, 3.0 eq.) were added. The mixture was stirred at room temperature overnight and concentrated to dryness under reduced pressure. The residue was taken up in EtOAc (40 mL), washed with water (30 mL) and sat. aq. NaCl (30 mL) and dried over $MgSO_4$. The volatiles were removed under reduced pressure and the residue was subjected to FCC (silica, isohexane/EtOAc), which afforded a yellow solid (113 mg) consisting of **TRFS-green**, 4-nitrophenol and other minor impurities. This solid was washed with acetonitrile, which afforded pure **TFRS-green** (**15**) (35 mg, 0.084 mmol, 24%) as a yellow solid.

TLC R_f = 0.73 (isohexane:EtOAc, 1:1).

HRMS (ESI^+): $C_{20}H_{21}N_2O_4S_2$ $[M+H]^+$ calc. 417.0938, found 417.0938.

^{1}H-NMR (400 MHz, $CDCl_3$): 8.64 (dd, *J* = 7.3, 1.0 Hz, 1H), 8.61 (d, *J* = 8.2 Hz, 1H), 8.35 (d, *J* = 8.2 Hz, 1H), 8.20 (dd, *J* = 8.6, 1.1 Hz, 1H), 7.79 (dd, *J* = 8.6, 7.3 Hz, 1H), 7.53 (s, 1H), 5.90 (m, 1H), 4.22 – 4.13 (m, 2H), 3.45 (dd, *J* = 12.7, 5.3 Hz, 2H), 3.38 (dd, *J* = 12.8, 2.6 Hz, 2H), 1.78 – 1.66 (m, 2H), 1.44 (h, *J* = 7.4 Hz, 2H), 0.97 (t, *J* = 7.4 Hz, 3H).

^{13}C-NMR (100 MHz, $CDCl_3$): 164.2, 163.7, 152.3, 138.4, 132.5, 131.5, 129.1, 127.0, 125.9, 123.7, 123.0, 118.5, 117.0, 79.7, 45.2, 40.4, 30.3, 20.5, 14.0.

Apart from all ^{13}C signals being shifted slightly (presumed to result from different referencing), the obtained spectroscopic data was in agreement with previously reported values. See Zhang *et al., J. Am. Chem. Soc.* **2014**, *136*, 226.[8] Upon comparing our spectroscopic data with the previously reported values we noticed that the data reported by Zhang *et al.* contained some mistakes. The ^{13}C-NMR data reported in the text is missing a signal around 79 ppm, that was however present in the spectrum depicted in the Supplementary information. Furthermore, the ^{1}H NMR data refers to a doublet of doublets at 8.63 ppm, which actually consists of two clearly separated signals corresponding to two non-equivalent protons.

1-(1,2-dithiolan-4-yl)-3-(2-oxo-4-(trifluoromethyl)-2*H*-chromen-7-yl)urea (Fast-TRFS) (18)

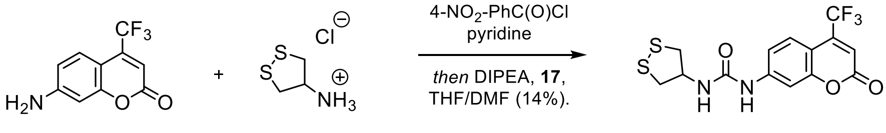

16 **17** **Fast-TRFS (18)**

To a solution of 4-nitrophenyl chloroformate (78 mg, 0.39 mmol, 1.1 eq.) in anhydrous tetrahydrofuran (3 mL) under nitrogen atmosphere at 0 °C was dropwise added a solution of compound 7-amino-4-(trifluoromethyl)-2*H*-chromen-2-one **16** (prepared according to Sun *et al.*[49] (80 mg, 0.35 mmol, 1.0 eq.) and pyridine (42 µL, 0.52 mmol, 1.5 eq.) in anhydrous THF (3 mL). The mixture was stirred with warming to r.t. for 4 h. Then, a suspension consisting of 1,2-dithiolan-4-amine hydrochloride **17** (prepared according to Felber *et al.*[14]) (66 mg, 0.42 mmol, 1.2 eq.), DIPEA (245 µL, 1.4 mmol, 4.0 eq.) and anhydrous DMF (1.5 mL) was added. The mixture was stirred at r.t. overnight and concentrated under reduced pressure. The residue was taken up in EtOAc (40 mL), washed with water (30 mL) and sat. aq. NaCl (30 mL) and dried over $MgSO_4$. The volatiles were removed under reduced pressure and the residue was subjected to FCC (silica, isohexane/EtOAc), which afforded a yellowish solid (51 mg) that consisted of **Fast-TRFS** and minor impurities. This solid was dissolved in warm (approx. 50 °C) MeCN, the solution was cooled to –20 °C overnight and the resulting precipitate was collected to afford pure **Fast-TRFS** (**18**) (18 mg, 0.048 mmol, 14%) as a faint yellow solid.

TLC R_f = 0.6 (isohexane:EtOAc, 1:1).

HRMS (ESI+): $C_{14}H_{12}F_3N_2O_3S_2$ $[M+H]^+$ calc. 377.0236, found 377.0237.

^{1}H-NMR (400 MHz, DMSO-d_6): 9.37 (s, 1H), 7.75 (d, *J* = 2.1 Hz, 1H), 7.59 (dd, *J* = 8.9, 2.0 Hz, 1H), 7.27 (dd, *J* = 8.9, 2.2 Hz, 1H), 6.81 (s, 1H), 6.68 (d, *J* = 8.1 Hz, 1H), 4.96 (dtt, *J* = 7.8, 5.1, 2.7 Hz, 1H), 3.30 (dd, *J* = 11.5, 4.9 Hz, 2H), 3.16 (dd, *J* = 11.5, 2.7 Hz, 2H).

^{13}C-NMR (100 MHz, DMSO-d_6): 158.9, 155.2, 153.7, 144.8, 139.4 (q, *J* = 32.2 Hz), 125.4, 121.8 (q, *J* = 275.6 Hz), 114.8, 113.0 (q, *J* = 5.7 Hz), 106.6, 104.2, 55.6, 44.1.

The previously reported spectroscopic data by Li *et al.*[12] contained significant errors, which complicated a comparison with our spectroscopic data. Upon close inspection of the ^{1}H-NMR spectral data, we noticed that the values provided by Li et al. did not correspond to DMSO-d_6 as specified, but instead were based on a measurement in $CDCl_3$. We did not further characterize our material in $CDCl_3$, as the reported ^{1}H-NMR spectrum in $CDCl_3$ by Li et al. was clearly not pure and we already had obtained a high quality ^{1}H-NMR spectrum in DMSO-d_6, which confirmed that our batch of **Fast-TRFS** was pure. Furthermore, the ^{13}C-NMR spectrum in DMSO-d_6 was in agreement with the previously reported values. However, regarding the ^{13}C-NMR spectrum, Li *et al.* have ignored the splitting of several signals that resulted from the presence of fluorine in the molecule and consequently several chemical shifts were incorrectly reported. Additionally, the ^{13}C-NMR signals were shifted in an absolute, but not in a relative manner, which we suspect to be the result of incorrect referencing. Despite these issues, we were able to match all the signal that had been reported by Li *et al.* and thereby confirmed that we had indeed prepared the same compound.

1-methyl-1-(2-((2-morpholinoethyl)disulfaneyl)ethyl)-3-(2-oxo-4-(trifluoromethyl)-2H-chromen-7-yl)urea (Linear-TRFS) (20)

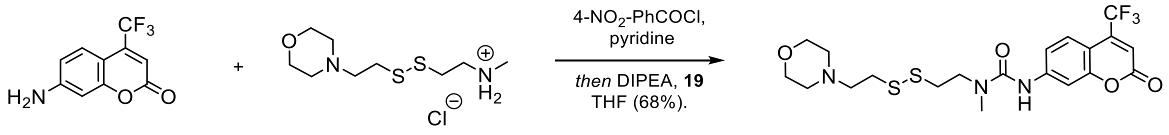

16 19 Linear-TRFS (20)

To a solution of 4-nitrophenyl chloroformate (97 mg, 0.48 mmol, 1.1 eq.) in anhydrous tetrahydrofuran (4 mL) under nitrogen atmosphere at 0 °C was dropwise added a solution of compound 7-amino-4-(trifluoromethyl)-2*H*-chromen-2-one **16** (100 mg, 0.44 mmol, 1.0 eq.) and pyridine (53 µL, 0.66 mmol, 1.5 eq.) in anhydrous THF (4 mL). The mixture was stirred with warming to r.t. for 4 h. Then, solid *N*-methyl-2-((2-morpholinoethyl)disulfaneyl)ethan-1-amine dihydrochloride (prepared according to Felber *et al.*[14]) **19** (162 mg, 0.52 mmol, 1.2 eq.) and DIPEA (608 µL, 3.5 mmol, 8.0 eq.) were added. The mixture was stirred at r.t. overnight, diluted with 30 mL of sat. aq. $NaHCO_3$ and extracted with DCM (3×30 mL). The combined organic phases were dried over $MgSO_4$, the volatiles were removed under reduced pressure and the residue was subjected to FCC (silica, isohexane/EtOAc) to afford pure **Linear-TRFS** (**20**) (145 mg, 0.30 mmol, 68%) as a faint yellow solid.

TLC R_f = 0.33 (acetone:EtOAc, 1:9).

HRMS (ESI^+): $C_{20}H_{25}F_3N_3O_4S_2$ $[M+H]^+$ calc. 492.1233, found 492.1234.

^{1}H-NMR (400 MHz, $CDCl_3$): 7.65 (s, 1H), 7.61 (dq, *J* = 8.9, 2.0 Hz, 1H), 7.44 (d, *J* = 8.9 Hz, 1H), 7.11 (s, 1H), 6.64 (s, 1H), 3.80 – 3.68 (m, 6H), 3.12 (s, 3H), 3.01 – 2.85 (m, 4H), 2.75 (s, 2H), 2.54 (s, 4H).

^{13}C-NMR (100 MHz, $CDCl_3$): 159.7, 155.5, 154.5, 144.5, 141.64 (q, *J* = 32.9 Hz), 125.89 (q, *J* = 2.1 Hz), 121.70 (q, *J* = 275.5 Hz), 116.2, 112.96 (q, *J* = 5.7 Hz), 108.3, 106.8, 66.6, 57.9, 53.5, 48.7, 36.6, 35.7, 35.3.

4 NMR Spectra

***N*-Boc 1,2-dithiolan-4-amine (5)**

^{1}H-NMR

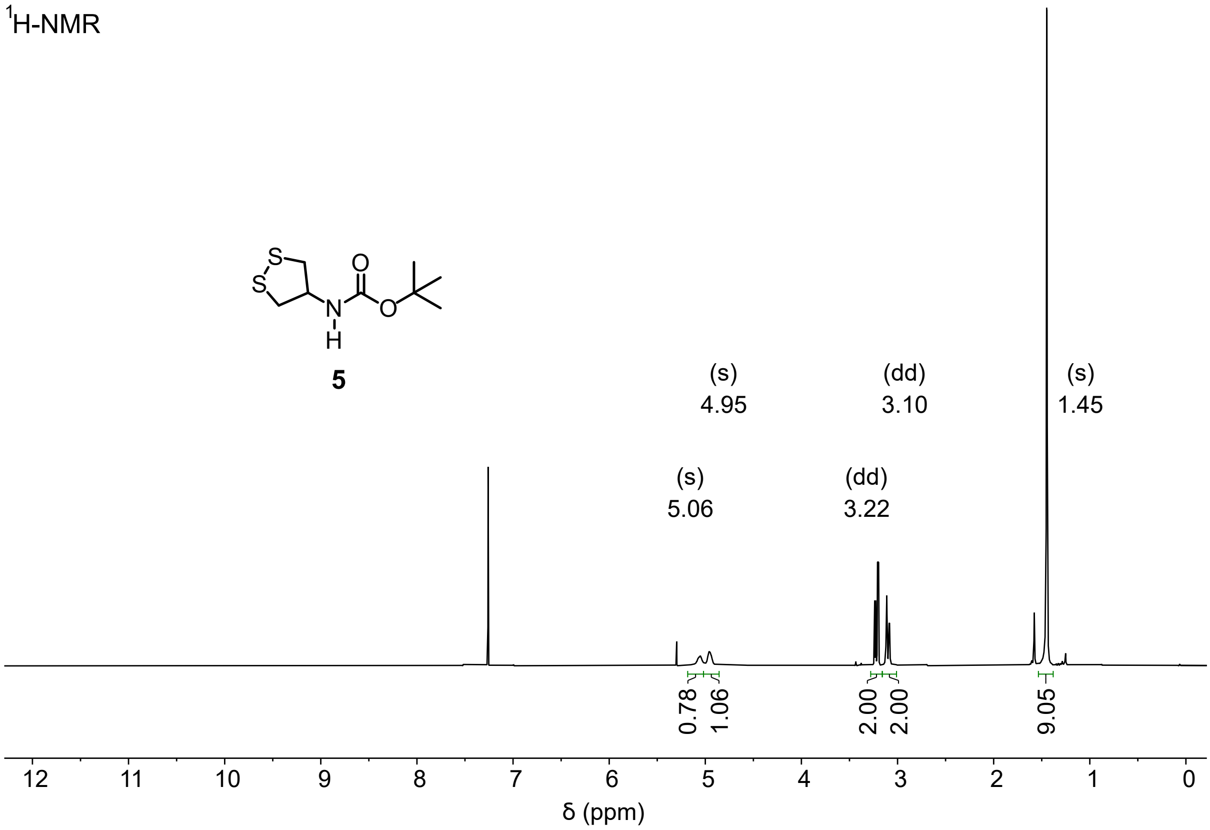

^{13}C-NMR

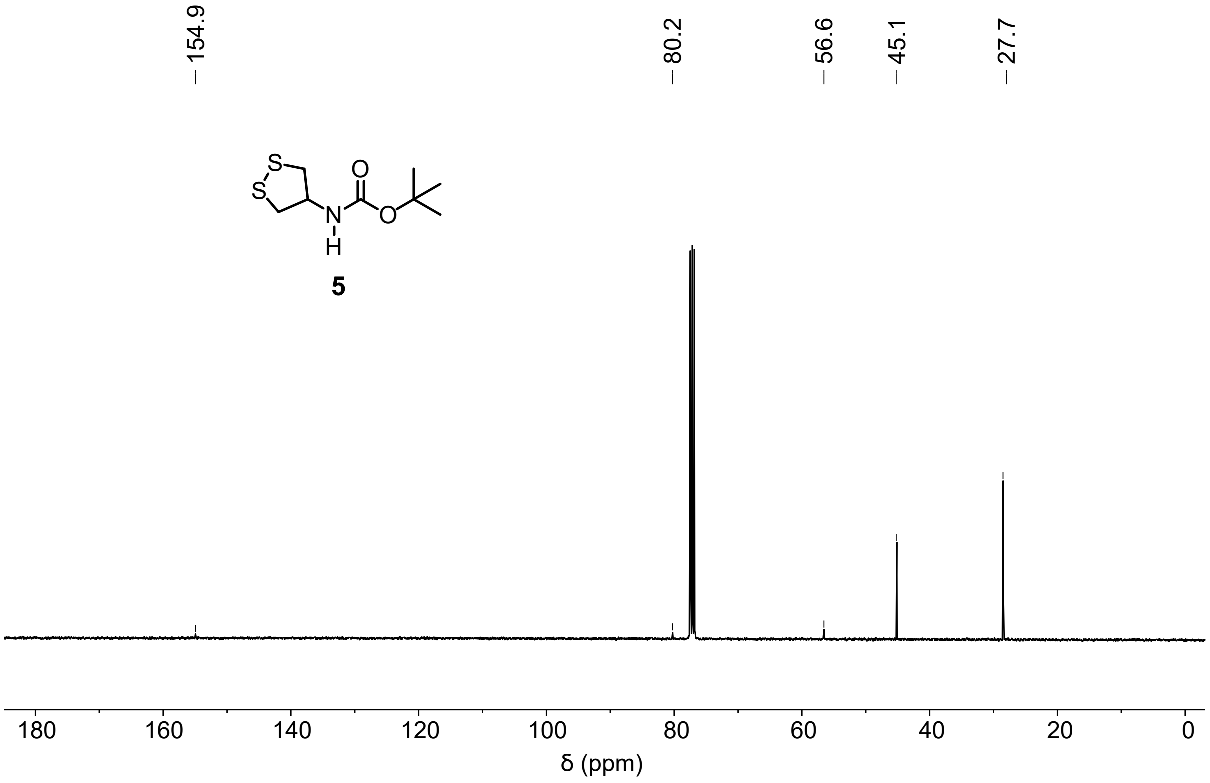

***N*-Boc *N*-methyl-1,2-dithiolan-4-amine (10)**

^{1}H-NMR

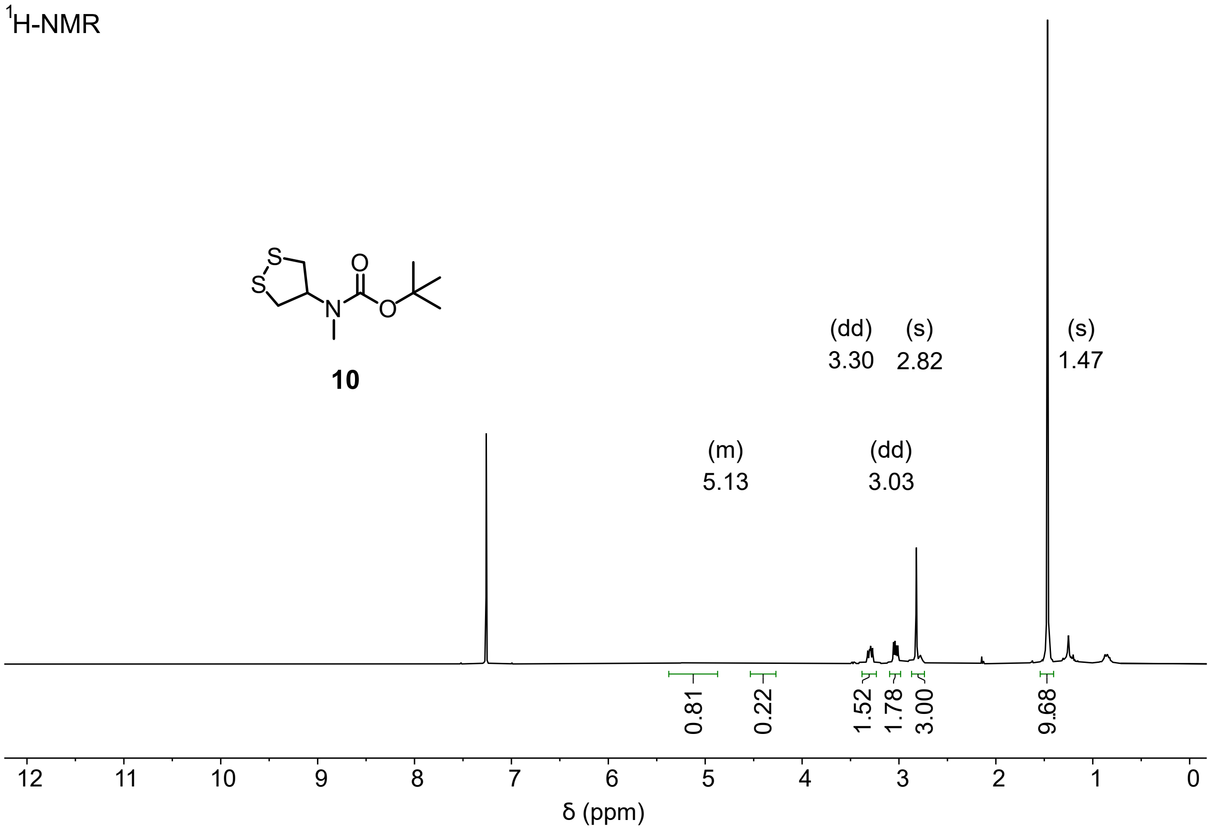

^{13}C-NMR

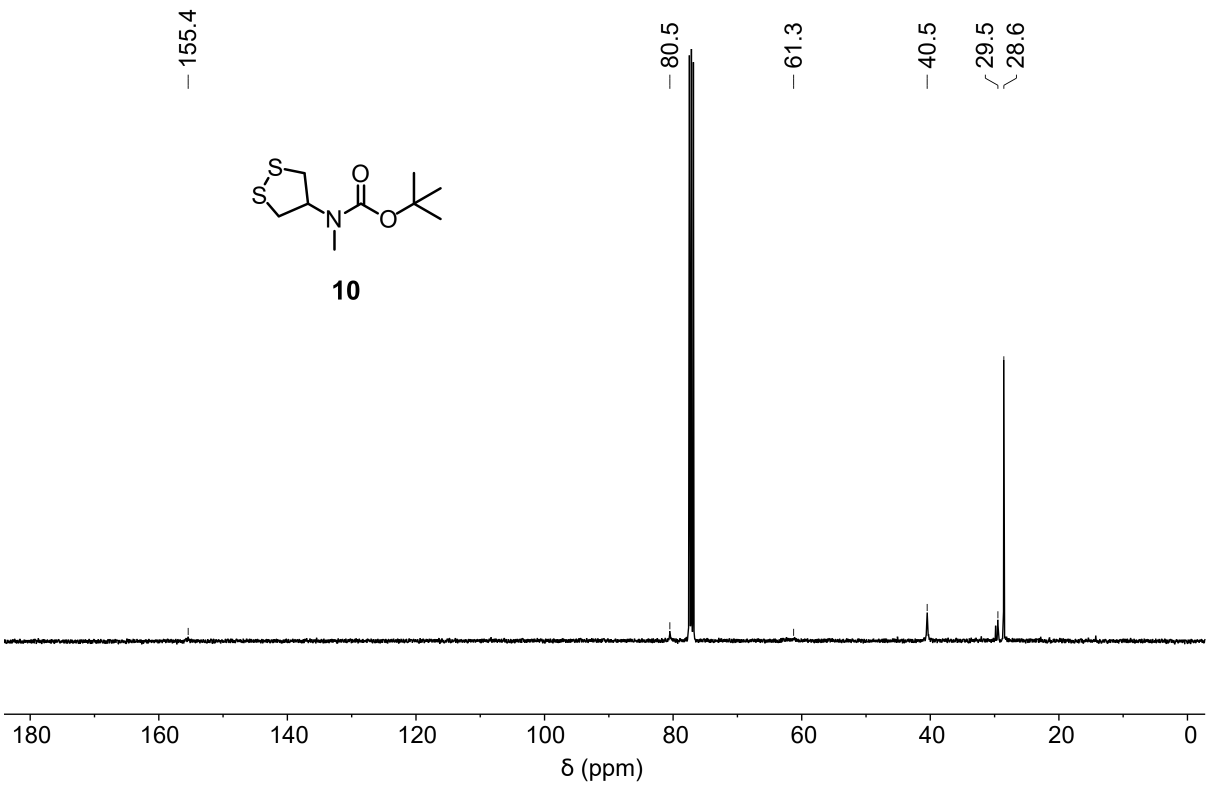

***N*-methyl-1,2-dithiolan-4-amine hydrochloride (6)**

^{1}H-NMR

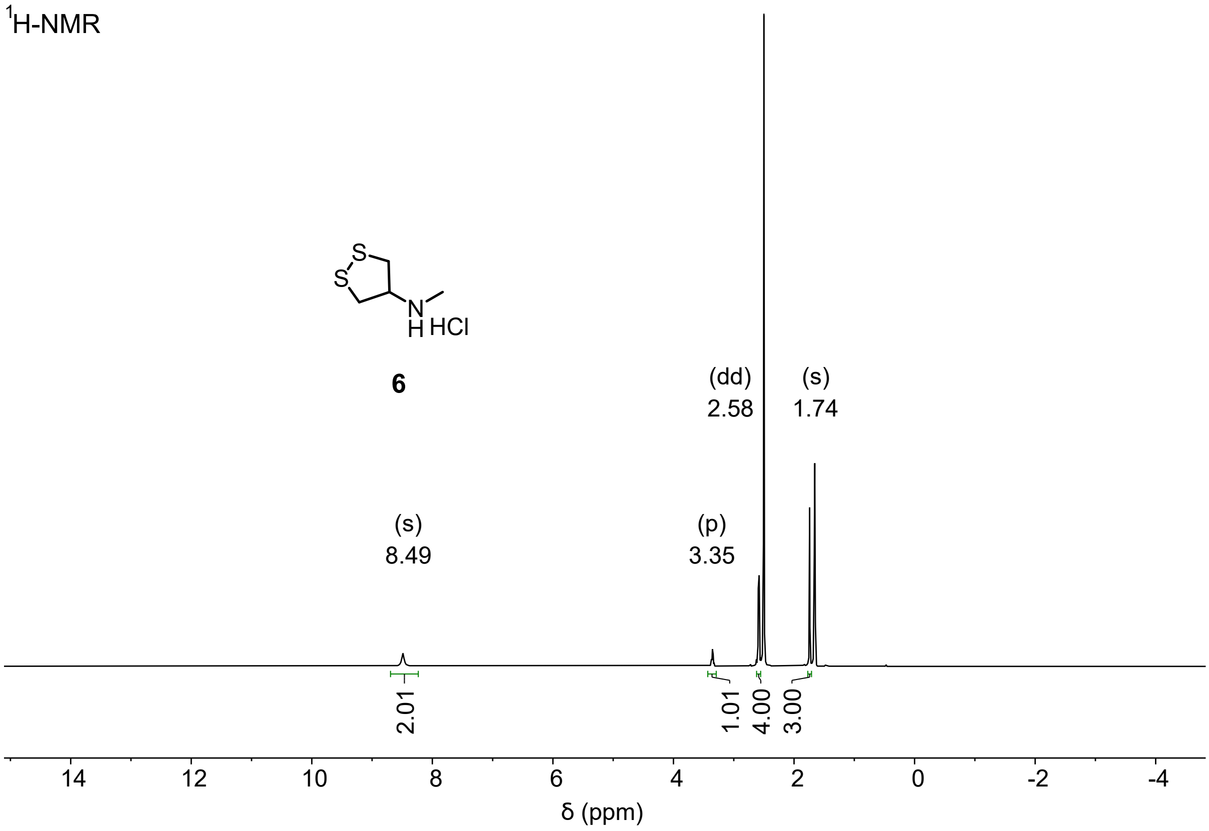

^{13}C-NMR

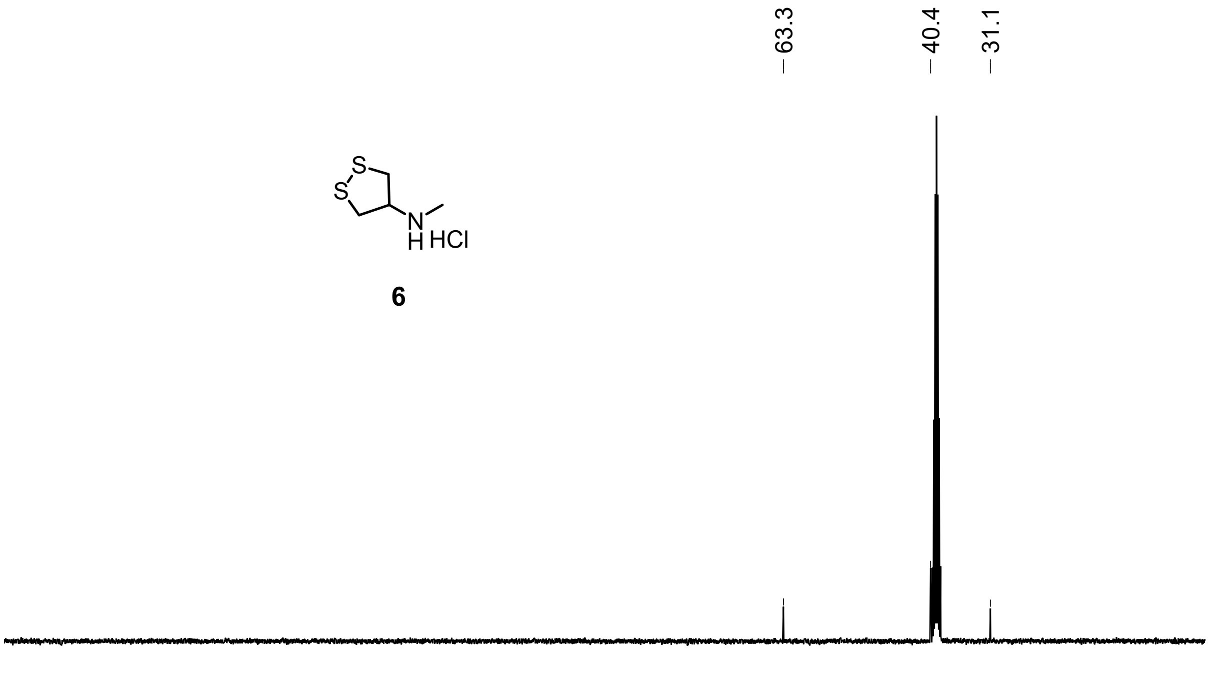

SS50-PQ (1)

^{1}H-NMR (373 K)

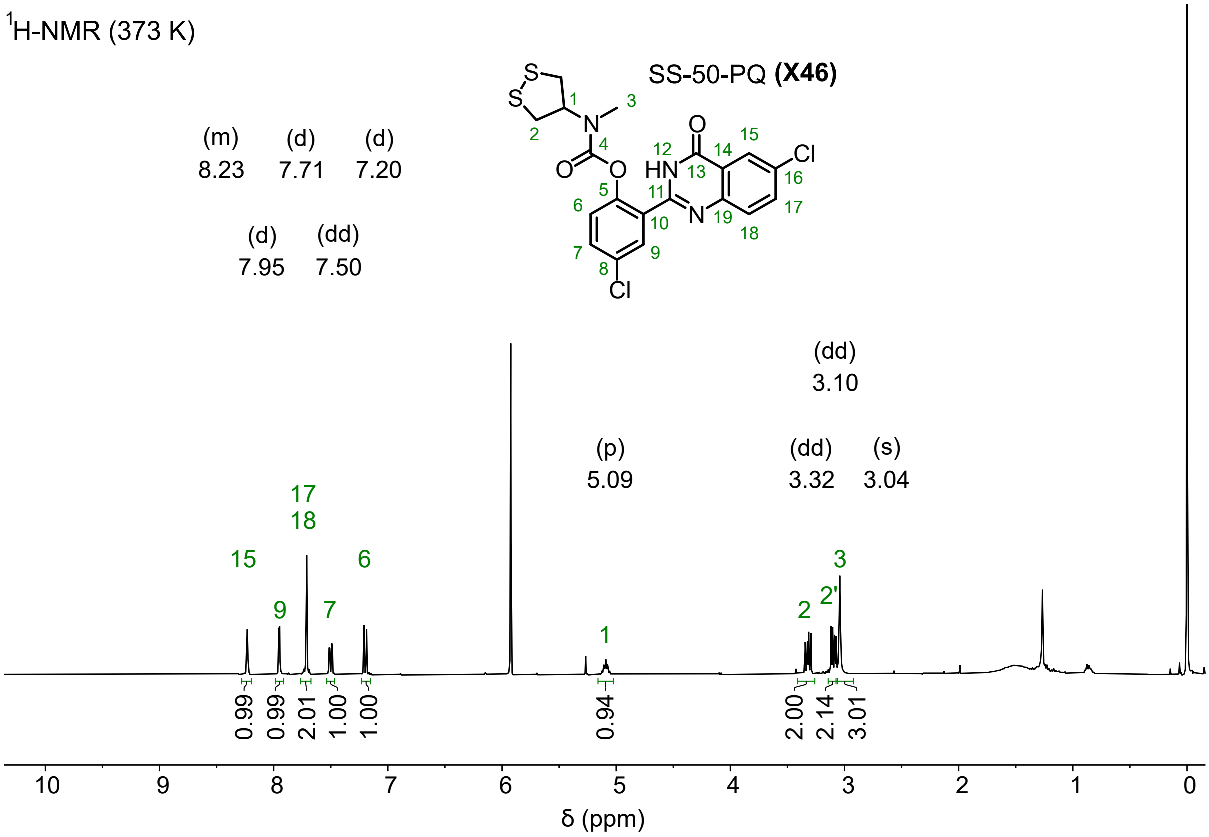

^{13}C-NMR (373 K)

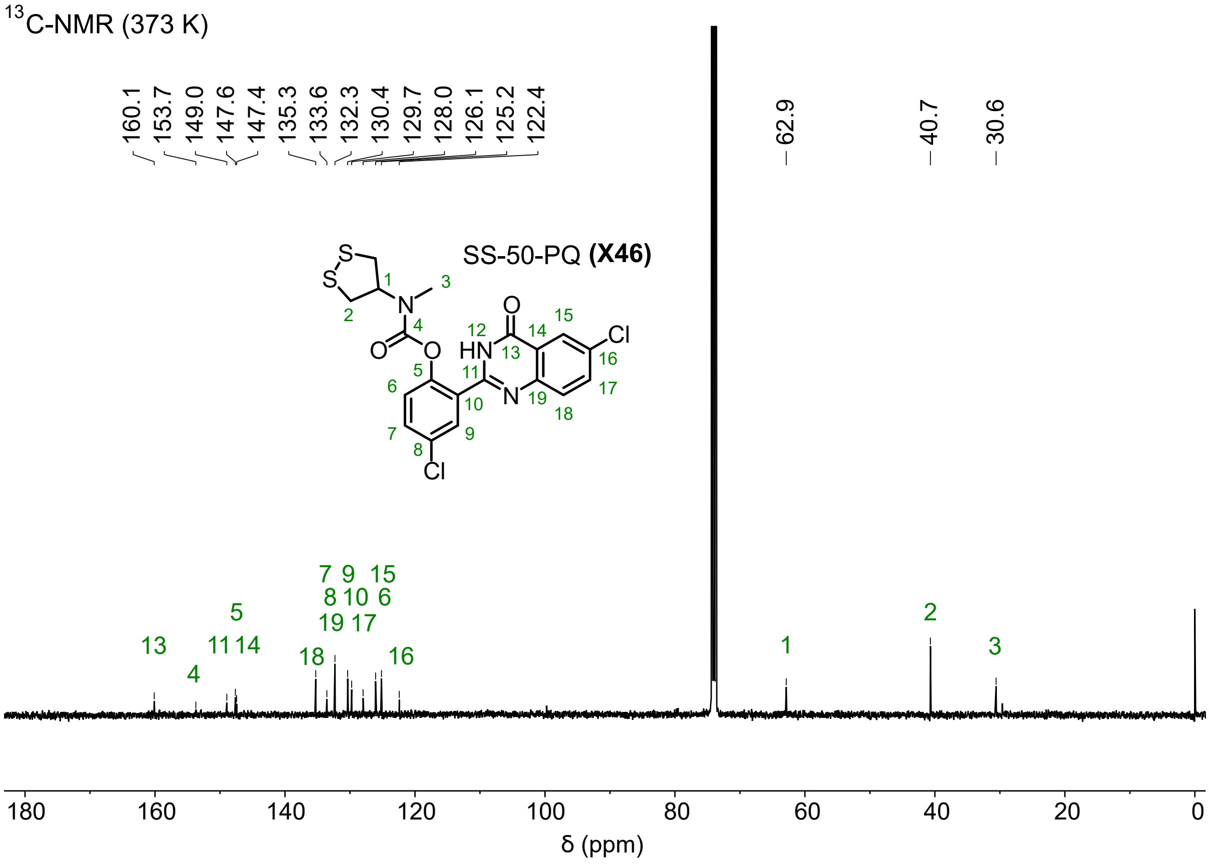

TRFS-green (15)

^{1}H-NMR

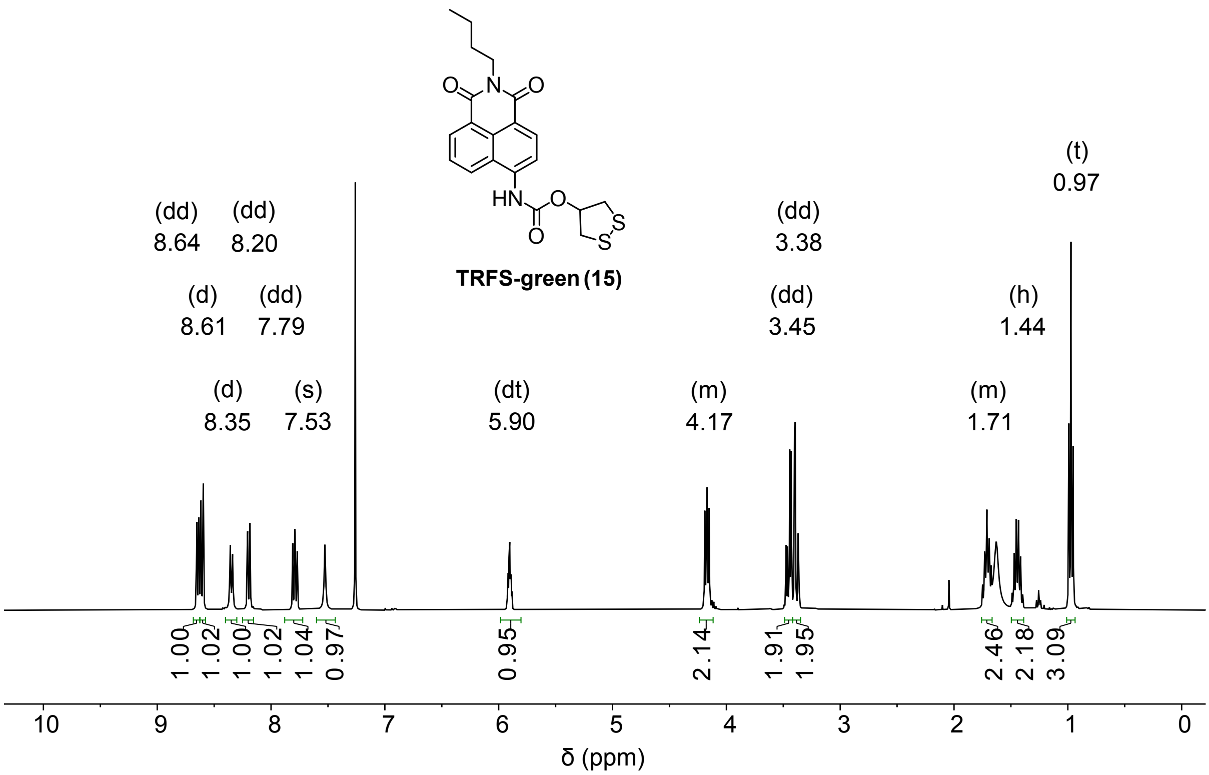

^{13}C-NMR

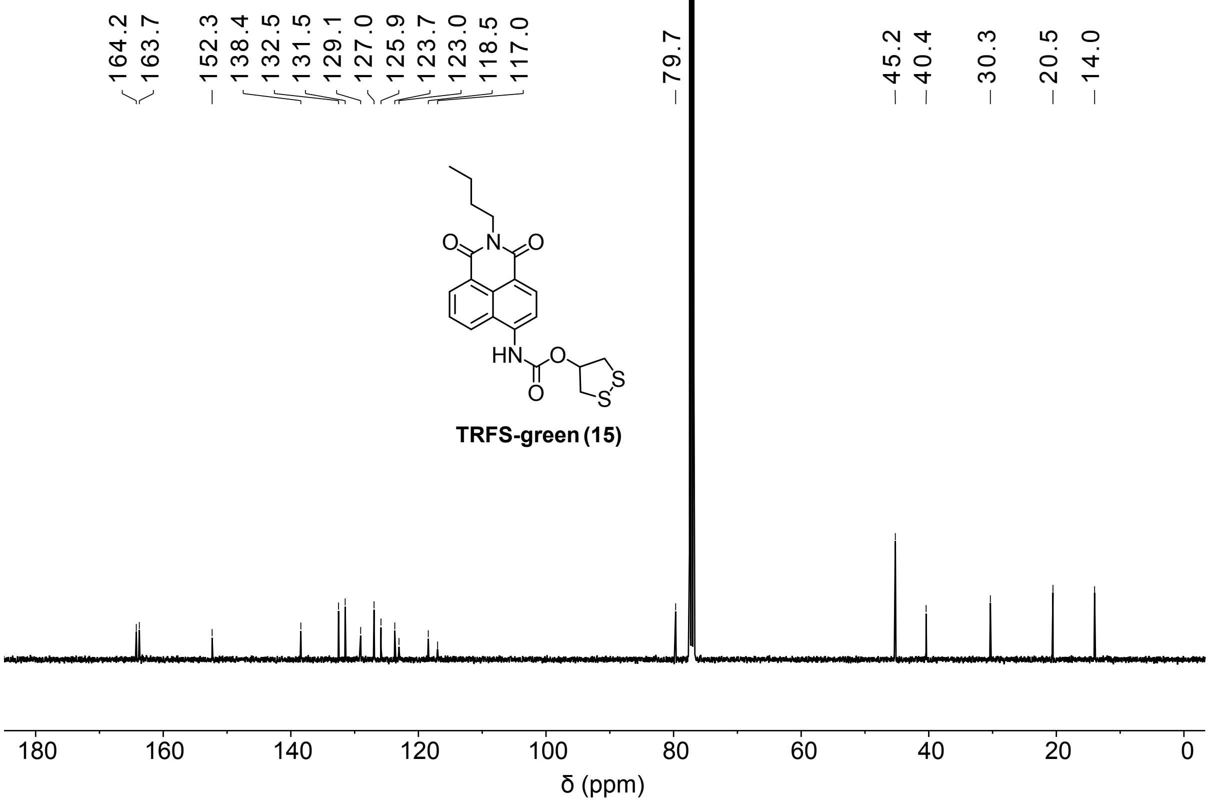

Fast-TRFS (18)

^{1}H-NMR

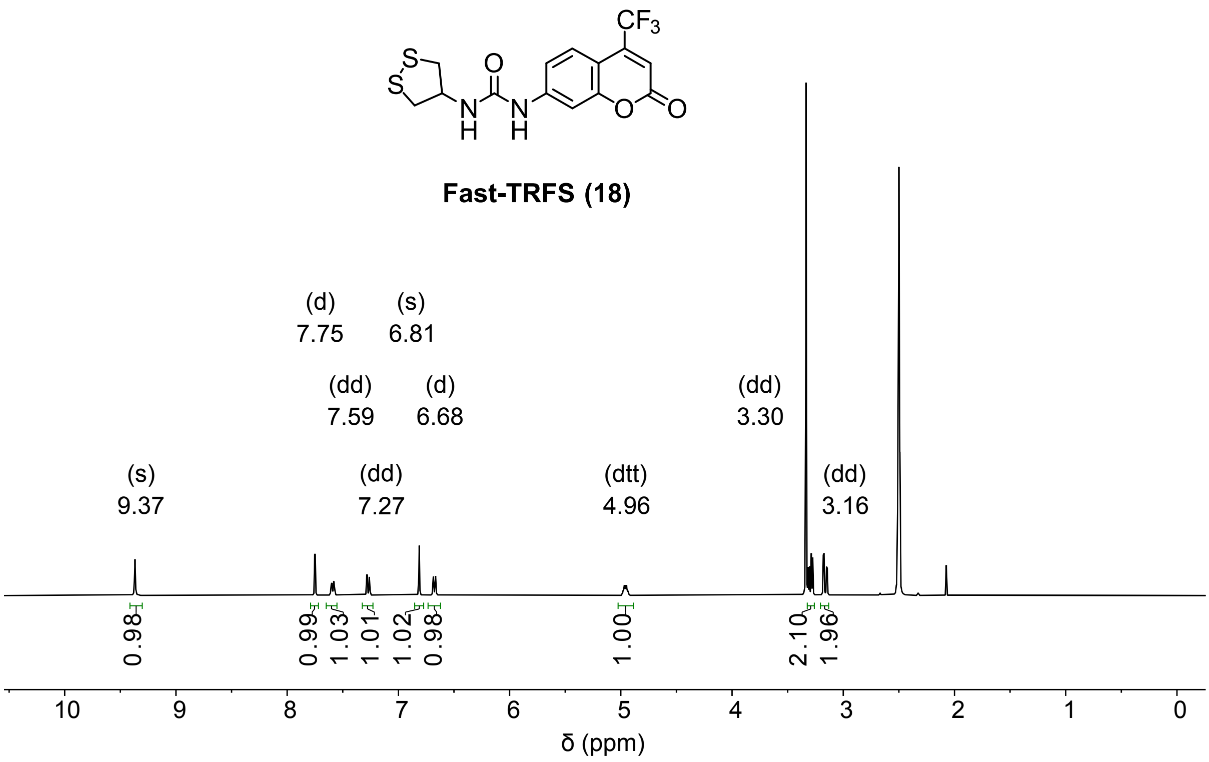

^{13}C-NMR

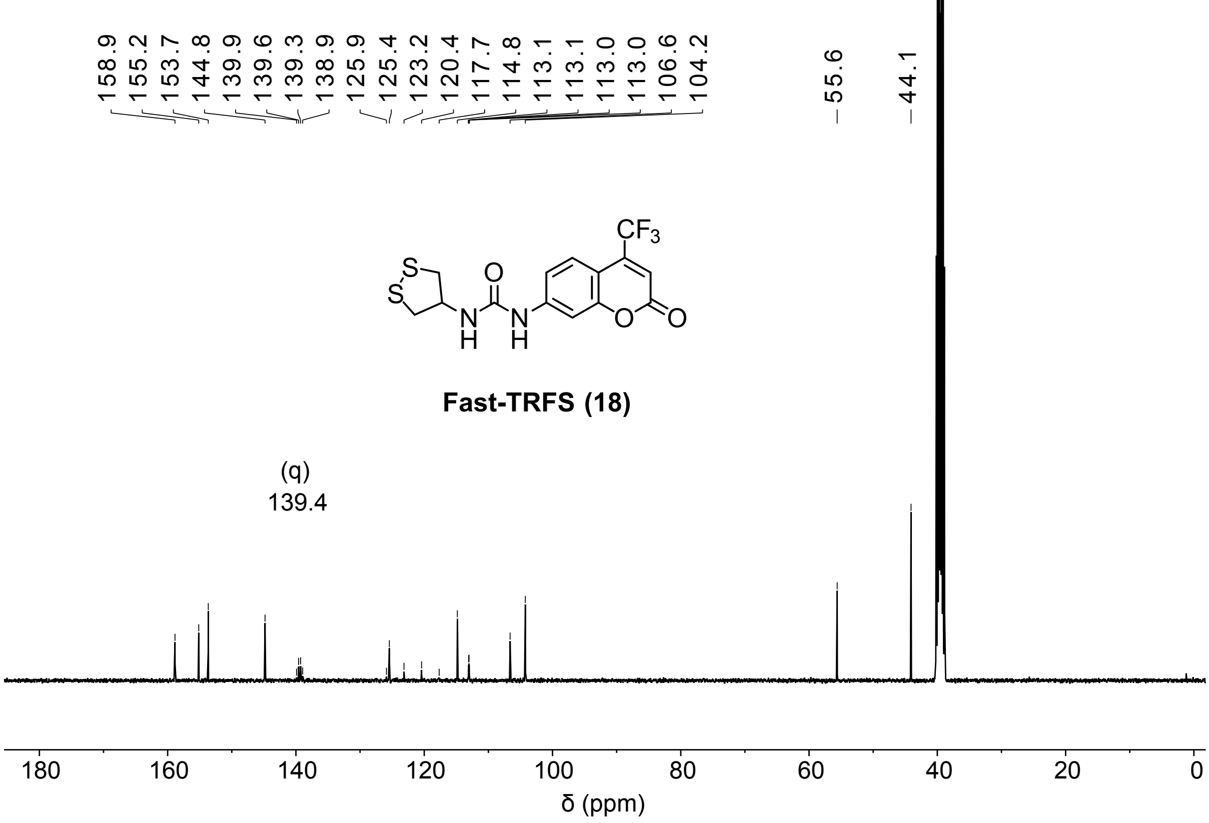

Linear-TRFS (20)

^{1}H-NMR

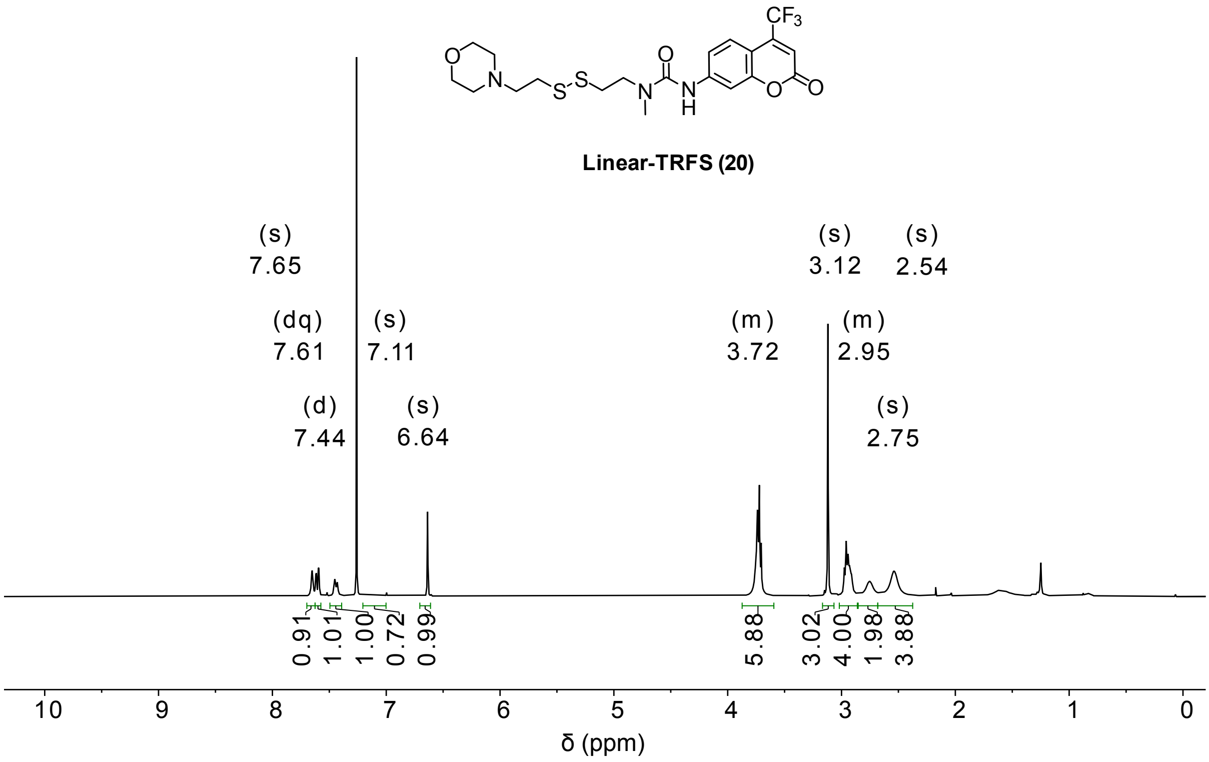

^{13}C-NMR

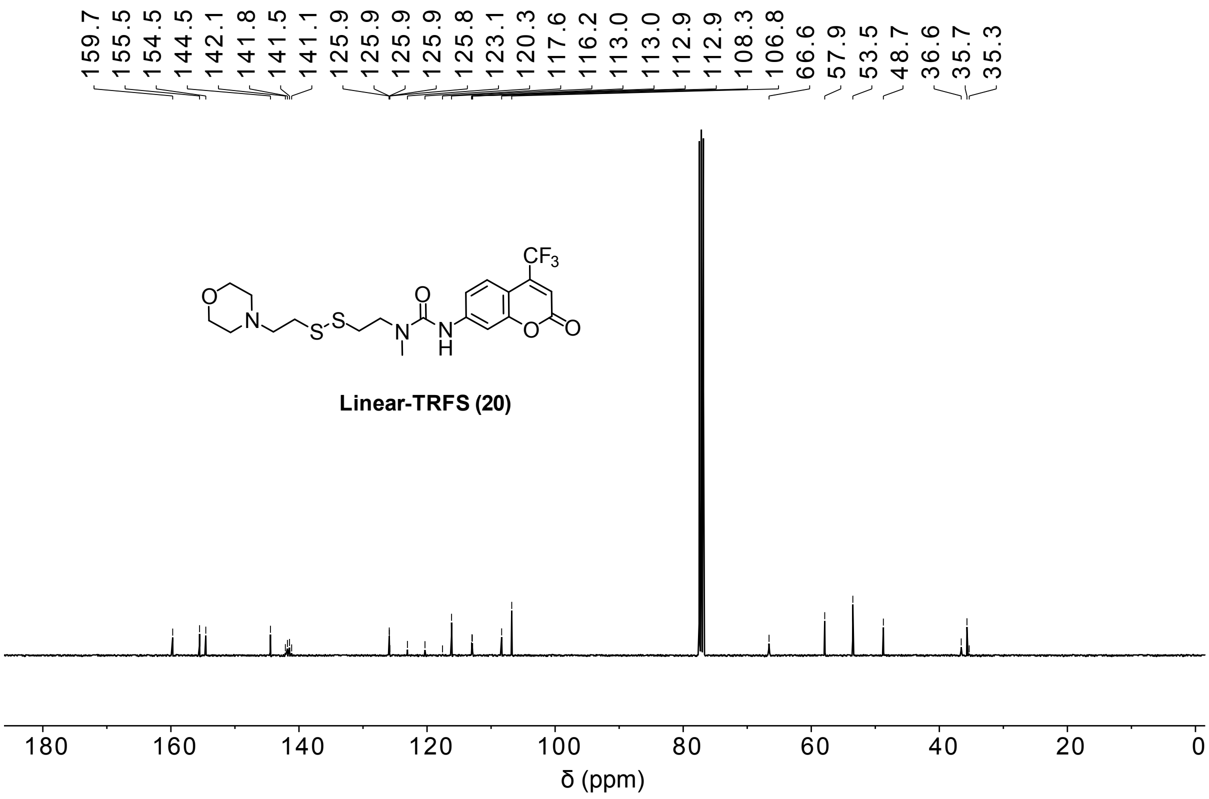

5 Supplementary References

1. Busker, S. *et al.* Irreversible TrxR1 inhibitors block STAT3 activity and induce cancer cell death. *Sci. Adv.* **6**, eaax7945 (2020). https://doi.org/10.1126/sciadv.aax7945.

2. Zeisel, L. *et al.* Selective cellular probes for mammalian thioredoxin reductase TrxR1: rational design of RX1, a modular 1,2-thiaselenane redox probe. *ChemRxiv* (2021). https://doi.org/10.33774/chemrxiv-2021-52kwx.

3. Carmine, A., Domoto, Y., Sakai, N. & Matile, S. Comparison of Lipoic and Asparagusic Acid for Surface-Initiated Disulfide-Exchange Polymerization. *Chem. - Eur. J.* **19**, 11558–11563 (2013). https://doi.org/10.1002/chem.201301567.

4. Gasparini, G., Sargsyan, G., Bang, E.-K., Sakai, N. & Matile, S. Ring Tension Applied to Thiol-Mediated Cellular Uptake. *Angew. Chem. Int. Ed.* **54**, 7328–7331 (2015). https://doi.org/10.1002/anie.201502358.

5. Abegg, D. *et al.* Strained Cyclic Disulfides Enable Cellular Uptake by Reacting with the Transferrin Receptor. *J. Am. Chem. Soc.* **139**, 231–238 (2017). https://doi.org/10.1021/jacs.6b09643.

6. Chuard, N. *et al.* Strain-Promoted Thiol-Mediated Cellular Uptake of Giant Substrates: Liposomes and Polymersomes. *Angew. Chem. Int. Ed.* **56**, 2947–2950 (2017). https://doi.org/10.1002/anie.201611772.

7. Martinent, R., Du, D., López-Andarias, J., Sakai, N. & Matile, S. Oligomers of Cyclic Oligochalcogenides for Enhanced Cellular Uptake. *ChemBioChem* **21**, 1–8 (2020). https://doi.org/10.1002/cbic.202000630.

8. Zhang, L. *et al.* Highly Selective Off–On Fluorescent Probe for Imaging Thioredoxin Reductase in Living Cells. *J. Am. Chem. Soc.* **136**, 226–233 (2014). https://doi.org/10.1021/ja408792k.

9. Liu, Y. *et al.* A small molecule probe reveals declined mitochondrial thioredoxin reductase activity in a Parkinson's disease model. *Chem. Commun.* **52**, 2296–2299 (2016). https://doi.org/10.1039/C5CC09998F.

10. Ma, H., Zhang, J., Zhang, Z., Liu, Y. & Fang, J. A fast response and red emission probe for mammalian thioredoxin reductase. *Chem. Commun.* **52**, 12060–12063 (2016). https://doi.org/10.1039/C6CC04984B.

11. Li, X. *et al.* Selective Activation of a Prodrug by Thioredoxin Reductase Providing a Strategy to Target Cancer Cells. *Angew. Chem. Int. Ed.* **57**, 6141–6145 (2018). https://doi.org/10.1002/anie.201801058.

12. Li, X. *et al.* A fast and specific fluorescent probe for thioredoxin reductase that works via disulphide bond cleavage. *Nat. Commun.* **10**, 1–12 (2019). https://doi.org/10.1038/s41467-019-10807-8.

13. Zhao, J. *et al.* Loss of thioredoxin reductase function in a mouse stroke model disclosed by a two-photon fluorescent probe. *Chem. Commun.* **56**, 14075–14078 (2020). https://doi.org/10.1039/D0CC05900E.

14. Felber, J. G. *et al.* Selective, Modular Probes for Thioredoxins Enabled by Rational Tuning of a Unique Disulfide Structure Motif. *J. Am. Chem. Soc.* **143**, 8791–8803 (2021). https://doi.org/10.1021/jacs.1c03234.

15. Mandal, P. K. *et al.* Loss of Thioredoxin Reductase 1 Renders Tumors Highly Susceptible to Pharmacologic Glutathione Deprivation. *Cancer Res.* **70**, 9505–9514 (2010). https://doi.org/10.1158/0008-5472.CAN-10-1509.

16. Mandal, P. K. Complex Redundancy between the Mammalian Thioredoxin and Glutathione Systems in Cell Proliferation and Tumorigenesis. (Ludwig-Maximilian-University Munich, 2009).

17. Karlenius, T. C., Shah, F., Di Trapani, G., Clarke, F. M. & Tonissen, K. F. Cycling hypoxia up-regulates thioredoxin levels in human MDA-MB-231 breast cancer cells. *Biochem. Biophys. Res. Commun.* **419**, 350–355 (2012). https://doi.org/10.1016/j.bbrc.2012.02.027.

18. Lothrop, A. P., Ruggles, E. L. & Hondal, R. J. No Selenium Required: Reactions Catalyzed by Mammalian Thioredoxin Reductase That Are Independent of a Selenocysteine Residue. *Biochemistry* **48**, 6213–6223 (2009). https://doi.org/10.1021/bi802146w.

19. Zhao, J. *et al.* Loss of thioredoxin reductase function in a mouse stroke model disclosed by a two-photon fluorescent probe. *Chem. Commun.* **56**, 14075–14078 (2020). https://doi.org/10.1039/D0CC05900E.

20. Baell, J. B. & Nissink, J. W. M. Seven Year Itch: Pan-Assay Interference Compounds (PAINS) in 2017—Utility and Limitations. *ACS Chem. Biol.* **13**, 36–44 (2018). https://doi.org/10.1021/acschembio.7b00903.

21. Nelson, K. M. *et al.* The Essential Medicinal Chemistry of Curcumin. *J. Med. Chem.* **60**, 1620–1637 (2017). https://doi.org/10.1021/acs.jmedchem.6b00975.

22. Bisson, J. *et al.* Can Invalid Bioactives Undermine Natural Product-Based Drug Discovery? *J. Med. Chem.* **59**, 1671–1690 (2016). https://doi.org/10.1021/acs.jmedchem.5b01009.

23. Thorne, N., Auld, D. S. & Inglese, J. Apparent activity in high-throughput screening: origins of compound-dependent assay interference. *Mol. Divers.* **14**, 315–324 (2010). https://doi.org/10.1016/j.cbpa.2010.03.020.

24. Stafford, W. C. *et al.* Irreversible inhibition of cytosolic thioredoxin reductase 1 as a mechanistic basis for anticancer therapy. *Sci. Transl. Med.* **10**, eaaf7444 (2018). https://doi.org/10.1126/scitranslmed.aaf7444.

25. Cassini, A. & Messori, L. Molecular Mechanisms and Proposed Targets for Selected Anticancer Gold Compounds. *Curr. Top. Med. Chem.* **11**, 2647–2660 (2011). https://doi.org/10.2174/156802611798040732.

26. Saei, A. A. *et al.* Comprehensive chemical proteomics for target deconvolution of the redox active drug auranofin. *Redox Biol.* **32**, 101491 (2020). https://doi.org/10.1016/j.redox.2020.101491.

27. Snyder, R. M., Mirabelli, C. K. & Crooke, S. T. Cellular interactions of auranofin and a related gold complex with raw 264.7 macrophages. *Biochem. Pharmacol.* **36**, 647–654 (1987). https://doi.org/10.1016/0006-2952(87)90715-5.

28. Albert, A. *et al.* Speciation analysis of the antirheumatic agent Auranofin and its thiol adducts by LC/ESI-MS and LC/ICP-MS. *J. Anal. At. Spectrom.* **27**, 975–981 (2012). https://doi.org/10.1039/C2JA30109A.

29. Coffer, M. T., Shaw, C. F., Hormann, A. L., Mirabelli, C. K. & Crooke, S. T. Thiol competition for Et3PAuS-albumin: a nonenzymatic mechanism for Et3PO formation. *J. Inorg. Biochem.* **30**, 177–187 (1987). https://doi.org/10.1016/0162-0134(87)80062-4.

30. Bachman, R. E., Bodolosky-Bettis, S. A., Pyle, C. J. & Gray, M. A. Reversible Oxidative Addition and Reductive Elimination of Fluorinated Disulfides at Gold(I) Thiolate Complexes: A New Ligand Exchange Mechanism. *J. Am. Chem. Soc.* **130**, 14303–14310 (2008). https://doi.org/10.1021/ja805266r.

31. Reglinski, J., Hoey, S. & Smith, W. E. Exchange reactions between disulphides and myocrisin: An in vitro model for a mechanism in chrysotherapy. *Inorganica Chim. Acta* **152**, 261–264 (1988). https://doi.org/10.1016/S0020-1693(00)91479-8.

32. Thomas, R. C. & Reed, L. J. Disulfide Polymers of DL-α-Lipoic Acid. *J. Am. Chem. Soc.* **78**, 6148–6149 (1956). https://doi.org/10.1021/ja01604a053.

33. Cheng, Y. *et al.* Inhibitors of thiol-mediated uptake. *Chem. Sci.* (2021). doi:10.1039/D0SC05447J. https://doi.org/10.1039/D0SC05447J.

34. Rothan, H. A. *et al.* The FDA- approved gold drug Auranofin inhibits novel coronavirus (SARS-COV-2) replication and attenuates inflammation in human cells. *bioRxiv* 2020.04.14.041228 (2020) doi:10.1101/2020.04.14.041228. https://doi.org/10.1101/2020.04.14.041228.

35. Fromherz, P. & Ruppel, D. Lipid vesicle formation: the transition from open disks to closed shells. *FEBS Lett.* **179**, 155–159 (1985). https://doi.org/10.1016/0014-5793(85)80211-8.

36. Burns, J. A. & Whitesides, G. M. Predicting the stability of cyclic disulfides by molecular modeling: effective concentrations in thiol-disulfide interchange and the design of strongly reducing dithiols. *J. Am. Chem. Soc.* **112**, 6296–6303 (1990). https://doi.org/10.1021/ja00173a017.

37. Blaschke, B. M., Böhm, P., Drieschner, S., Nickel, B. & Garrido, J. A. Lipid Monolayer Formation and Lipid Exchange Monitored by a Graphene Field-Effect Transistor. *Langmuir* **34**, 4224–4233 (2018). https://doi.org/10.1021/acs.langmuir.8b00162.

38. Hu, G. *et al.* Fluorescent Probes for Imaging Protein Disulfides in Live Organisms. *ACS Sens.* **6**, 1384–1391 (2021). https://doi.org/10.1021/acssensors.1c00049.

39. Dóka, É. *et al.* A novel persulfide detection method reveals protein persulfide- and polysulfide-reducing functions of thioredoxin and glutathione systems. *Sci. Adv.* **2**, e1500968 (2016). https://doi.org/10.1126/sciadv.1500968.

40. Cheng, Q. & Arnér, E. S. J. Selenocysteine Insertion at a Predefined UAG Codon in a Release Factor 1 (RF1)-depleted Escherichia coli Host Strain Bypasses Species Barriers in Recombinant Selenoprotein Translation. *J. Biol. Chem.* **292**, 5476–5487 (2017). https://doi.org/10.1074/jbc.M117.776310.

41. Xu, J., Cheng, Q. & Arnér, E. S. J. Details in the catalytic mechanism of mammalian thioredoxin reductase 1 revealed using point mutations and juglone-coupled enzyme activities. *Free Radic. Biol. Med.* **94**, 110–120 (2016). https://doi.org/10.1016/j.freeradbiomed.2016.02.013.

42. Pader, I. *et al.* Thioredoxin-related protein of 14 kDa is an efficient L-cystine reductase and S-denitrosylase. *Proc. Natl. Acad. Sci. U. S. A.* **111**, 6964–6969 (2014). https://doi.org/10.1073/pnas.1317320111.

43. Schindelin, J. *et al.* Fiji: an open-source platform for biological-image analysis. *Nat. Methods* **9**, 676–682 (2012). https://doi.org/10.1038/nmeth.2019.

44. Westerfield, M. *The Zebrafish Book. A Guide for the Laboratory Use of Zebrafish (Danio rerio)*. (University of Oregon Press).

45. Preibisch, S., Saalfeld, S. & Tomancak, P. Globally optimal stitching of tiled 3D microscopic image acquisitions. *Bioinformatics* **25**, 1463–1465 (2009). https://doi.org/10.1093/bioinformatics/btp184.

46. Lukesh, J. C., Palte, M. J. & Raines, R. T. A Potent, Versatile Disulfide-Reducing Agent from Aspartic Acid. *J. Am. Chem. Soc.* **134**, 4057–4059 (2012). https://doi.org/10.1021/ja211931f.

47. Zhou, P., Yao, J., Hu, G. & Fang, J. Naphthalimide Scaffold Provides Versatile Platform for Selective Thiol Sensing and Protein Labeling. *ACS Chem. Biol.* **11**, 1098–1105 (2016). https://doi.org/10.1021/acschembio.5b00856.

48. Ao, X., Bright, S. A., Taylor, N. C. & Elmes, R. B. P. 2-Nitroimidazole based fluorescent probes for nitroreductase; monitoring reductive stress in cellulo. *Org. Biomol. Chem.* **15**, 6104–6108 (2017). https://doi.org/10.1039/C7OB01406F.

49. Sun, Q. *et al.* Non-Peptide-Based Fluorogenic Small-Molecule Probe for Elastase. *Anal. Chem.* **85**, 11304–11311 (2013). https://doi.org/10.1021/ac402097g.
