# Supplementary material for: Cyclic 5-membered disulfides are not selective substrates of thioredoxin reductase, but are opened nonspecifically
Source: Nat Commun. 2022 Apr 1;13:1754. doi: 10.1038/s41467-022-29136-4 (PMC8975869; doi:10.1038/s41467-022-29136-4)
Supplement: Supplementary file 3 — Description of Additional Supplementary Files [file 41467_2022_29136_MOESM3_ESM.pdf]

Description of Additional Supplementary Files

Supplementary Movie 1: Representative confocal time lapse microscopy of live HeLa cells treated with SS50-PQ (50 µM) and immediately imaged for one hour at ex/em: 405/530bp20; fluorescence channel (green) overlaid on brightfield transmission image.
