# Supplementary material for: Cyclic 5-membered disulfides are not selective substrates of thioredoxin reductase, but are opened nonspecifically
Source: Nat Commun. 2022 Apr 1;13:1754. doi: 10.1038/s41467-022-29136-4 (PMC8975869; doi:10.1038/s41467-022-29136-4)
Supplement: Supplementary file 5 — Reporting summary [file 41467_2022_29136_MOESM5_ESM.pdf]

nature research

Corresponding author(s): Oliver Thorn-Seshold

Last updated by author(s): Feb 17, 2022

Reporting Summary

Nature Research wishes to improve the reproducibility of the work that we publish. This form provides structure for consistency and transparency in reporting. For further information on Nature Research policies, see our Editorial Policies and the Editorial Policy Checklist.

Statistics

For all statistical analyses, confirm that the following items are present in the figure legend, table legend, main text, or Methods section.

n/a	Confirmed	
☐	☒	The exact sample size (n) for each experimental group/condition, given as a discrete number and unit of measurement
☐	☒	A statement on whether measurements were taken from distinct samples or whether the same sample was measured repeatedly
☒	☐	The statistical test(s) used AND whether they are one- or two-sided<br>*Only common tests should be described solely by name; describe more complex techniques in the Methods section.*
☒	☐	A description of all covariates tested
☒	☐	A description of any assumptions or corrections, such as tests of normality and adjustment for multiple comparisons
☐	☒	A full description of the statistical parameters including central tendency (e.g. means) or other basic estimates (e.g. regression coefficient) AND variation (e.g. standard deviation) or associated estimates of uncertainty (e.g. confidence intervals)
☒	☐	For null hypothesis testing, the test statistic (e.g. F, t, r) with confidence intervals, effect sizes, degrees of freedom and P value noted<br>*Give P values as exact values whenever suitable.*
☒	☐	For Bayesian analysis, information on the choice of priors and Markov chain Monte Carlo settings
☒	☐	For hierarchical and complex designs, identification of the appropriate level for tests and full reporting of outcomes
☒	☐	Estimates of effect sizes (e.g. Cohen's d, Pearson's r), indicating how they were calculated

Our web collection on statistics for biologists contains articles on many of the points above.

Software and code

Policy information about availability of computer code

Data collection	Flow Cytometry data was collected on a BD LSRFortessa instrument from BD Bioscience, Heidelberg (Germany) using the integrated BD FACSDiva v. 8.0.1 software.<br>Plate reader data were measured on FLUOstar Omega (BMG Labtech) and collected using Omega Software version 5.50 R3 (BMG Labtech) or else on a Tecan Infinite M200 plate reader (integrated software) from Tecan, Maennedorf (Switzerland).<br>Image acquisition for zebrafish embryos was controlled by the Zen Black software v. 2.5. from Carl Zeiss microscopy, Jena, Germany for confocal imaging, and Las X software v. 5.0.3. for stereofluorescence imaging (Leica Microsystems, Wetzlar, Germany). Tile stitching was performed in ImageJ using the open-source Fiji plugin version 1.51.
Data analysis	Dose-response curves were fitted using GraphPad Prism version 8.0.2 for Windows<br>Flow Cytometry data was gated and analyzed using FlowJO v.10.7.1 (BD Biosciences)<br>Live cell imaging data and zebrafish embryo data was adjusted for brightness and contrast using ImageJ with the open-source Fiji plugin version 1.51 (cf. Preibisch et al. Bioinformatics 2009).<br>NMR spectra were analysed with the software MestreNova 12 developed by MestreLab Ltd., Santiago de Compostela (Spain).

For manuscripts utilizing custom algorithms or software that are central to the research but not yet described in published literature, software must be made available to editors and reviewers. We strongly encourage code deposition in a community repository (e.g. GitHub). See the Nature Research guidelines for submitting code & software for further information.

Data

Policy information about availability of data

All manuscripts must include a data availability statement. This statement should provide the following information, where applicable:

- Accession codes, unique identifiers, or web links for publicly available datasets
- A list of figures that have associated raw data
- A description of any restrictions on data availability

All data generated or analysed during this study are included in this article and its Supplementary Information files, including the Source Data file (raw data for Fig. 3-6 and Supplementary Fig. 3-16). These and all data of this study can also be obtained from the authors upon request. None of these datasets are resources of public interest and therefore are not archived publicly in other forms

Field-specific reporting

Please select the one below that is the best fit for your research. If you are not sure, read the appropriate sections before making your selection.

- [x] Life sciences
- [ ] Behavioural & social sciences
- [ ] Ecological, evolutionary & environmental sciences

For a reference copy of the document with all sections, see nature.com/documents/nr-reporting-summary-flat.pdf

Life sciences study design

All studies must disclose on these points even when the disclosure is negative.

Sample size	Number of cells used in the assays were chosen according to common practice and cell numbers are available in the method section or in the supporting information. Sample size was mainly chosen by the appropriate culture vessel format experiments were run in, considering practicability and ability to run ideally all samples in parallel. Cells were grown to 80-90% confluency in the respective vessel. All assays are given additional validity by being run as time courses and/or titration assays. For microscopy cell density was chosen to be around 50-70% confluency to show the reader enough cells, while cells are still surrounded by clear background. For flow cytometry we chose at least 10000 analyzable events to give a reliable picture of probe activation.
Data exclusions	No data was excluded from the presented studies; experiments were only interpreted, if all controls ran as expected according to standardized procedures described in the Supporting Information. For example, as described in the main text, we performed "reducibility benchmark tests" with stocks of dithiolane probes (like SS50PQ) by reducing them with TCEP, and only if the fluorescence obtained matched expectations for the maximum signal for that concentration were assays with that stock interpreted, otherwise it was concluded that the stock had polymerised or otherwise degraded and a fresh stock was prepared from solid and assays were redone.<br>All animals included in the study were analyzed, and representative examples are shown.
Replication	All attempts at replications were successful, unless stated otherwise. All relevant assays were done at least three times independently according to common practice. Zebrafish assays: Experiments involving zygotes and 3 dpf embryos were carried out in triplicates. In addition, two replicates were carried out for monitoring of signal development for 3 dpf embryos using confocal imaging. For each of the replicates, eggs from a different clutch were used.
Randomization	No randomization necessary for the cell culture experiments in this study and all experiments with all probes were run on the same cell lines. The experiments have been designed according to common practice and frequently checked for systematic errors or the influence of covariates. Zebrafish assays: As there is no established way of discriminating between zebrafish embryos at this early developmental stage (e.g. by sex), all embryos included in this study were randomly allocated to the test groups.
Blinding	No blinding necessary for the cell culture experiments in this study: quantitative assays used for probe validation are automatically performed by machines and software and are therefore considered objective. Furthermore, cellular and cellfree assays were independently run by different experimenters (typically in both the labs at Karolinska and in Munich) and results were similar.

Reporting for specific materials, systems and methods

We require information from authors about some types of materials, experimental systems and methods used in many studies. Here, indicate whether each material, system or method listed is relevant to your study. If you are not sure if a list item applies to your research, read the appropriate section before selecting a response.

Materials & experimental systems

n/a	Involved in the study
x	Antibodies
	x Eukaryotic cell lines
x	Palaeontology and archaeology
	x Animals and other organisms
x	Human research participants
x	Clinical data
x	Dual use research of concern

Methods

n/a	Involved in the study
x	ChIP-seq
	x Flow cytometry
x	MRI-based neuroimaging

Eukaryotic cell lines

Policy information about cell lines

Cell line source(s)	HeLa (DSMZ; ACC 57), Jurkat T-Cells (ATCC, TIB-152), A549 (DSMZ, ACC 107) from DSMZ. MEF fl/fl (for TrxR1), MEF -/- "Knock-out" (for TrxR1), MEF 2ATG "Knock-in" (for TrxR1) from Marcus Conrad's laboratory (Helmholtz Centre, Munich; reference 70 in the paper, Mandal, P. K. et al. Loss of Thioredoxin Reductase 1 Renders Tumors Highly Susceptible to Pharmacologic Glutathione Deprivation. Cancer Res 70, 9505–9514 (2010). https://doi.org/10.1158/0008-5472.CAN-10-1509 AND reference 71, PhD thesis, Mandal, P. K. Complex Redundancy between the Mammalian Thioredoxin and Glutathione Systems in Cell Proliferation and Tumorigenesis. (Ludwig-Maximilian-University Munich, 2009). https://edoc.ub.uni-muenchen.de/11958/1/Mandal_Pankaj_Kumar.pdf). The MEF 2ATG line was additionally validated by us for reconstitution of Sec-containing TrxR1 in 2014, see 10.1038/cddis.2014.209. Additional summary information on the derivation of the MEF lines is given in the Main and Supporting texts
Authentication	Cell lines were not specifically authenticated for this study, other than monitoring microscopic appearance and general properties daily. HeLa, Jurkat T-Cells, and A549 were recently purchased from ATCC and run through regular replacement cycles from our master cell bank.
Mycoplasma contamination	all cell lines in this studies were mycoplasma negative
Commonly misidentified lines (See ICLAC register)	none

Animals and other organisms

Policy information about studies involving animals; ARRIVE guidelines recommended for reporting animal research

Laboratory animals	The study included Danio rerio embryos (up to 3 dpf, sex undetermined) of wt background (ab). (Wilson et al. Genetics 198, 1291; doi:10.1534/genetics.114.169284 (2014)) Fish were cared for according to best practice guides (Westerfield, M. 2007. The Zebrafish Book. A Guide for the Laboratory Use of Zebrafish (Danio rerio). University of Oregon Press. 5th edition)
Wild animals	No wild animals were used in this study.
Field-collected samples	The study did not include animals collected in the wild.
Ethics oversight	As all zebrafish embryos in this study were analyzed at ages below 5 days post fertilization, hence no approval by an ethics board was required, in agreement with German legislation. The study adhered to ARRIVE guidelines.

Note that full information on the approval of the study protocol must also be provided in the manuscript.

Flow Cytometry

Plots

Confirm that:

- [x] The axis labels state the marker and fluorochrome used (e.g. CD4-FITC).
- [x] The axis scales are clearly visible. Include numbers along axes only for bottom left plot of group (a 'group' is an analysis of identical markers).
- [x] All plots are contour plots with outliers or pseudocolor plots.
- [x] A numerical value for number of cells or percentage (with statistics) is provided.

Methodology

Sample preparation	Jurkat cells were seeded 24 h before treatment with inhibitors then incubated for at least 3 h more before treatment with SS-50-PQ

Instrument	BD LSRFortessa
Software	Data collection: BD FACSDiva 8.0.1<br>Data analysis: FlowJo software v.10.7.1 (BD Biosciences)
Cell population abundance	1) gating for main cell population at least 5000 cells<br>2) single cell gating at least 3000 cells<br>3) live cell gating at least 2500 cells
Gating strategy	1) FSC-A vs. SSC-A<br>2) FSC-A vs. FSC-H<br>3) Zombie negative and positive gaiting<br>4) PQ positive and negative gaiting

☒ Tick this box to confirm that a figure exemplifying the gating strategy is provided in the Supplementary Information.
